# TRIM21 promotes colorectal cancer development through regulating DNA replication by TCF3/MCM2/5 axis

**DOI:** 10.1038/s41420-025-02722-3

**Published:** 2025-09-25

**Authors:** Xintian Zhang, Han Yao, Yichao Hou, Kun Zhou, Yu Liang, Lidan Hou, Xingming Zhang, Wenfeng Wang, Leilei Du, Mengfei Yao, Jianhua Wang, Xiangjun Meng

**Affiliations:** 1 Shanghai Key Laboratory of Gut Microecology and Associated Major Diseases Research, Digestive Disease Research and Clinical Translation Center, Department of Gastroenterology, Shanghai Ninth People’s Hospital, Shanghai Jiao Tong University School of Medicine, 639 Zhi Zao Ju Road, 200011 Shanghai, China; 2 Cancer Institute, Fudan University Shanghai Cancer Center, Department of Oncology, Shanghai Medical College, Fudan University, 270 Dongan Road, 200032 Shanghai, China

**Keywords:** Gastrointestinal cancer, Cell growth

## Abstract

Disrupting DNA replication has been employed for treating cancers. In the present study, we found that Tripartite motif containing 21 (TRIM21) was highly expressed in colorectal cancer (CRC) and could be valuable for predicting the prognosis of CRC patients. Further study demonstrated that TRIM21 positively regulated the expression of MCM2 and MCM5, DNA replication and proliferation of CRC cells both in vitro and in vivo. In addition, TRIM21 knockdown inhibited both replication initiation and velocity, and increased the chemosensitivity of CRC cells to 5-FU and SN-38. Our study also revealed that DNA replication inhibition following TRIM21 knockdown could not be restored by cell cycle checkpoint kinase inhibitors, but partially by Transcription Factor 3 (TCF3) knockdown. TCF3 directly suppressed *MCM2* and *MCM5* transcription, inhibiting DNA replication. In summary, TRIM21 could influence tumor development and chemosensitivity to replication inhibitors by regulating DNA replication through the TCF3/MCM2/5 axis, suggesting a promising potential for CRC in the clinic.

## Introduction

Colorectal cancer (CRC) is one of the malignant tumors with increasing morbidity and mortality rates [1]. Clinically, strategies in dealing CRC including surgery, chemotherapy, and immunotherapy, et al., but encounter multiple challenges such as loss of surgery opportunity when diagnosed, multiple drug resistance and immunotherapy tolerance et al. [2]. Therefore, insight into the tumorigenesis and development of CRC will provide promising therapeutic targets for CRC.

DNA replication is a tightly regulated process that involves the coordination of many protein molecules [3, 4]. Many of these proteins participate in origin “licensing” and “firing”, and have been found highly expressed in many tumors, and many of them are associated with the outcomes. For example, the expression of Origin Recognition Complex Subunit 1 (ORC1) could be employed to predict the prognosis of many tumors [5]. Aberrant expression of Chromatin Licensing and DNA Replication Factor 1 (CDT1) in colorectal adenomas increased the genomic instability, hence promoted CRC progression [6]. Decreased expression of Mini-chromosome Maintenance Complex Component 3 (MCM3), MCM4, MCM5 and MCM7 inhibited DNA replication and proliferation of various tumor cells [7–9]. MCM2 and MCM3 were more sensitive, specific and efficient proliferation markers for CRC and breast cancer than the Marker of Proliferation Ki-67 (MKI67) [10, 11].

Replication stress (RS) is a major challenge for maintaining genome stability. RS can be induced by inhibitors of DNA replication or aberrant expression of DNA replication proteins et al. [4, 12–17]. Normally, about 10%-30% of “licensed” replication origins will eventually be “fired” while most of the others remain dormant. Upon RS occurrence, these dormant origins will be “fired”, ensuring replication completion [18, 19]. If the number of “licensed” origins decreases, the dormant origins must also decrease, leading to insufficient DNA replication, especially in the presence of RS [12, 16, 20]. Generally, partial deletion of MCMs (MCM2-7) is tolerable under normal conditions, but it will result in replication fork collapse and ultimately cell death in the presence of concurrent replication inhibitors [21, 22]. Therefore, interfering with DNA replication has been considered as an approach to improve the sensitivity of tumor cells to replication inhibitors, and reducing the number of “licensed” origins by down-regulating replication-associated proteins is a major option [15, 17, 23–28].

Tripartite motif containing 21 (TRIM21) belongs to the TRIM protein family and is encoded by *TRIM21* gene located on human chromosome 11p15.4. Human TRIM21 protein was first cloned in 1991 with a molecular weight of 52 kDa and contains 475 amino acids [29]. Most studies on TRIM21 have focused on immune regulation, suggesting that it can promote or inhibit innate immunity [30–32]. However, there are few studies on TRIM21 and tumors, especially in the field of CRC research, and there is no report on its regulation of DNA replication of eukaryotic cells.

In this paper, we reported that TRIM21 positively regulated DNA replication and proliferation of CRC cells through Transcription Factor 3 (TCF3). Suppressing TRIM21 expression could enhance the sensitivity of CRC cells to chemotherapeutic drugs interfering with DNA synthesis. These new findings might provide promising alternative options for CRC in clinic.

## Results

### TRIM21 level was correlated with clinicopathological features and prognosis of CRC patients

**Fig. 1:**
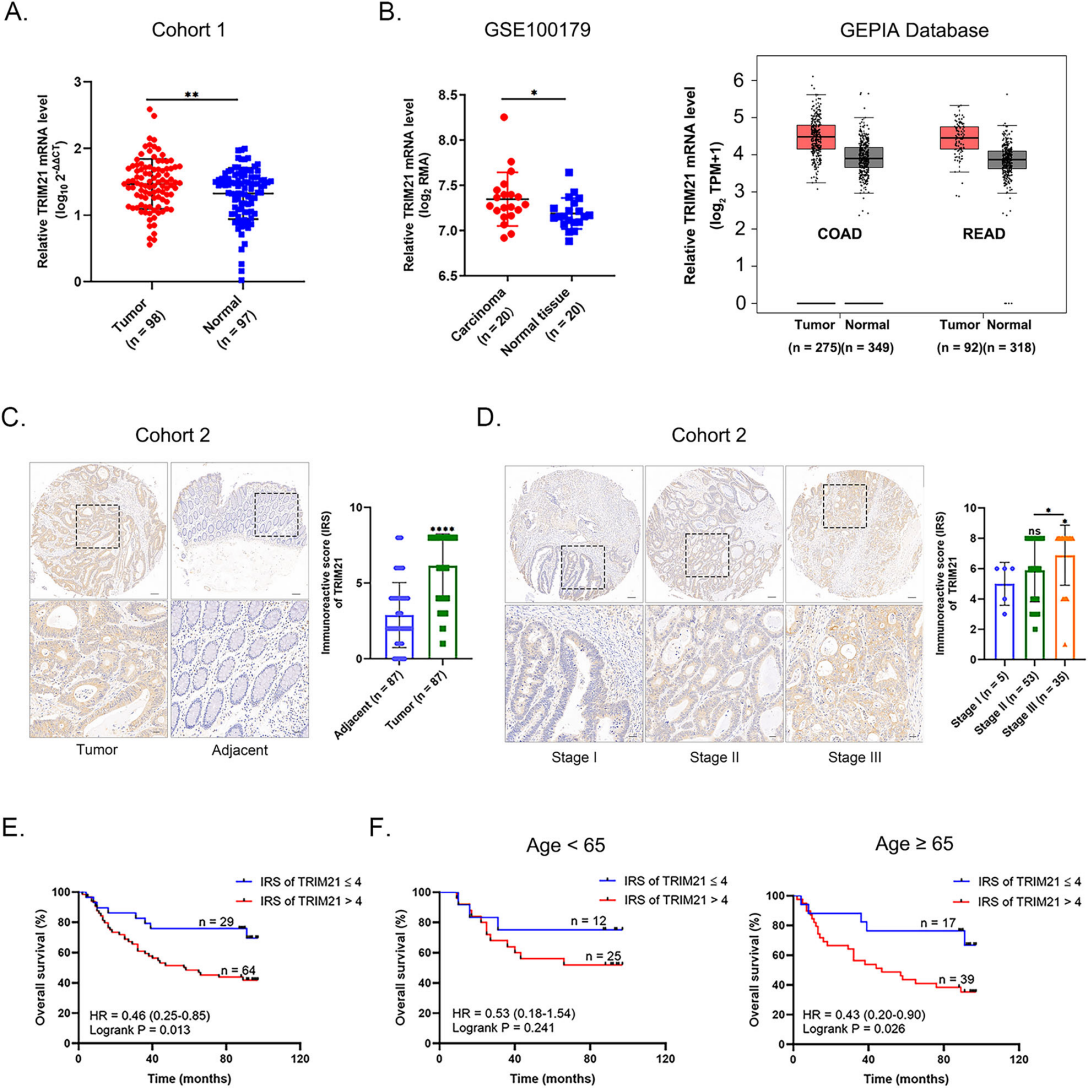
TRIM21 level was correlated with clinicopathological features and prognosis of CRC patients. **A** Relative TRIM21 mRNA level in human CRC tissues (*n* = 98) and normal intestinal epithelium (*n* = 97) from Cohort 1. **P < 0.01. **B** Relative TRIM21 mRNA level in human CRC tissues (*n* = 20) and normal intestinal epithelium (*n* = 20) from the GSE100179 dataset (left panel). Relative TRIM21 mRNA level in COAD (Tumor, *n* = 275; Normal, *n* = 349) and READ (Tumor, *n* = 92; Normal, *n* = 318) datasets from GAPIA Database (right panel). *P < 0.05. **C** Representative IHC staining images and immunoreactive scores of TRIM21 from CRC (*n* = 87) and adjacent normal tissues (*n* = 87) in Cohort 2. Scale bars = 100 μm (upper panel) and 25 μm (lower panel). ****P < 0.0001. **D** Representative IHC staining images and immunoreactive scores of TRIM21 from CRC tissues at different AJCC stages (Stage Ⅰ, *n* = 5; Stage Ⅱ, *n* = 53; Stage Ⅲ, *n* = 35) in Cohort 2. Scale bars = 100 μm (upper panel) and 25 μm (lower panel). *P < 0.05, ns = no significance. **E** Kaplan–Meier overall survival curves for all CRC patients (*n* = 93) in Cohort 2 based on TRIM21 level. **F** Kaplan–Meier overall survival curves for CRC patients aged less than 65 years (left panel, *n* = 37) and those aged 65 years or more (right panel, *n* = 56) in Cohort 2 based on TRIM21 level.

In Cohort 1, TRIM21 mRNA level was significantly higher in CRC tissues than that in normal tissues, consistent with the results from the NCBI Gene Expression Omnibus (GEO) database (GSE100179) and GEPIA database (http://gepia.cancer-pku.cn/) (Fig. 1A, B). Furthermore, immunohistochemical (IHC) staining on human CRC tissue microarray (Cohort 2) demonstrated no significant difference in the distribution of clinicopathological characteristics between groups with low (immunoreactive score [IRS] ≤4) and high (IRS > 4) TRIM21 expression (Supplementary Table 1). Analysis of IHC staining revealed that TRIM21 protein level was significantly higher in CRC tissues than that in paired adjacent normal tissues (Fig. 1C). In addition, TRIM21 protein level in AJCC stage Ⅲ tissues was obviously higher than that in stage Ⅰ and stage Ⅱ tissues, and the IRS of stage Ⅱ tissues was also higher than stage Ⅰ tissues, although there was no statistical difference (Fig. 1D). Besides, Kaplan–Meier survival analysis showed that TRIM21 protein level was negatively correlated with overall survival (OS) of CRC patients, especially those aged ≥65 years (Fig. 1E, F). In general, TRIM21 is expressed higher in CRC tissues than in normal tissues, with higher TRIM21 protein levels predicting poor prognosis, particularly in CRC patients aged 65 years or older.

### TRIM21 regulated CRC cell proliferation and was associated with DNA replication

**Fig. 2:**
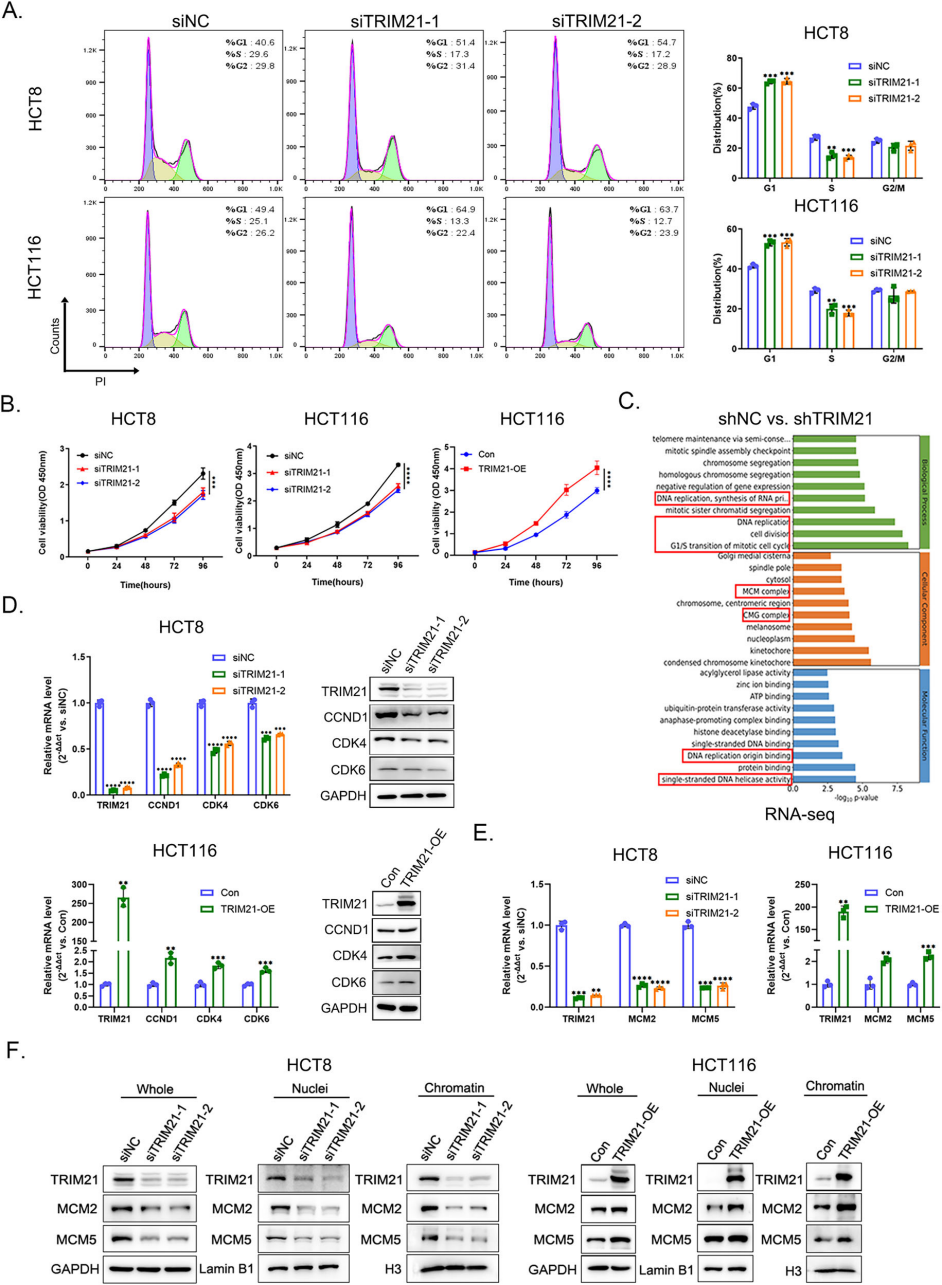
TRIM21 regulated CRC cell proliferation and was associated with DNA replication. **A** Cell cycle analysis of CRC cells with or without TRIM21 knockdown by flow cytometry. **P < 0.01, ***P < 0.001. **B** Cell proliferation was evaluated by CCK-8 assay in CRC cells with TRIM21 knockdown or overexpression. ***P < 0.001, ****P < 0.0001. **C** Gene ontology analysis and functional annotation of differentially expressed genes from RNA-seq results between two groups (shNC and shTRIM21, *n* = 3). These differentially expressed genes were mainly involved in G1-S transition and DNA replication (red boxes). **D** Detection of mRNA and protein levels of CCND1, CDK4 and CDK6 after TRIM21 knockdown or overexpression in CRC cells by RT-qPCR and western blot, respectively. GAPDH was used as a loading control. **P < 0.01, ***P < 0.001, ****P < 0.0001. **E** Detection of MCM2 and MCM5 mRNA levels after TRIM21 knockdown or overexpression in CRC cells by RT-qPCR. **P < 0.01, ***P < 0.001, ****P < 0.0001. **F** Detection of MCM2 and MCM5 protein levels after TRIM21 knockdown or overexpression in whole cell lysates, nuclei and chromatin fractions of CRC cells. GAPDH was used as a loading control for whole cell lysates, Lamin B1 and histone H3 were used as loading controls for nuclei and chromatin fractions, respectively.

Following detection of TRIM21 expression across multiple CRC cell lines (Supplementary Fig. 1A, B), we selected HCT8 and HCT116 cells for subsequent experiments based on their representative expression profiles. Knockdown and overexpression efficiencies were validated as shown in Supplementary Fig. 1C. Cell cycle analysis showed that TRIM21 knockdown induced G1 phase arrest and reduced the proportion of cells in S phase in both cell lines (Fig. 2A). CCK-8 assay revealed that TRIM21 knockdown significantly inhibited CRC cell proliferation, while its overexpression promoted cell growth (Fig. 2B). Gene Ontology analysis of RNA sequencing (RNA-seq) data identified significant enrichment of differentially expressed genes ( | Fold Change | ≥ 1.5, q-value < 0.05) in G1-S phase transition and DNA replication pathways between control and TRIM21-knockdown HCT8 cells (Supplementary Fig. 1D, Fig. 2C). Compared with the control group, CCND1, CDK4 and CDK6 expression were significantly down-regulated in TRIM21 knockdown cells, while upregulated in TRIM21 overexpression cells (Fig. 2D). In terms of Cellular Component, Gene Ontology analysis enriched MCM and Cdc45-MCM-GINS (CMG) complexes, which are crucial for origin “licensing” and “firing” [3, 4]. MCM2 and MCM5, two essential components of MCM and CMG complexes, were positively regulated by TRIM21 in both nuclei and chromatin fractions (Fig. 2E, F). These results indicated that TRIM21 played a positive role in CRC cell proliferation and DNA replication.

### TRIM21 regulated DNA replication in CRC cells

**Fig. 3:**
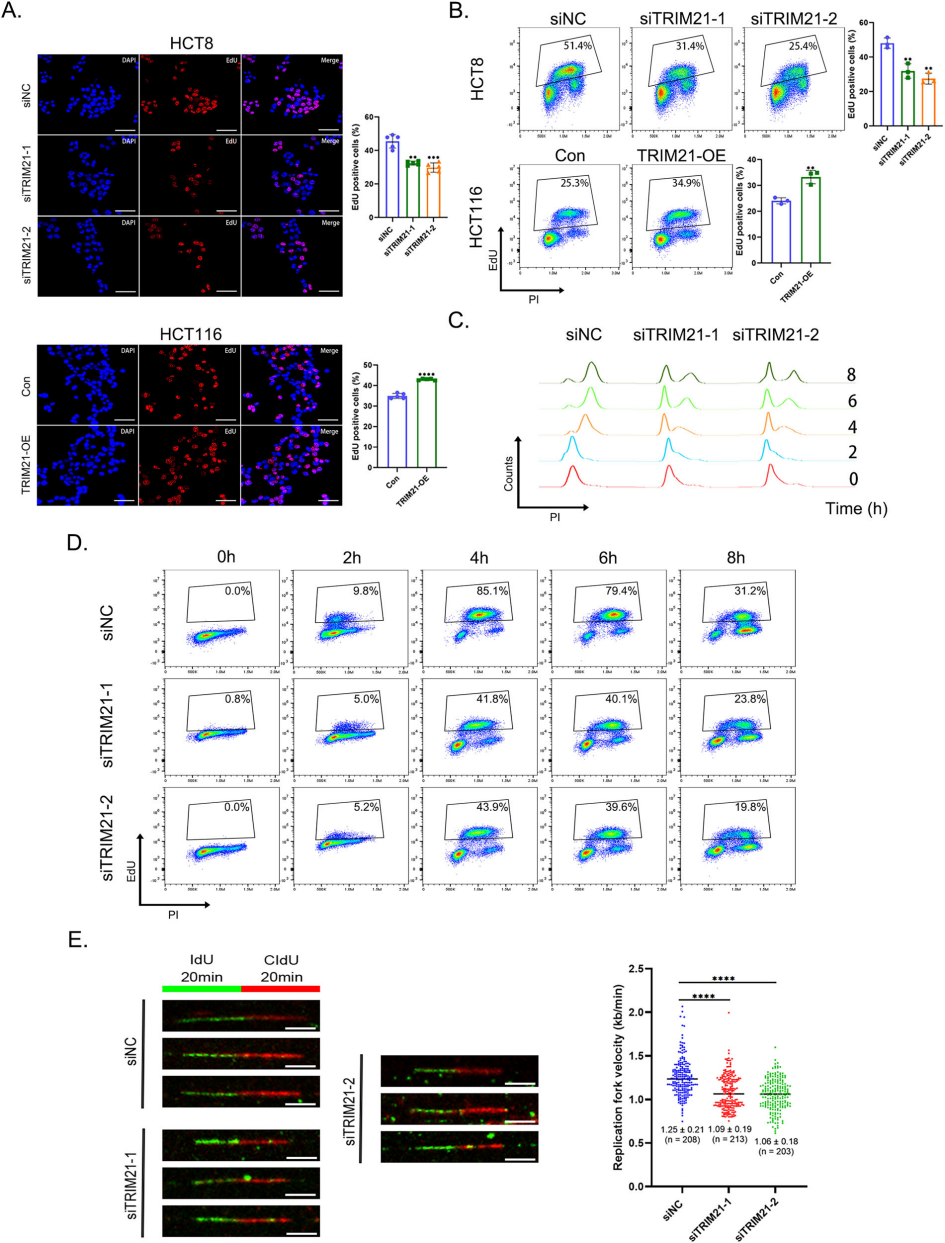
TRIM21 regulated DNA replication in CRC cells. **A** Fluorescence detection of EdU-positive cells in CRC cells with TRIM21 knockdown or overexpression. Nuclei were stained with DAPI (blue), and replicating DNA was incorporated with EdU (red). Scale bars = 50 μm. **P < 0.01, ***P < 0.001, ****P < 0.0001. **B** Detection of EdU-positive cells in CRC cells with TRIM21 knockdown or overexpression by EdU/PI double staining assay. **P < 0.01. **C** Cell cycle analysis of HCT8 cells collected at different time points (0 h, 2 h, 4 h, 6 h, 8 h) after release of synchronization from siNC group and siTRIM21 groups by flow cytometry. **D** Detection of EdU-positive cells in HCT8 cells collected at different time points (0 h, 2 h, 4 h, 6 h, 8 h) after release of synchronization from siNC group and siTRIM21 groups by EdU/PI double staining assay. **E** Results of DNA fiber assay from HCT8 cells with or without TRIM21 knockdown. The representative images were shown in the left panel. Green fluorescence represented IdU incorporated DNA fibers and red fluorescence represented CIdU incorporated DNA fibers. Scale bars = 5 μm. Analysis of replication fork velocity was presented in the right panel. ****P < 0.0001.

Verifying the effect on DNA replication, we found the proportion of EdU-positive cells in the TRIM21 knockdown group was significantly lower than that in the control group, and vice versa (Fig. 3A, B and Supplementary Fig. 2A, B). It is reported that the MCM2/5 gate conformation regulates DNA helicase activity, and their zinc-finger rings were essential for DNA unwinding and replication initiation [28, 33, 34]. Given the importance of MCM2 and MCM5 in promoting DNA replication, we then explored the effect of TRIM21 on replication initiation and elongation. Cells were synchronized at the G1/S boundary by double thymidine block [35] (Supplementary Fig. 2C, D). Cell cycle analysis revealed that TRIM21 depletion significantly impaired S-phase entry following synchronization release, suggesting its role in regulating replication initiation (Fig. 3C). The flow cytometry analysis displayed a more intuitive result (Fig. 3D). Besides, DNA fiber assay was used to examine replication elongation and we found the DNA replication rate was slowed down in siTRIM21 group, and the length of newly synthesized DNA fibers was shortened compared with the control group (Supplementary Fig. 2E, Fig. 3E). Taken together, these findings demonstrated the importance of TRIM21 in facilitating DNA replication in CRC cells, suggesting its role in supporting CRC progression.

### Temporal and spatial distribution of TRIM21 protein

Our immunoblotting data in Fig. 2F displayed that TRIM21 can be detected both in nuclei and chromatin fractions. Immunofluorescence staining also confirmed the distribution of TRIM21 in cytoplasm and nuclei (Supplementary Fig. 3A). In addition, TRIM21 protein was expressed in all phases of the cell cycle without differences in abundance in whole cell lysates. However, TRIM21 expression peaked at G1/S boundary (0 h) and early-mid S phase (2nd to 4th hour post-release) in nuclei, and decreased upon G2 transition initiation ( ≥ 6 h) (Supplementary Fig. 3B). Similarly, TRIM21, MCM2 and MCM5 levels at 0, 2nd, and 4th hour after release in chromatin was higher than those at 6th hour (Supplementary Fig. 3C). The relatively high expression of TRIM21 protein in nuclei and chromatin during G1/S boundary and S phase might be associated with its putative role in DNA replication regulation. To achieve higher spatiotemporal resolution, we performed Isolation of proteins on nascent DNA (iPOND) assay (Supplementary Fig. 3D). Results showed that TRIM21 protein was highly abundant in pulse samples but not in chase samples, indicating that TRIM21 protein might be present around replication forks (Supplementary Fig. 3E). These results provided spatial and temporal evidence for the effect of TRIM21 protein in regulating DNA replication.

### TRIM21 knockdown enhanced the chemosensitivity of CRC cells

**Fig. 4:**
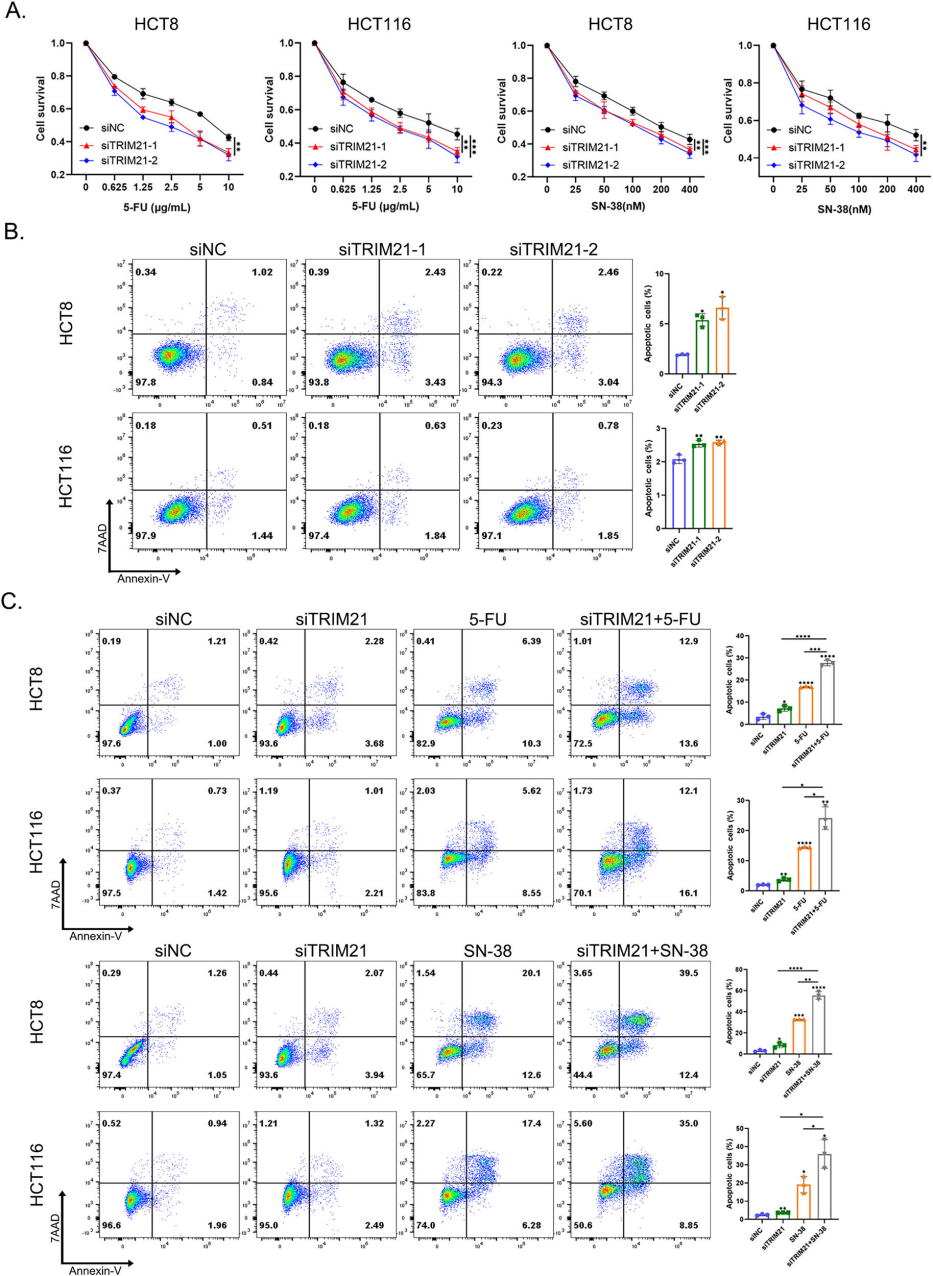
TRIM21 knockdown enhanced the chemosensitivity of CRC cells. **A** Survival of CRC cells with or without TRIM21 knockdown after treatment with different concentrations of 5-FU or SN-38. **P < 0.01, ***P < 0.001. **B** Apoptosis analysis of CRC cells with or without TRIM21 knockdown by 7AAD/Annexin-V staining. *P < 0.05, **P < 0.01. **C** Apoptosis analysis of CRC cells with or without TRIM21 knockdown after treatment with a relatively low concentration 5-FU (0.625 μg/mL) or SN-38 (25 nM) by 7AAD/Annexin-V staining. *P < 0.05, **P < 0.01, ***P < 0.001, ****P < 0.0001.

As mentioned above, decreased levels of replication-associated proteins would result in fewer origin “licensing” events, potentially increasing tumor cell susceptibility to replication inhibitors [12, 16, 20]. Two DNA replication inhibitors, 5-FU and SN-38, were used in our study [36, 37]. The present results showed that TRIM21 knockdown enhanced the inhibitory effect of different concentrations of 5-FU or SN-38 on CRC cell proliferation (Fig. 4A). The combination of a relatively low concentration 5-FU (0.625 μg/mL) or SN-38 (25 nM) and TRIM21 knockdown further increased the number of apoptotic cells, with a more significant effect than TRIM21 knockdown alone or replication inhibitor treatment alone (Fig. 4B, C). Some experiments were also conducted in a 5-FU-resistant cell line (HCT8-5FU), but the inhibitory effect on HCT8-5FU cells after TRIM21 knockdown was not that obvious (Supplementary Fig. 4A–D).

### Regulation of DNA replication by TRIM21 was independent of the DNA damage response

DNA damage activates DNA damage response (DRR), during which the cell cycle will be transiently arrested to repair these damages [38]. γH2AX is often used as a marker for double-strand breaks [39] and was significantly increased in CRC cells following hydroxyurea (HU) treatment, but no change was detected at each time point after TRIM21 knockdown compared to control cells (Supplementary Fig. 5A). Two groups of cell cycle checkpoint kinase, ATM/ATR and CHK1/CHK2, played important roles in DDR [40, 41]. Notably, neither UCN-01 (a CHK1 inhibitor, 500 nM) nor caffeine (an ATM/ATR inhibitor, 5 mM) [38] reversed the reduction in EdU-positive cells caused by TRIM21 knockdown (Supplementary Fig. 5B). In addition, CHK1 activation depends on its phosphorylation at Ser317 and Ser345 [42]. HU treatment increased CHK1 (Ser345) phosphorylation level, whereas TRIM21 knockdown showed no significant effect on CHK1 phosphorylation in either HU untreated or treated CRC cells (Supplementary Fig. 5C). Therefore, we illustrated that TRIM21 regulated DNA replication in CRC cells through a DDR-independent mechanism.

### TCF3 regulated DNA replication of CRC cells

**Fig. 5:**
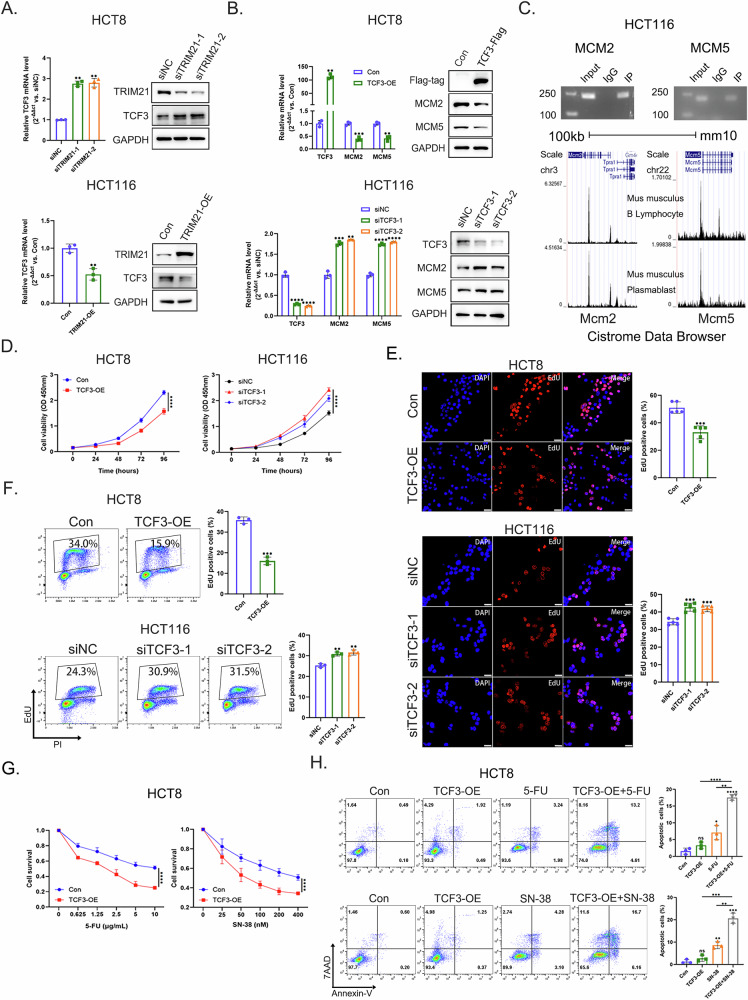
TCF3 regulated DNA replication of CRC cells. **A** Detection of mRNA (left panel) and protein (right panel) levels of TCF3 after TRIM21 knockdown or overexpression in CRC cells, respectively. GAPDH was used as a loading control. **P < 0.01. **B** Detection of mRNA (left panel) and protein (right panel) levels of MCM2 and MCM5 after TCF3 overexpression or knockdown in CRC cells, respectively. GAPDH was used as a loading control. **P < 0.01, ***P < 0.001, ****P < 0.0001. **C** ChIP assay (upper panel) and ChIP-seq (lower panel) results of TCF3 binding in the promoter regions of *MCM2* and *MCM5*. ChIP-seq results derived from mouse plasma blast cells and B lymphocytes were obtained from the Cistrome Data Browser. **D** Cell proliferation was evaluated by CCK-8 assay in CRC cells with TCF3 overexpression or knockdown. ****P < 0.0001. **E** Fluorescence detection of EdU-positive cells in CRC cells with TCF3 overexpression or knockdown. Nuclei were stained with DAPI (blue), and replicating DNA was incorporated with EdU (red). Scale bars = 50 μm. ***P < 0.001. **F** Detection of EdU-positive cells in CRC cells with TCF3 overexpression or knockdown by EdU/PI double staining assay. **P < 0.01, ***P < 0.001. **G** Survival of HCT8 cells with or without TCF3 overexpression after treatment with different concentrations of 5-FU or SN-38. ****P < 0.0001. **H** Apoptosis analysis of HCT8 cells with or without TCF3 overexpression after treatment with a relatively low concentration 5-FU (0.625 μg/mL) or SN-38 (25 nM) by 7AAD/Annexin-V staining. *P < 0.05, **P < 0.01, ***P < 0.001, ****P < 0.0001, ns = no significance.

As TRIM21 regulated MCM2 and MCM5 mRNA levels in our study, we examined the transcriptional regulation of *MCM2* and *MCM5* by TRIM21 through transcription factors. Taking advantage of the Mean Rank of the chEA3 database (https://maayanlab.cloud/chea3/) and our RNA-seq results, we then focused on TCF3. TCF3 expression was negatively regulated by TRIM21, and it also exerted a negative regulatory effect on MCM2 and MCM5 expression (Fig. 5A, B). Chromatin immunoprecipitation (ChIP) assay demonstrated direct binding of TCF3 protein to the *MCM2*/*MCM5* gene promoter region (Fig. 5C, upper panel), and ChIP-seq results of mouse plasma blast cells and B lymphocytes [43] from Cistrome Data Browser (http://cistrome.org/db/#/) also displayed the same result (Fig. 5C, lower panel). Moreover, luciferase reporter assay confirmed that TCF3 overexpression suppressed wild-type (WT) *MCM2* reporter activity but had no effect on the mutant construct (*MCM2* MT1) with disrupted TCF3-binding sites. Similarly, while TCF3 strongly repressed WT *MCM5* reporter activity, the mutant (*MCM5* MT3) exhibited only partial inhibition (Supplementary Fig. 6A, B). These results demonstrated that TCF3 repressed *MCM2* and *MCM5* transcription through direct promoter interactions.

We also explored whether TCF3 could function as a substrate of TRIM21, but the Co-Immunoprecipitation (Co-IP) experiment did not show any interaction between the two (Supplementary Fig. 6C). Overexpression of an E3 ligase-dead mutant TRIM21 (C16A) [44, 45] in HCT116 cells had the same regulatory effect as wild-type TRIM21 did on MCM2, MCM5 and TCF3 expression (Supplementary Fig. 6D), indicating that the E3 ligase function of TRIM21 might not be involved in it. Besides, we excluded the possibility of direct transcriptional regulation of *TCF3* by TRIM21, as the ChIP assay failed to detect TRIM21 binding at *TCF3* promoter region (Supplementary Fig. 6E).

When studying TCF3’s effects on DNA replication in CRC cells, we found that TCF3 overexpression inhibited cell proliferation and reduced the percentage of EdU-positive cells, while TCF3 knockdown had the opposite results (Fig. 5D–F). We also observed that TCF3 overexpression enhanced the inhibitory effect of 5-FU and SN-38 on cell proliferation, as well as promoted apoptosis of cells treated with a relatively low concentration 5-FU (0.625 μg/mL) or SN-38 (25 nM) (Fig. 5G, H). Hence, TCF3 acted downstream of TRIM21 to inhibit DNA replication as well as the proliferation of CRC cells.

### TCF3 downregulation partially restored the inhibition of DNA replication caused by TRIM21 knockdown

**Fig. 6:**
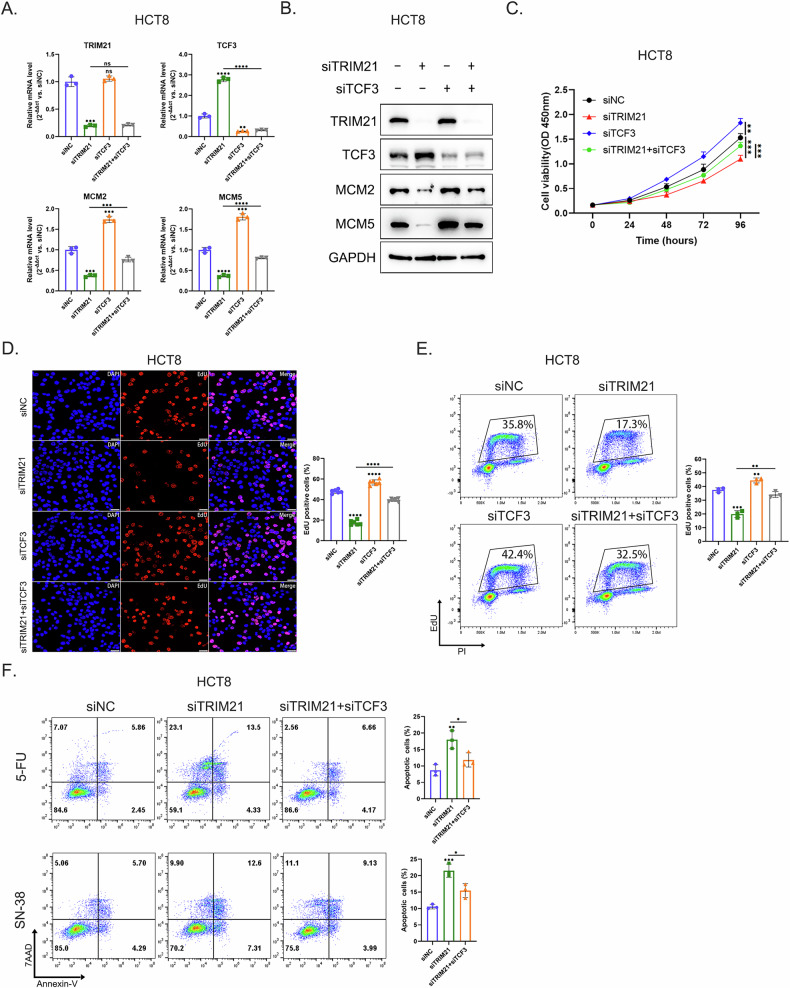
TCF3 downregulation partially restored the inhibition of DNA replication caused by TRIM21 knockdown. **A**, **B** Decreased MCM2 and MCM5 mRNA (**A**) and protein (**B**) levels were partially rescued by TCF3 knockdown after TRIM21 downregulation in HCT8 cells. GAPDH was used as a loading control. **P < 0.01, ***P < 0.001, ****P < 0.0001, ns = no significance. **C** After TRIM21 knockdown, TCF3 downregulation partially reversed the proliferation restriction in HCT8 cells. **P < 0.01, ***P < 0.001. **D**, **E** TCF3 knockdown restored the decrease in the percentage of EdU-positive HCT8 cells caused by TRIM21 downregulation to a certain extent. Figure 6D showed the results of fluorescence detection. Nuclei were stained with DAPI (blue), and replicating DNA was incorporated with EdU (red). Scale bars = 50 μm. Figure 6E displayed the results of detection by EdU/PI double staining assay. **P < 0.01, ***P < 0.001, ****P < 0.0001. **F** The increased apoptosis of HCT8 cells caused by TRIM21 knockdown after 5-FU (0.625 μg/mL) or SN-38 (25 nM) treatment was rescued by TCF3 downregulation to a certain extent. *P < 0.05, **P < 0.01, ***P < 0.001.

Rescue experiments were carried out to investigate whether TRIM21 regulated DNA replication through TCF3. Figure 6A, B showed that TCF3 knockdown partially restored the downregulation of MCM2 and MCM5 induced by TRIM21 knockdown. TCF3 downregulation also recovered the inhibitory effect of TRIM21 knockdown on cell proliferation in part (Fig. 6C). Knocking down TCF3 partly reversed the reduction in the percentage of EdU-positive cells caused by decreased TRIM21 expression, as well (Fig. 6D, E). To some extent, TCF3 knockdown rescued the increase of apoptotic cells induced by TRIM21 knockdown when treating with 5-FU or SN-38 (Fig. 6F). Thus, these results demonstrated that TRIM21 could regulate DNA replication, at least in part, by modulating TCF3 expression in CRC cells.

### TRIM21 regulated tumor growth via TCF3 in vivo

**Fig. 7:**
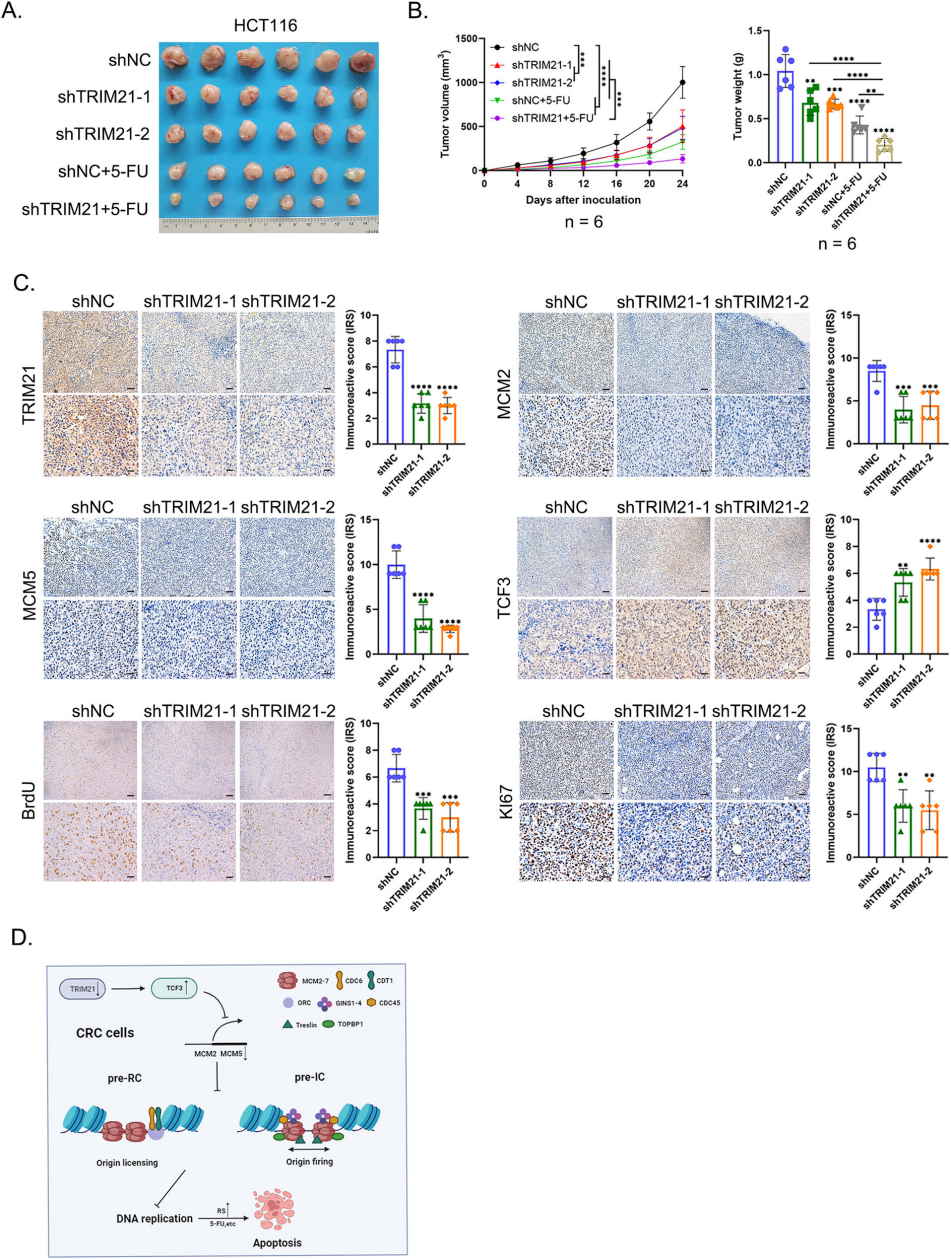
TRIM21 regulated tumor growth via TCF3 in vivo. **A** Representative images of tumors from nude mice (*n* = 6) inoculation with lentivirus stably transfected HCT116 cells with or without 5-FU intervention. **B** Analysis performed on the volume and weight of the tumors from Fig. 7A. **P < 0.01, ***P < 0.001, ****P < 0.0001. **C** Representative IHC staining images and corresponding immunoreactive scores of TRIM21, MCM2, MCM5, TCF3, BrdU and MKI67 of the tumors from Fig. 7A. Scale bars = 50 μm (upper panel) and 20 μm (lower panel). **P < 0.01, ***P < 0.001, ****P < 0.0001. **D** Schematic diagram of TRIM21 regulating DNA replication in CRC cells.

A subcutaneous xenograft tumor model was established using lentivirus-stably transfected HCT116 cell lines (Supplementary Fig. 7A, B). TRIM21 knockdown inhibited tumor growth in vivo. Tumors arising from HCT116 cells with TRIM21 knockdown had significant slower tumor growth than those without TRIM21 knockdown, and treatment with 5-FU further restrained tumor growth (Fig. 7A, B). IHC staining of tumor tissues acquired on the day of sacrifice showed decreased MCM2, MCM5, BrdU and MKI67 levels in TRIM21 knockdown groups, contrasting with elevated TCF3 levels (Fig. 7C). Besides, TCF3 knockdown partially restored tumor growth restriction caused by TRIM21 downregulation in vivo, and the corresponding results of IHC staining were also obtained (Supplementary Fig. 7C–E). Taken together, TRIM21 knockdown restrained CRC xenografts growth in vivo, at least in part, through upregulating TCF3 expression.

Altogether, TRIM21 knockdown in CRC cells upregulated TCF3 expression, which in turn inhibited *MCM2* and *MCM5* transcription, thereby hindering DNA replication. Meanwhile, RS in CRC cells will rise sharply if replication inhibitors are applied, leading to replication machinery overload and apoptosis (Fig. 7D).

## Discussion

The present study demonstrated that TRIM21 modulated *MCM2* and *MCM5* transcription by regulating TCF3 expression, thus affecting DNA replication as well as proliferation of CRC cells. In addition, TRIM21 knockdown improved the chemosensitivity of CRC cells to replication inhibitors.

It is reported that TRIM21 plays diverse roles in different cancers through its E3 ligase activity. For example, on the one hand, TRIM21 translocated β-catenin from cytoplasm to nucleus, thus promoting glioblastoma progression [46]. TRIM21 could also suppress antioxidant response of hepatocellular carcinoma cells by mediating P62 expression [47]. In CRC, TRIM21 could enhance Cisplatin resistance of tumor cells by down-regulating PAR4 and promote tumor growth in nude mice [48, 49]. On the other hand, TRIM21 could inhibit glycolysis of renal cancer cells by degrading HIF-1α to hinder tumor progression [50]. In non-small cell lung cancer, PD-L1 degradation by TRIM21 could improve anti-tumor effects of CD8-positive T cells [51]. In the present study, we found that TRIM21 positively regulated CRC cell proliferation and DNA replication through TCF3/MCM2/5 axis, and the regulation of TCF3 by TRIM21 was E3 ligase-independent, as we performed Co-IP experiment but found no interaction between TRIM21 and TCF3 proteins. Further study revealed that overexpression of an E3 ligase-dead mutant TRIM21 also induced MCM2, MCM5 upregulation as well as TCF3 downregulation, which meant that the E3 ligase function of TRIM21 was probably not engaged in it. Additionally, no direct interaction was detected between TRIM21 and the *TCF3* promoter region by ChIP assay. Collectively, these results implied an indirect regulatory relationship between TRIM21 and TCF3, a premise requiring future investigation.

Regarding research on TCF3, it has been reported to play a suppressive role in CRC tumorigenesis, but the transcriptional regulation of *MCM2* and *MCM5* by TCF3 has not been reported yet. In the field of CRC research, studies found that after TCF3 knockdown, β-catenin would translocate to nuclei and activate Wnt/β-catenin pathway to promote CRC occurrence and development. TCF3 was also found to inhibit MYC expression by binding to the Wnt-responsive DNA elements (WREs). When TCF3 expression decreased, the WREs bound more readily to the TCF4/β-catenin complex, then activated MYC, and allowed dormant CRC cells to re-enter the cell cycle [52, 53]. Our study demonstrated that TCF3 suppressed CRC development by modulating DNA replication through repressing *MCM2* and *MCM5* transcription, providing a novel mechanistic insight into its tumor-suppressive function.

What’s more, studies have revealed that aberrant MCMs expression can induce RS and enhance the efficacy of replication inhibitors. For instance, MCMs knockdown in pancreatic ductal adenocarcinoma and CRC cells enhanced the sensitivity of tumor cells to CMG inhibitors [25]. MCMs knockdown combined with DNA replication inhibitors resulted in DNA fragmentation, followed by replication fork stall and collapse [21, 22]. In our present study, TRIM21 knockdown decreased MCM2 and MCM5 expression and enhanced the inhibitory effect of 5-FU and SN-38 on CRC cells, which has not yet been reported. Recently, Zhang et al. demonstrated that Claspin ubiquitination induced by TRIM21 overexpression in the presence of RS reduced CHK1 (Ser345) phosphorylation, leading to replication fork instability and tumor formation [48]. In our study, we found that CHK1 (Ser345) phosphorylation level maintained stable after TRIM21 knockdown, and we postulated that the difference may be due to the different cell lines or drug concentration used.

In conclusion, by employing clinical samples and a series in vitro and in vivo experiments, we preliminary explored the mechanism by which TRIM21 regulating DNA replication through TCF3/MCM2/5 axis, and eventually affecting CRC development and chemosensitivity to replication inhibitors.

## Materials and methods

### Cell culture and treatment

Human normal colonic epithelial cells NCM460 and HCT8-5FU cells were purchased from iCell Bioscience Inc. (Shanghai, China). Other CRC cell lines were from the ATCC. All cells were cultured according to the ATCC protocol. All human cell lines have been authenticated using STR profiling. All experiments were performed with mycoplasma-free cells. jetPRIME^®^ DNA/siRNA transfection reagent (Polyplus, France) was used for transfection. siRNAs were purchased from GenePharma Co., Ltd (Shanghai, China), and overexpression plasmids were constructed by Generay Biotech Co., Ltd (Shanghai, China). Luciferase reporter gene plasmids were constructed by OBiO Technology Co., Ltd (Shanghai, China). Lentiviruses targeting specific genes were produced by Genomeditech Co., Ltd (Shanghai, China). UCN-01 and Caffeine were purchased from Chemegen Co., Ltd (Shanghai, China). The sequences of siRNA and shRNA were listed in Supplementary Table 2.

### RT-qPCR

Total RNA was isolated according to the protocol of TRIzol reagent (Ambion, USA). PrimeScript™ RT reagent Kit with gDNA Eraser (TAKARA, Japan) and ProFlex PCR System (Thermo Fisher Scientific, USA) were used for reverse transcription. RT-qPCR was performed with TB Green^®^ Premix Ex Taq™ II Kit (TAKARA, Japan) and StepOnePlus™ real-time PCR system (Thermo Fisher Scientific, USA). GAPDH served as an internal control. 2^−ΔΔCt^ method was utilized to calculate the relative expression of mRNA. Primer sequences were shown in Supplementary Table 3.

### Western blot

Cells were lysed with RIPA lysis buffer (Thermo Fisher Scientific, USA) and kept on ice for 1 h. Next, the supernatant was acquired after centrifugation and quantified. 40 μg total protein was then separated by SDS-PAGE and transferred onto PVDF membranes (Millipore, USA). The membranes were treated with 5% skim milk, followed by incubation with primary and secondary antibodies. Primary antibodies against GAPDH (60004-1-Ig), Histone-H3 (17168-1-AP), Lamin B1 (12987-1-AP), TRIM21 (12108-1-AP), PCNA (60097-1-Ig) and TCF3 (21242-1-AP) were from Proteintech (USA); CCND1 (#2922), CDK4 (#12790) and CDK6 (#13331) were from CST (USA); γH2AX (ab81299), pCHK1 (ab58567) and Myc-tag (ab9132) were from Abcam (UK); MCM2 (A23477), MCM5 (A5008) and Flag-tag (AE092) were from ABclonal (China). The results were visualized and imaged by the ECL kit and chemiluminescence imaging system (Tanon, China).

### Nuclear and chromatin protein extraction

Nuclear and chromatin proteins were extracted with the nuclear protein extraction kit (Thermo Fisher Scientific, USA). Briefly, cells were incubated with Cytoplasmic Extraction Reagent Ⅰ and Ⅱ sequentially on ice. The supernatant was removed and the pellet was suspended with Nuclear Extraction Reagent, followed by vortexing for 15 s every 2 min in 40 min. Lamin B1 was used as an internal control.

Chromatin protein extraction was performed with ChromaFlash™ Chromatin Extraction Kit (Epigentek, USA). In brief, cells were cross-linked and lysed with Working Lysis Buffer. After centrifugation, the supernatant was supplemented with Chromatin Buffer. Histone-H3 was used as an internal control.

### Flow cytometry analysis

Cell cycle and apoptosis analysis were carried out with PI/RNase Staining Buffer and Annexin-V-PE/7-AAD Apoptosis Detection Kit (BD Biosciences, USA), respectively. For cell cycle analysis, cells were treated with RNase and then stained with PI. For apoptosis analysis, cells were stained successively with Annexin-V-PE and 7-AAD. All samples were protected from light and tested as soon as possible.

### Cell viability and proliferation assay

Cells were seeded into 96-well plates, and proliferation/viability was detected by CCK-8 according to the protocol (DOJINDO LABORATORIES, Japan). The absorbance value at 450 nm wavelength was measured using a microplate reader (BioTek, USA).

### EdU staining and detection

EdU staining was performed by Cell-Light EdU Apollo In Vitro Kit (Ribobio, China) according to manufacturer’s instructions. Briefly, cells were pulsed with 10 μM EdU for 2 hours before fixation. After neutralization and permeabilization, cells were incubated in the dark for 10 minutes with Apollo staining solution. EdU detection was implemented with laser confocal microscopy (Leica, Germany) and flow cytometry (Beckman Coulter, USA).

### Cell synchronization

The experiment referred to several literatures with some modifications [35, 54]. Generally, cells were first treated with 2 mM thymidine for 14 h. Then, the medium was replaced with complete medium without thymidine. After 10 h release, the cells were treated with 2 mM thymidine again for another 14 h. Finally, release the cells with complete medium and collect cells at indicated time points for analysis.

### DNA fiber assay

The experiment referred to several literatures with some modifications [55–57]. Cells were incubated successively with 25 μM IdU and 250 μM CldU followed by digestion, then 2 μL cell suspension was aspirated to slide and mixed with 7 μL lysis droplet. Subsequently, DNA fibers were stretched, fixed, denatured, blocked and incubated with primary and secondary antibodies. Primary antibodies against ldU (347580, 1:25) and CldU (ab6326, 1:100) were from BD Biosciences (USA) and Abcam (UK), respectively. Donkey anti-rat Alexa Fluor 488 (A-21208, 1:100) and sheep anti-mouse Alexa Fluor 633 (A-21052, 1:100) antibodies were from Thermo Fisher Scientific (USA). Finally, DNA fibers were visualized by laser confocal microscopy (Leica, German) and measured using LAS X Life Science Microscope Software (https://www.leica-microsystems.com/).

### Immunofluorescence

Cells were seeded in confocal culture dishes, fixed, then permeabilized and blocked, followed by incubation with primary antibody against TRIM21 (12108-1-AP, Proteintech, USA) at 4 °C overnight. 12 hours later, cells were incubated with secondary antibody and anti-fluorescence quench agent (Thermo Fisher Scientific, USA). Observations were performed using laser confocal microscopy (Leica, German) as soon as possible.

### Isolation of proteins on nascent DNA (iPOND)

The experiment referred to literatures with some modifications [58, 59]. Cells were incubated with 10 μM EdU and 10 μM thymidine successively. After cross-linking and neutralization, each sample was permeabilized and treated with click reaction mixture. Then, cell precipitate was lysed, sonicated, centrifuged and streptavidin beads were aspirated into supernatant and incubated for 16–20 h with rotation. Finally, samples were eluted with 2× SDS Laemmli sample buffer and subsequently analyzed by the Western blot.

### Chromatin Immunoprecipitation (ChIP)

ChIP assay was carried out with EZ-ChIP™ Kit (Merck, Germany) according to manufacturer’s instructions. After cross-linking and neutralization, cells were resuspended in the SDS lysate mixture. Immunoprecipitation dilution buffer was added to each sample, and the mixture was sonicated and centrifuged. Next, the samples were added with indicated antibodies and incubated overnight at 4 °C. Due to the commercial unavailability of ChIP-grade antibodies against endogenous TRIM21 and TCF3, we used validated anti-Flag (AE092, ABclonal, China) and anti-Myc (ab9132, Abcam, UK) antibodies. The second day, each sample was added with protein G agarose and incubated for 1 h. After that, the agarose was washed and eluted. The eluted samples were then reversely cross-linked and used for DNA purification, purified DNA was used for PCR or qPCR analysis. The primer sequences were listed in Supplementary Table 4.

### Luciferase reporter assay

Luciferase reporter assay was conducted with Dual Luciferase Reporter Gene Assay Kit (11402ES, YEASEN, China) according to manufacturer’s protocol. Cells were co-transfected with luciferase reporter plasmids and gene overexpression plasmid or control plasmid. After 48 h, cells were lysed, and both firefly and Renilla luciferase activities were tested using a multimode microplate reader (BioTek, USA). Transfection efficiency was normalized by dividing the firefly luciferase activity by the corresponding Renilla luciferase activity.

### Immunohistochemistry (IHC)

Paraffin sections were placed at 65°C overnight and deparaffinized with xylene and ethanol. Endogenous peroxidase was then removed by 3% hydrogen peroxide-methanol. Subsequently, the sections were treated with Tris/EDTA and blocked with 1% BSA, followed by incubation with primary antibody at 4 °C overnight. Primary antibodies against TRIM21 (12108-1-AP) and TCF3 (67140-1-Ig) were from Proteintech (USA); MCM2 (A23477), MCM5 (A5008) and MKI67 (A20018) were from ABclonal (China); BrdU (ab6326) was from Abcam (UK). The following day, the sections were incubated with secondary antibody and stained with 3,3’-diaminobenzidine and hematoxylin. Eventually, these sections were dehydrated and sealed.

The results were interpreted by immunoreactive score (IRS) performed by two pathologists. The scoring criteria involved staining intensity (SI) and percentage of positive cells (PP). The SI was scored from 0 to 3 (0, negative staining; 1, weak staining; 2, moderate staining; 3, strong staining). The PP was scored from 0 to 4 (0, negative; 1, PP ≤ 25%; 2, 25% < PP ≤ 50%; 3, 50% < PP ≤ 75%; 4, 75% < PP ≤ 100%). The IRS was the product of these two scores.

### Co-Immunoprecipitation (Co-IP)

Cells were lysed with Pierce® IP Lysis Buffer (Thermo Fisher Scientific, USA) and centrifuged. The supernatant was then divided and added with anti-TRIM21 (12108-1-AP, Proteintech, USA), anti-TCF3 (21242-1-AP, Proteintech, USA) or rabbit control IgG (AC005, ABclonal, China) antibody, followed by incubation overnight at 4 °C. The second day, each sample was added with Protein A/G-Agarose (Santa Cruz Biotechnology, USA) and incubated, followed by thorough washing and elution with loading buffer.

### Patient specimens

Cohort1 contained 98 CRC samples and 97 normal samples. Clinicopathological characteristics and follow-up data were obtained from 93 CRC patients in Cohort2 (Supplementary Table 5), which included 87 pairs of paired adjacent specimens. All individuals received no preoperative chemotherapy or radiotherapy before surgery. After sample collection, immediately freeze them in liquid nitrogen for subsequent RNA extraction and qPCR experiments. Alternatively, fix the samples in formalin immediately, followed by paraffin embedding for IHC staining.

### CRC xenograft model

4-week-old BALB/c male nude mice were purchased from Charles River Laboratories (Beijing, China) and housed in a specific pathogen-free environment. After skin disinfection, we injected 2 × 10^6^ HCT116 cells into the right flanks of mice (for each group, *n* = 6) subcutaneously. When the tumor was palpable, tumor volume was measured and calculated as length × width^2^ × 0.5 every 4 days. 5-FU (10 mg/kg) was injected intraperitoneally every 2 days after tumor formation. After 24 days, mice were sacrificed by cervical dislocation after anesthesia, and the subcutaneous tumor was dissected, measured and used for IHC staining. For tissue BrdU staining, intraperitoneally inject 200 μL of 10 mg/mL BrdU (A13369, Adooq Bioscience, USA) solution 4 h prior to mouse sacrifice.

### RNA sequencing

The RNA sequencing was performed by OE Biotech Co, Ltd (Shanghai, China) using Illumina Hiseq X Ten platform to generate raw data. Each group had three biological replicates. The analysis of RNA-seq data was performed according to the TopHat-HTSeq-DeSeq2 frame.

### Statistical analysis

Statistical analysis was performed by GraphPad Prism 8.0 (GraphPad Software). All experiments were repeated at least three times, and data were presented as mean ± SD. Student’s *t*-test was used to compare the measurement data of normal distribution and comparable variation between two groups. When the data were skewed, the non-parametric Mann–Whitney *U*-test or Wilcoxon rank-sum test were used. Chi-square test was used to analyze the clinicopathological data. Survival analysis was performed with Kaplan–Meier method and log-rank test. A two-sided *P*-value < 0.05 was considered statistically significant.

## Supplementary information

Revised Supplementary Data

Full and Uncropped Western Blots

## Data Availability

Our high-throughput sequencing data can be downloaded from the GEO database under the accession number GSE276995. Other sequencing data can be downloaded from GSE100179. Other materials are available from the corresponding author on reasonable request.
