# Supplementary material for: TRIM21 promotes colorectal cancer development through regulating DNA replication by TCF3/MCM2/5 axis
Source: Cell Death Discov. 2025 Sep 25;11:422. doi: 10.1038/s41420-025-02722-3 (PMC12462522; doi:10.1038/s41420-025-02722-3)
Supplement: Supplementary file 1 — Revised Supplementary Data [file 41420_2025_2722_MOESM1_ESM.docx]

**Supplementary Data**

**Supplementary Figure 1. Detection of TRIM21 mRNA and protein levels in a normal colon epithelial cell line and some CRC cell lines**

**
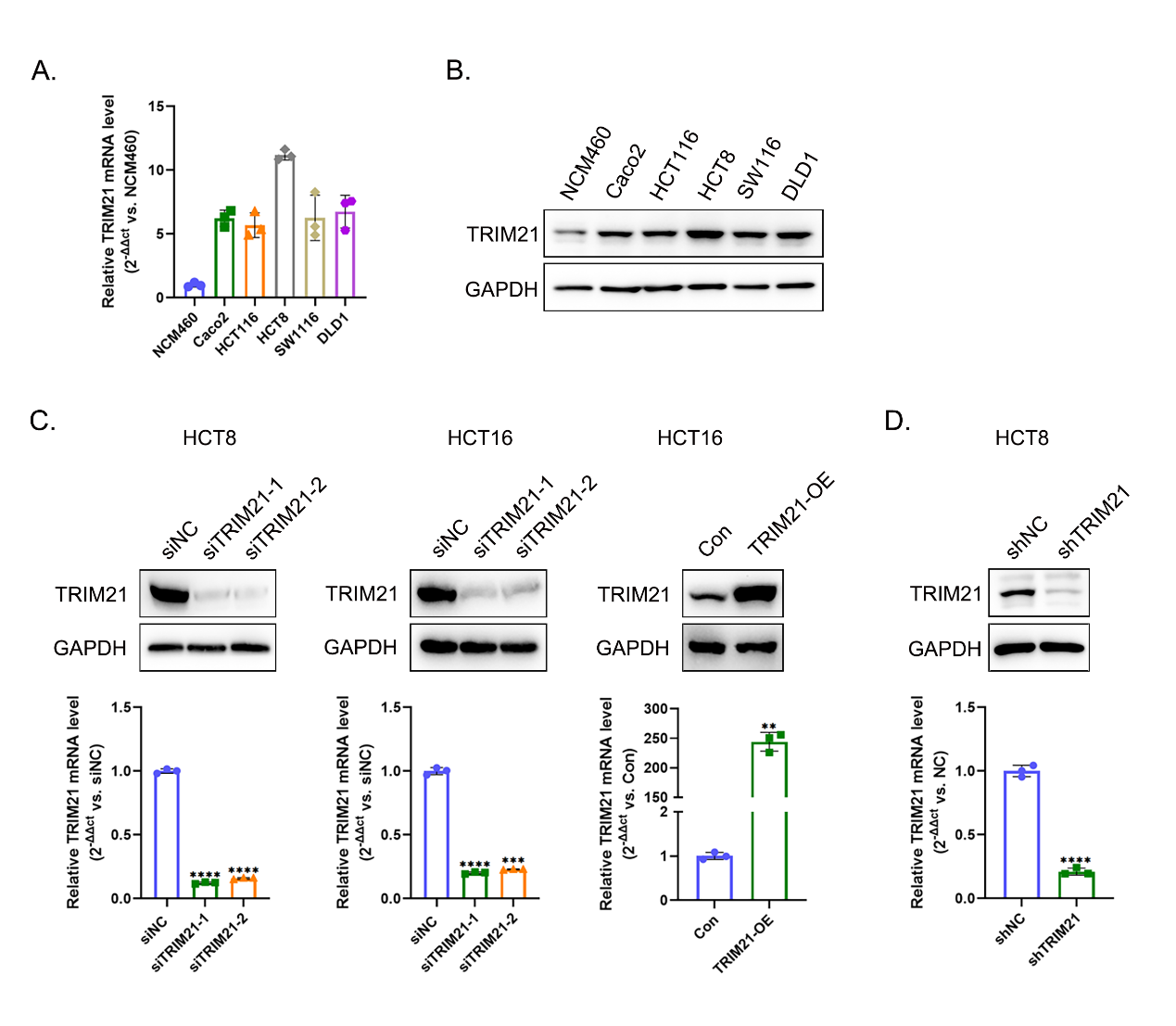
**

**A-B.** Detection of TRIM21 mRNA **(A)** and protein **(B)** levels in a normal colon epithelial cell line NCM460 and several CRC cell lines by RT-qPCR and western blot, respectively. GAPDH was used as a loading control. **C.** Validation of TRIM21 knockdown efficiency with two siRNA (siTRIM21-1 and siTRIM21-2) transfection in HCT8 and HCT116 cells by western blot and RT-qPCR (right two panels). Validation of TRIM21 overexpression efficiency with plasmid transfection in HCT116 cells by western blot and RT-qPCR (left panel). GAPDH was used as a loading control. ***P* < 0.01, ****P* < 0.001, *****P* < 0.0001. **D.** Detection of TRIM21 protein and mRNA levels in lentivirus stably transfected HCT8 cells (shNC and shTRIM21) used for RNA sequencing. GAPDH was used as a loading control. *****P* < 0.0001.

**Supplementary Figure 2. Diagrams of double thymidine block and DNA fiber assay**

**
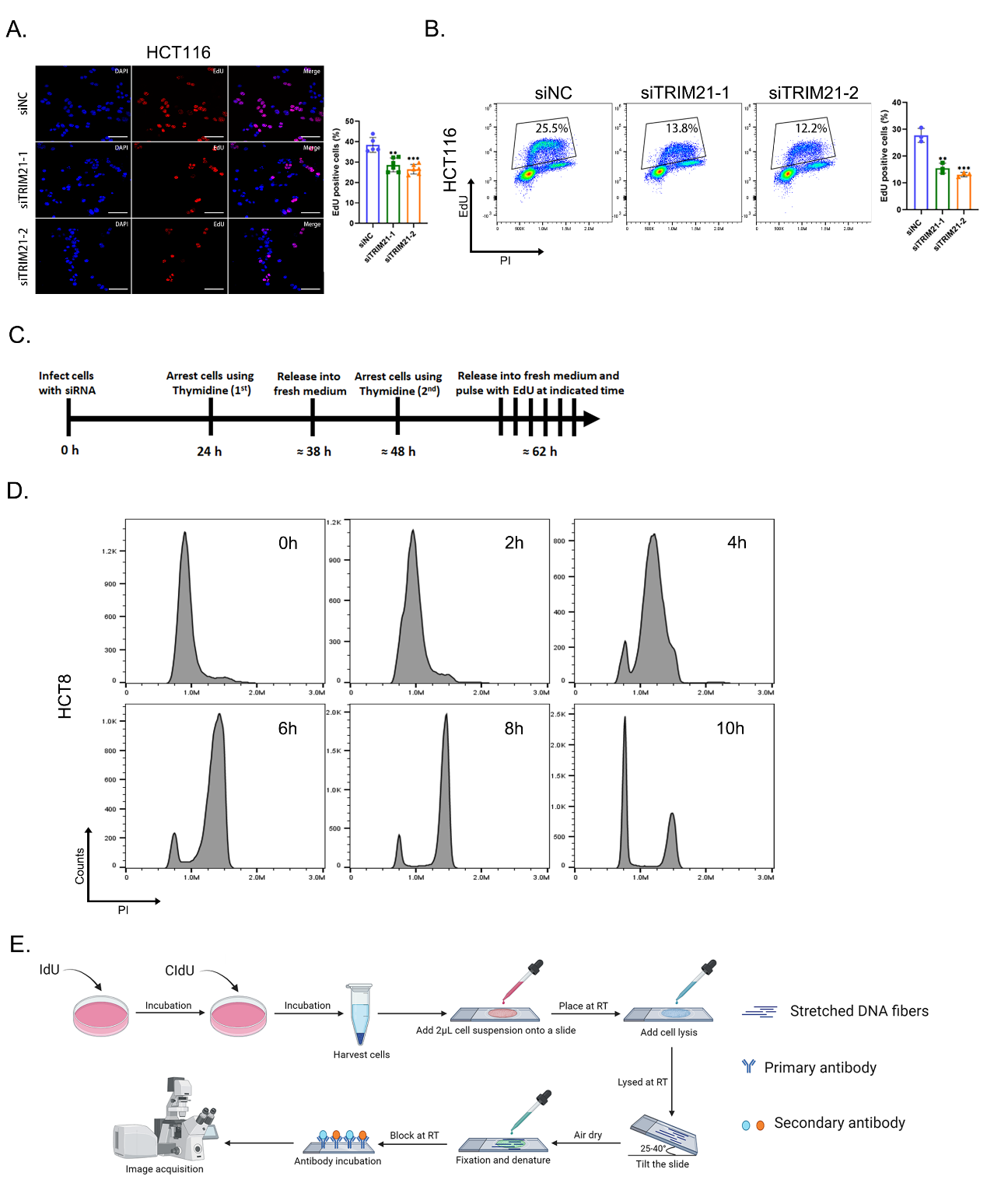
**

**A.** Fluorescence detection of EdU-positive cells in HCT116 cells with TRIM21 knockdown. Nuclei were stained with DAPI (blue) and replicating DNA was incorporated with EdU (red). Scale bars = 50 μm. ***P* < 0.01, ****P* < 0.001. **B.** Detection of EdU-positive cells in HCT116 cells with TRIM21 knockdown by EdU/PI double staining assay. ***P* < 0.01, ****P* < 0.001. **C.** Fishbone diagram of cell cycle synchronization at G1/S boundary achieved by double thymidine block. **D.** Cell cycle status of HCT8 cells at different time points (0h, 2h, 4h, 6h, 8h, 10h) after synchronization release. Most cells were synchronized at G1/S boundary at the time of release (0h). At 2^nd^ to 6^th^ hour (2h to 6h) after release, cells began to enter and concentrated in S phase. At 8^th^ hour (8h), most cells entered the G2/M phase and progressed to the G1 phase by the 10^th^ hour (10h). **E.** General flow diagram of DNA fiber assay. ldU and CldU refer to two halogenated nucleotides that successively label nascent DNA. RT, room temperature.

**Supplementary Figure 3. Temporal and spatial distribution of TRIM21 protein**

**
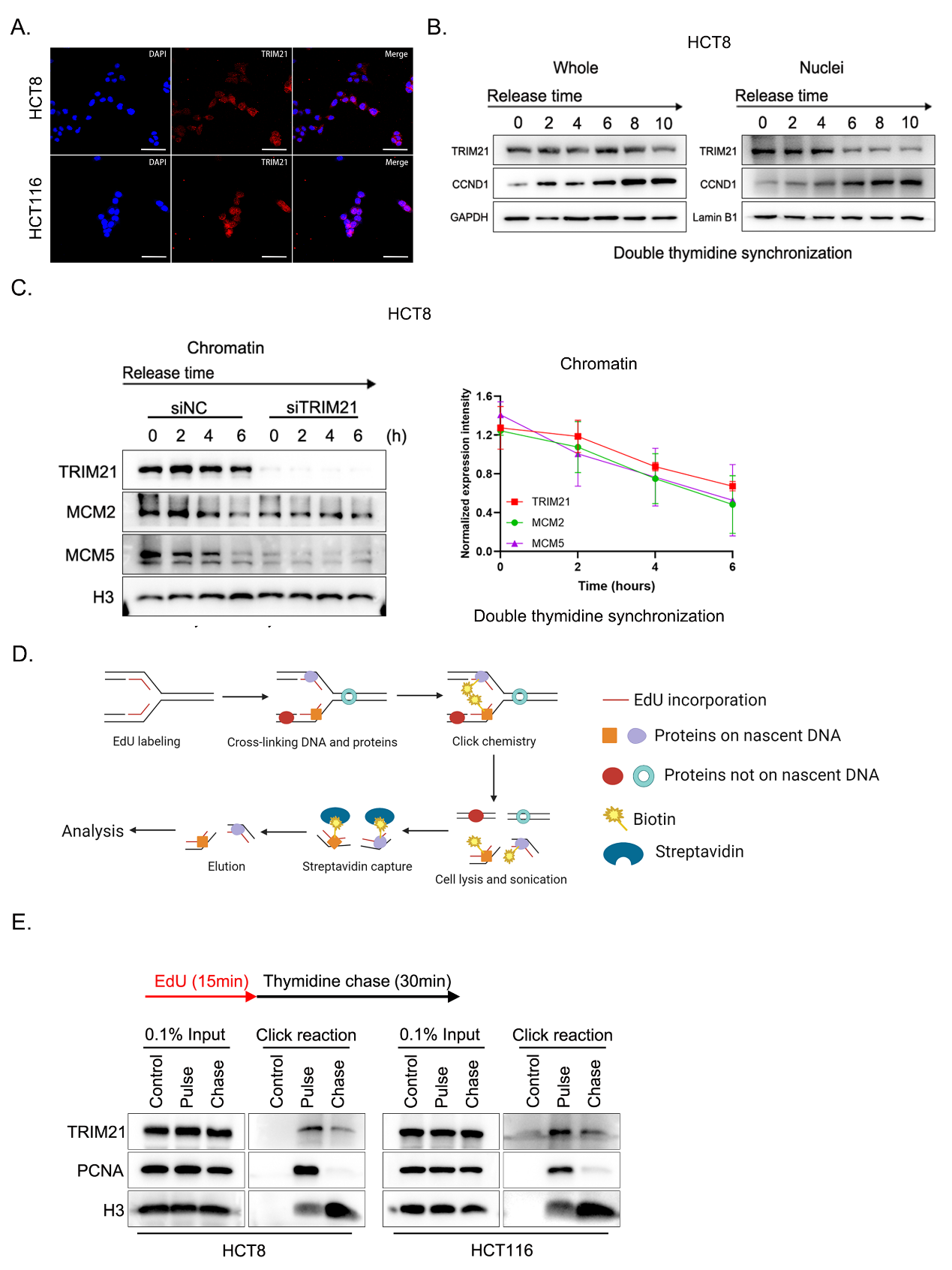
**

**A.** Assessment of TRIM21 protein (red) localization in CRC cells by immunofluorescence. Nuclei were stained with DAPI (blue). Scale bars = 50 μm. **B.** Detection of TRIM21 and CCND1 protein levels at different time points (0h, 2h, 4h, 6h, 8h, 10h) after synchronization release in whole cell lysates and nuclei fractions of HCT8 cells. GAPDH and Lamin B1 were used as loading controls for total and nuclei protein, respectively. **C.** Detection of TRIM21, MCM2 and MCM5 protein levels at different time points (0h, 2h, 4h, 6h) after synchronization release in chromatin fractions of HCT8 cells from two groups (siNC and siTRIM21). Histone H3 was used as a loading control for chromatin protein. **D.** General flow diagram of iPOND assay. **E.** Detection of TRIM21 protein in CRC cells by iPOND assay. PCNA protein was enriched specifically at the replication fork. Histone H3 was a chromatin-associated protein that was enriched in both replication fork and thymidine chase samples.

**Supplementary Figure 4. Effects of TRIM21 knockdown on DNA replication and chemosensitivity of HCT8-5FU cells**


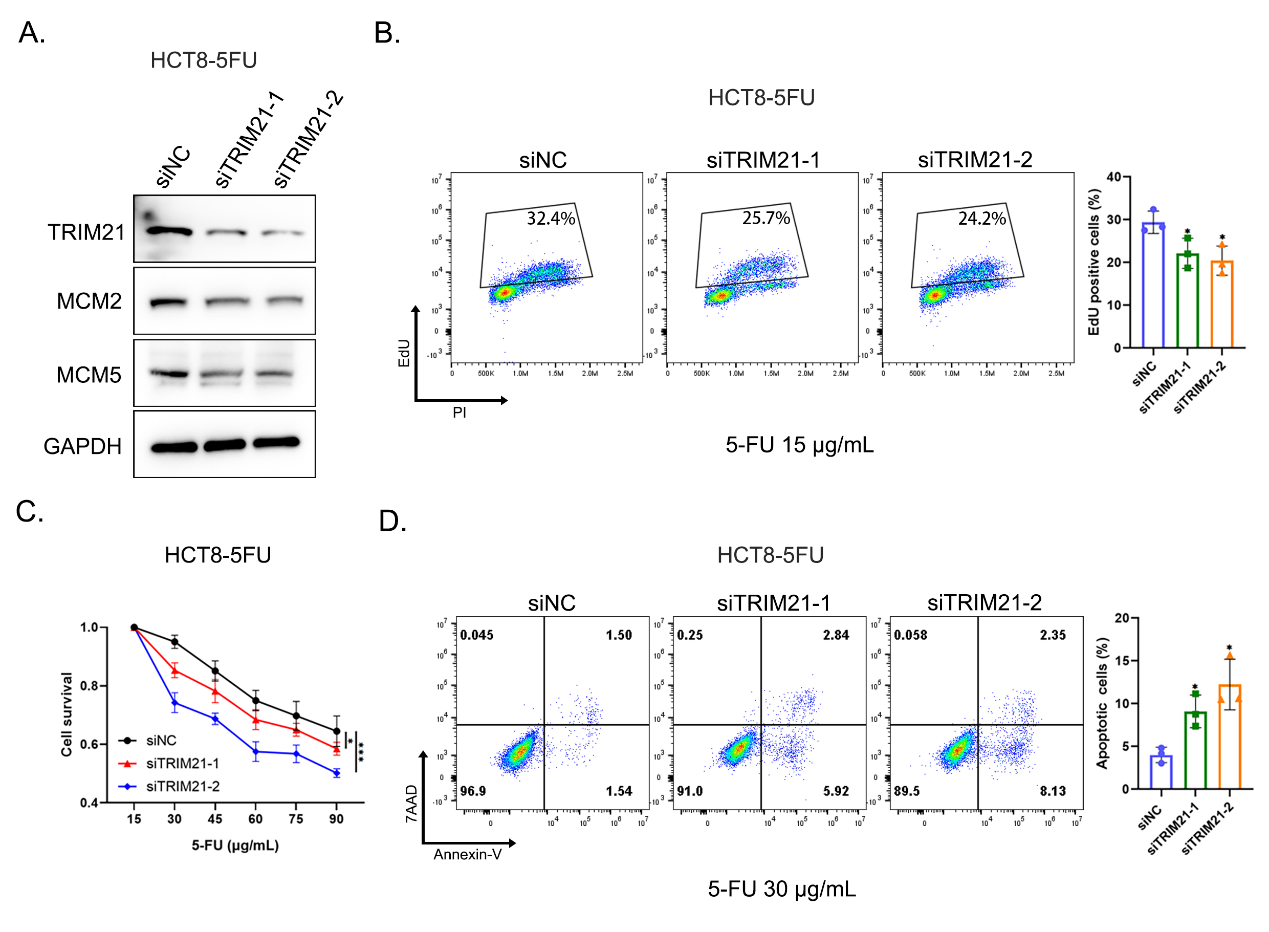


**A.** Detection of MCM2 and MCM5 protein levels after TRIM21 knockdown in HCT8-5FU cells. GAPDH was used as a loading control. **B.** Detection of EdU-positive cells in HCT8-5FU cells with or without TRIM21 knockdown by EdU/PI double staining assay. **P* < 0.05. **C.** Survival of HCT8-5FU cells with or without TRIM21 knockdown after treatment with different concentrations of 5-FU. **P* < 0.05, ****P* < 0.001. **D.** Apoptosis analysis of HCT8-5FU cells with or without TRIM21 knockdown after treatment with relatively low concentration 5-FU (30 μg/mL) by 7AAD/Annexin-V staining. **P* < 0.05.

**Supplementary Figure 5. Regulation of DNA replication by TRIM21 was independent of DNA damage response**

**
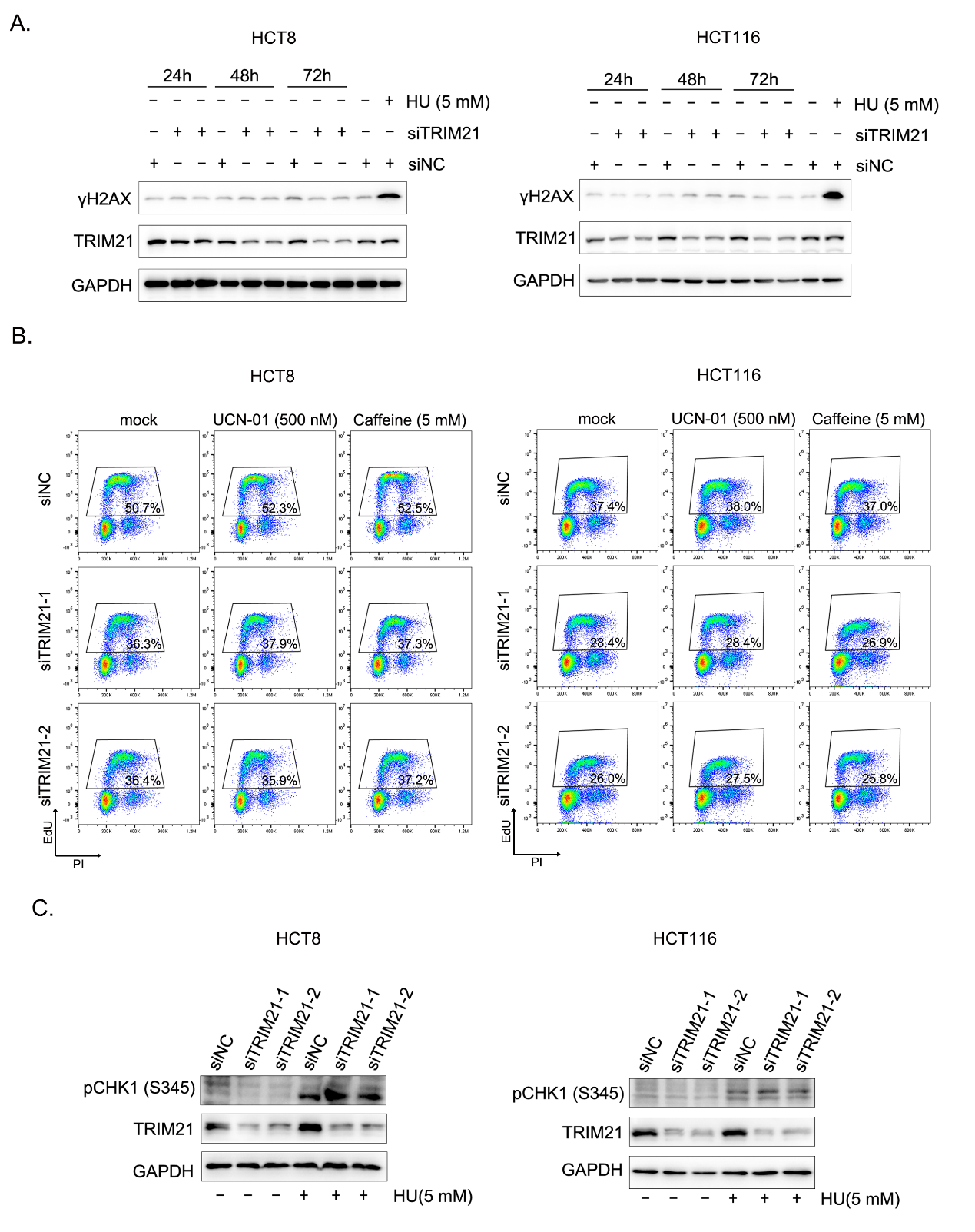
**

**A.** Detection of γH2AX level in CRC cells after HU treatment and at different time points (24h, 48h, 72h) after TRIM21 knockdown. GAPDH was used as a loading control. **B.** Detection of EdU-positive cells in CRC cells after TRIM21 knockdown with or without UCN-01/Caffeine treatment by EdU/PI double staining assay. **C.** Detection of pCHK1 (S345) level in CRC cells after TRIM21 knockdown with or without HU treatment. GAPDH was used as a loading control.

**Supplementary Figure 6. Interaction between TRIM21 and TCF3**

**
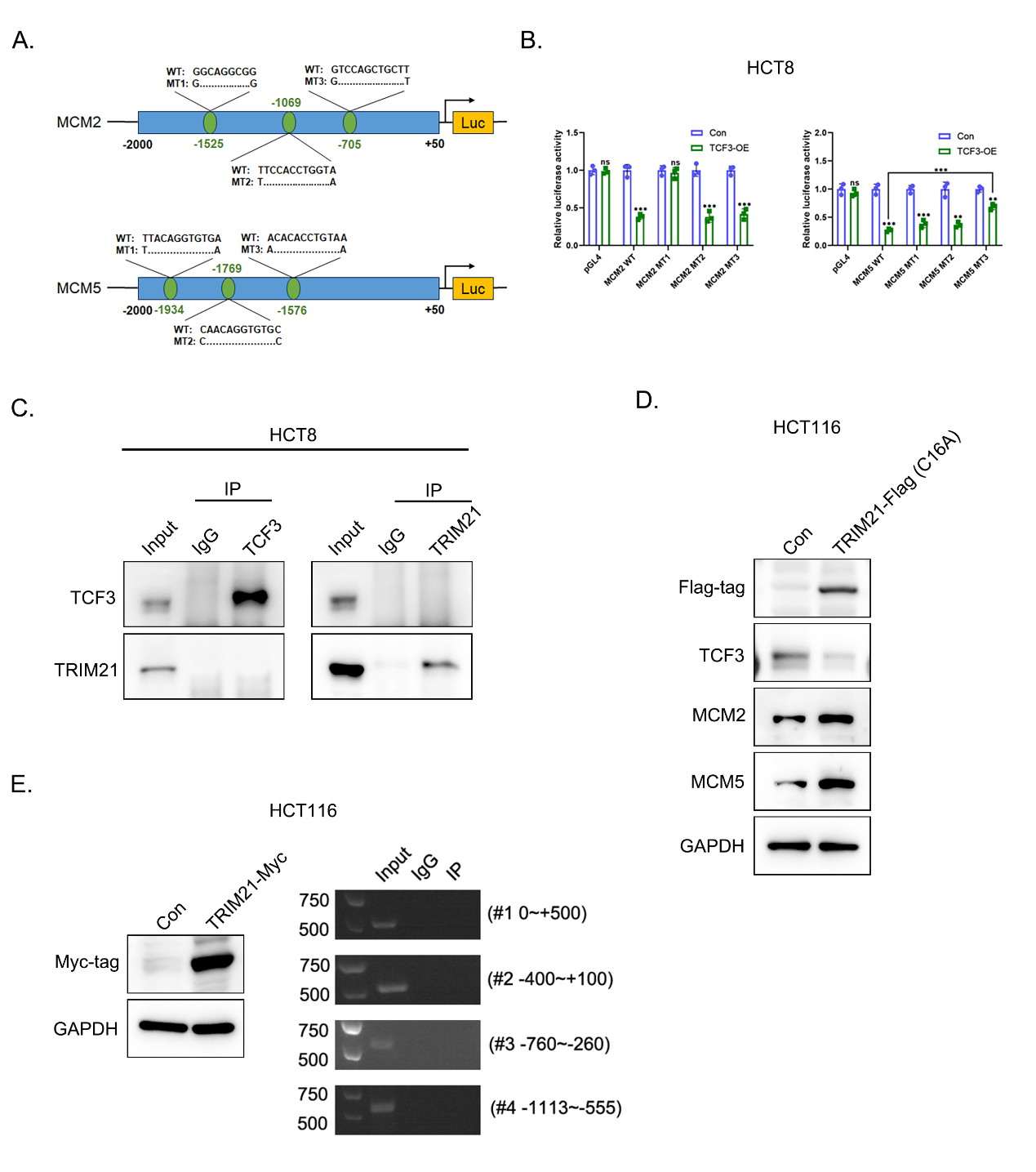
**

**A.** Schematic of luciferase reporter constructs with wild type (WT) or mutant (MT) putative TCF3-binding sites (green dots) in *MCM2* and *MCM5* promoter regions. **B.** Luciferase activities of luciferase reporter plasmids containing WT or MT *MCM2*/*MCM5* promoter in control or TCF3-overexpressing HCT8 cells. ***P* < 0.01, ****P* < 0.001, ns = no significance. **C.** Validation of the interaction between TRIM21 and TCF3 protein by Co-IP assay. IgG was used as a negative control. **D.** Detection of protein levels of MCM2, MCM5 and TCF3 after overexpressing a E3 ligase-dead mutant of TRIM21 (C16A) in HCT116 cells. GAPDH was used as a loading control. **E.** HCT116 cells transfected with Myc-tagged TRIM21 overexpression plasmid were subjected to ChIP assay to assess TRIM21 binding to *TCF3* promoter region. GAPDH was used as a loading control for western blot. IgG was used as a negative control for ChIP assay.

**Supplementary Figure 7. TRIM21 regulated tumor growth via TCF3 in vivo**


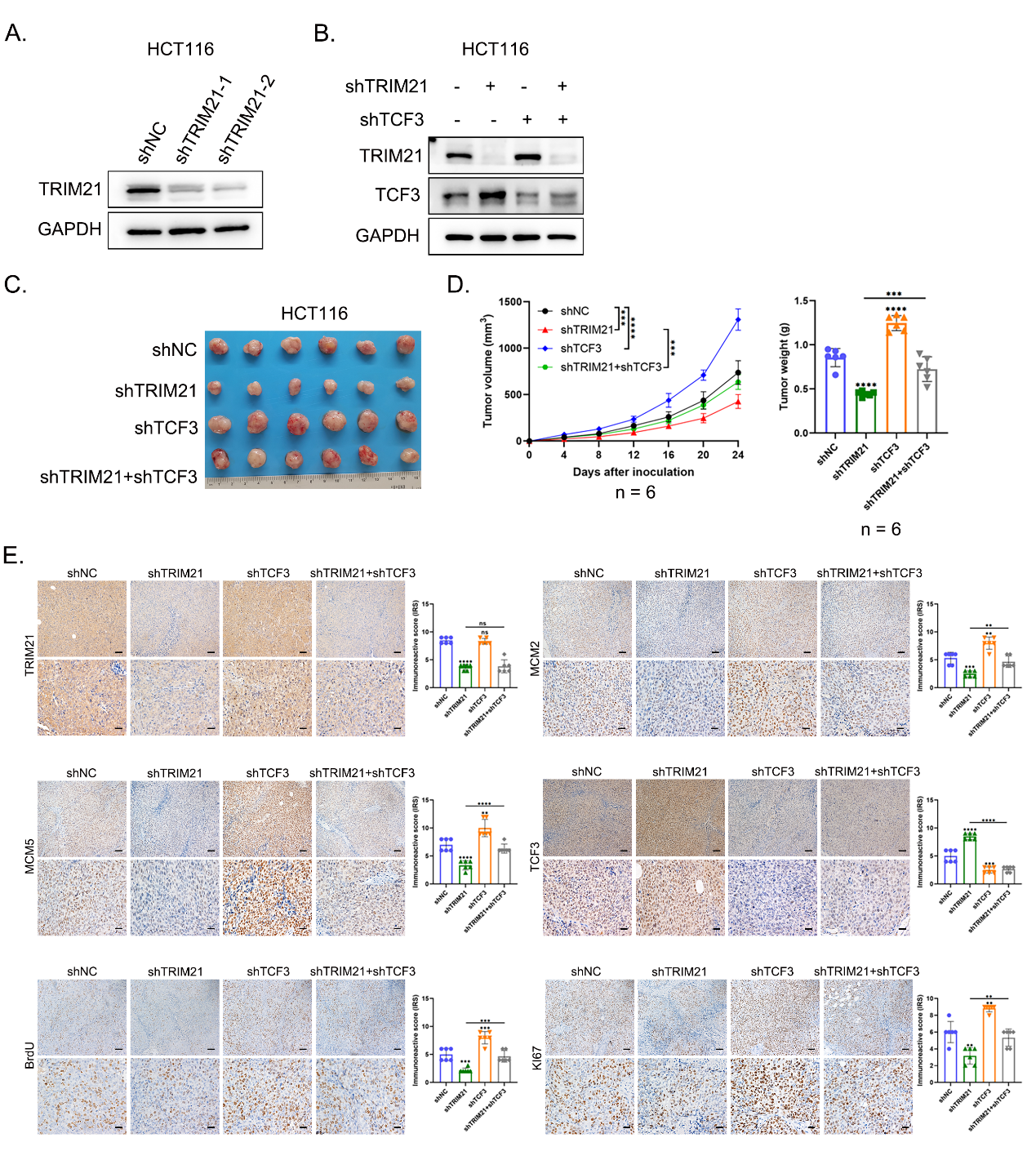


**A-B.** Detection of TRIM21 and TCF3 protein levels in lentivirus stably transfected HCT116 cells used for CRC xenograft models. GAPDH was used as a loading control. **C.** Representative images of tumors from nude mice (n = 6) inoculation with lentivirus stably transfected HCT116 cells. **D.** Analysis performed on the volume and weight of the tumors from Supplementary Figure 7C. ****P* < 0.001, *****P* < 0.0001. **E.** Representative IHC staining images and corresponding immunoreactive scores of TRIM21, MCM2, MCM5, TCF3, BrdU and Ki67 of the tumors from Supplementary Figure 7C. Scale bars = 50 μm (upper panel) and 20 μm (lower panel). ***P* < 0.01, ****P* < 0.001, *****P* < 0.0001, ns = no significance.

**Supplementary Tables**

**Supplementary Table 1. The correlation between TRIM21 level and clinicopathological features**

| Clinicopathological Feature | Total  (n = 93) | TRIM21 Expression | | P-value  (χ2 test) |
| --- | --- | --- | --- | --- |
|  |  | IRS ≤ 4  (n = 29,31.18%) | IRS > 4  (n = 64,68.82%) |  |
| Gender |  |  |  |  |
| Male | 44 (47.31) | 12 (41.38) | 32 (50.00) | 0.441 |
| Female | 49 (52.69) | 17 (58.62) | 32 (50.00) |  |
| Age (Years) |  |  |  |  |
| <65 | 37 (39.78) | 12 (41.38) | 25 (39.06) | 0.833 |
| ≥65 | 56 (60.22) | 17 (58.62) | 39 (60.94) |  |
| Tumor size (cm) |  |  |  |  |
| <5 | 37 (39.78) | 12 (41.38) | 25 (39.06) | 0.833 |
| ≥5 | 56 (60.22) | 17 (58.62) | 39 (60.94) |  |
| Tumor location |  |  |  |  |
| Left | 51 (54.84) | 15 (51.72) | 36 (56.25) | 0.685 |
| Right | 42 (45.16) | 14 (48.28) | 28 (43.75) |  |
| TNM Stage (AJCC) |  |  |  |  |
| Stage I | 5 (5.38) | 2 (6.90) | 3 (4.69) | 0.651 |
| Stage II | 53 (56.99) | 18 (62.07) | 35 (54.69) |  |
| Stage III | 35 (37.63) | 9 (31.03) | 26 (40.63) |  |
| T stage |  |  |  |  |
| T1,2 | 5 (5.38) | 2 (6.90) | 3 (4.69) | 0.662 |
| T3,4 | 88 (94.62) | 27 (93.10) | 61 (95.31) |  |
| Lymph node metastasis |  |  |  |  |
| Absent | 58 (62.37) | 20 (68.97) | 38 (59.38) | 0.377 |
| Present | 35 (37.63) | 9 (31.03) | 26 (40.63) |  |

**Supplementary Table 2. Targeted sequences of siRNA and shRNA**

| siRNA/shRNA | Sequences |
| --- | --- |
| siTRIM21-1 | GACTTCACCTGTTCTGTGA |
| siTRIM21-2 | GGACCTGGATATTACCTCT |
| siTCF3-1 | CCCGGATCACTCAAGCAATAA |
| siTCF3-2 | CAGCCTCTCTTCATCCACATT |
| shTRIM21-1 | GGCAGCGCTTTCTGCTCAAGA |
| shTRIM21-2 | GAAGAGAGATTTGATAGTTAT |
| shTCF3 | CCCGGATCACTCAAGCAATAA |

**Supplementary Table 3. Primer sequences for RT-qPCR**

| Gene | Primer (Forward) | Primer (Reverse) |
| --- | --- | --- |
| TRIM21 | CAGAACTCAGGAGTGTGTGCCA | TCCAAGCCTCACTTGTCTCCGA |
| GAPDH | GTCTCCTCTGACTTCAACAGCG | ACCACCCTGTTGCTGTAGCCAA |
| MCM2 | ATGATCGAGAGCATCGAGAACC | GCCAAGTCCTCATAGTTCACCA |
| MCM5 | GGAAGTGCAACACAGATCAGG | AGGGACGACCTTGTCACACA |
| CCND1 | GCTGCGAAGTGGAAACCATC | CCTCCTTCTGCACACATTTGAA |
| CDK4 | ATGGCTACCTCTCGATATGAGC | ATTGGGGACTCTCACACTCT |
| CDK6 | GCTGACCAGCAGTACGAATG | GCACACATCAAACAACCTGACC |
| TCF3 | CCAGACCAAACTGCTCATCCTG | TCGCCGTTTCAAACAGGCTGCT |

**Supplementary Table 4. Primer sequences for ChIP**

| Gene | Primer (Forward) | Primer (Reverse) |
| --- | --- | --- |
| MCM2 | TCACGCCTGTAATCCTAGCAC | CTCACTGCAACCTCCACCTC |
| MCM5 | TCATGCCTGTAATCCCAGCA | TCACTGCAACTTCCACCTCC |
| TCF3 #1 | ATCGTTATAAAATGTCACGTTTATTGC | CCCCATGGTAGATGCAAGGG |
| TCF3 #2 | GACAGACTCAGACTTCGAGCC | GGATGATTGGGACTTTAAAACGACC |
| TCF3 #3 | CTGAGCCTTCTCCTCCCTAAG | TAGGAACCATCCAGGACCT |
| TCF3 #4 | TAGTAGAGATGGGATTTCGCCA | CTGCTCATCCCAAAGTGCCC |

**Supplementary Table 5. Clinicopathological and follow-up information of patients in Cohort 2**

| Patients | Alive or Dead | Survival time (months) | Gender | Age (years) | Tumor size (cm) | Location | Lymphatic metastasis | T stage | AJCC stage |
| --- | --- | --- | --- | --- | --- | --- | --- | --- | --- |
| 1 | Dead | 32 | Female | 78 | 6.0 | Right | Absent | T4a | Ⅱ |
| 2 | Dead | 7 | Female | 74 | 10.0 | Left | Present | T3 | Ⅲ |
| 3 | Dead | 14 | Female | 81 | 4.5 | Right | Absent | T3 | Ⅱ |
| 4 | Alive | 97 | Female | 64 | 5.0 | Right | Absent | T4b | Ⅱ |
| 5 | Alive | 97 | Male | 62 | 7.0 | Left | Absent | T3 | Ⅱ |
| 6 | Dead | 10 | Male | 57 | 3.0 | Right | Present | T4b | Ⅲ |
| 7 | Alive | 97 | Male | 76 | 5.0 | Left | Absent | T4a | Ⅱ |
| 8 | Alive | 97 | Female | 60 | 5.0 | Left | Present | T3 | Ⅲ |
| 9 | Dead | 40 | Female | 60 | 7.0 | Right | Absent | T3 | Ⅱ |
| 10 | Alive | 97 | Male | 61 | 6.0 | Left | Present | T3 | Ⅲ |
| 11 | Dead | 9 | Female | 54 | 4.0 | Left | Absent | T3 | Ⅱ |
| 12 | Alive | 97 | Male | 74 | 5.0 | Left | Absent | T3 | Ⅱ |
| 13 | Dead | 47 | Male | 74 | 4.5 | Left | Absent | T3 | Ⅱ |
| 14 | Alive | 96 | Female | 70 | 9.0 | Right | Absent | T3 | Ⅱ |
| 15 | Dead | 91 | Female | 76 | 4.0 | Right | Absent | T3 | Ⅱ |
| 16 | Dead | 43 | Male | 57 | 6.0 | Left | Absent | T3 | Ⅱ |
| 17 | Dead | 76 | Female | 66 | 7.0 | Left | Present | T3 | Ⅲ |
| 18 | Alive | 96 | Male | 65 | 9.0 | Left | Absent | T3 | Ⅱ |
| 19 | Dead | 27 | Female | 50 | 2.5 | Left | Present | T4a | Ⅲ |
| 20 | Alive | 96 | Male | 80 | 4.5 | Right | Absent | T3 | Ⅱ |
| 21 | Dead | 2 | Female | 70 | 5.0 | Right | Present | T3 | Ⅲ |
| 22 | Alive | 95 | Female | 47 | 9.0 | Right | Present | T3 | Ⅲ |
| 23 | Alive | 95 | Male | 68 | 6.0 | Right | Absent | T3 | Ⅱ |
| 24 | Alive | 95 | Female | 72 | 4.0 | Left | Absent | T3 | Ⅱ |
| 25 | Dead | 8 | Male | 90 | 5.0 | Left | Present | T3 | Ⅲ |
| 26 | Alive | 95 | Male | 65 | 4.0 | Left | Present | T4a | Ⅲ |
| 27 | Alive | 95 | Male | 61 | 7.0 | Right | Absent | T4a | Ⅱ |
| 28 | Dead | 10 | Female | 61 | 6.0 | Left | Present | T3 | Ⅲ |
| 29 | Dead | 16 | Female | 78 | 4.0 | Left | Absent | T3 | Ⅱ |
| 30 | Dead | 39 | Male | 67 | 5.0 | Right | Absent | T3 | Ⅱ |
| 31 | Alive | 94 | Male | 68 | 3.5 | Left | Absent | T2 | Ⅰ |
| 32 | Dead | 10 | Male | 76 | 6.0 | Left | Present | T3 | Ⅲ |
| 33 | Alive | 94 | Male | 85 | 7.0 | Left | Present | T3 | Ⅲ |
| 34 | Alive | 94 | Female | 63 | 6.0 | Left | Absent | T3 | Ⅱ |
| 35 | Dead | 9 | Female | 72 | 3.0 | Right | Present | T3 | Ⅲ |
| 36 | Alive | 94 | Female | 78 | 3.5 | Left | Absent | T3 | Ⅱ |
| 37 | Alive | 94 | Male | 63 | 5.0 | Left | Absent | T3 | Ⅱ |
| 38 | Dead | 7 | Male | 77 | 5.0 | Left | Present | T4a | Ⅲ |
| 39 | Alive | 93 | Female | 58 | 8.0 | Right | Absent | T3 | Ⅱ |
| 40 | Dead | 16 | Male | 62 | 9.0 | Left | Present | T3 | Ⅲ |
| 41 | Alive | 93 | Male | 75 | 5.0 | Right | Absent | T3 | Ⅱ |
| 42 | Alive | 93 | Male | 58 | 4.0 | Right | Present | T3 | Ⅲ |
| 43 | Dead | 5 | Female | 83 | 5.0 | Right | Absent | T3 | Ⅱ |
| 44 | Alive | 92 | Male | 75 | 4.0 | Right | Absent | T4a | Ⅱ |
| 45 | Alive | 92 | Male | 51 | 5.0 | Left | Absent | T3 | Ⅱ |
| 46 | Alive | 92 | Female | 86 | 6.0 | Right | Absent | T3 | Ⅱ |
| 47 | Dead | 44 | Male | 72 | 6.0 | Right | Present | T3 | Ⅲ |
| 48 | Dead | 29 | Male | 65 | 7.0 | Right | Present | T4a | Ⅲ |
| 49 | Dead | 11 | Male | 80 | 5.0 | Left | Present | T3 | Ⅲ |
| 50 | Alive | 91 | Female | 75 | 6.0 | Left | Absent | T3 | Ⅱ |
| 51 | Dead | 31 | Female | 60 | 4.0 | Left | Absent | T3 | Ⅱ |
| 52 | Alive | 91 | Male | 78 | 4.5 | Right | Absent | T3 | Ⅱ |
| 53 | Alive | 91 | Male | 51 | 2.0 | Right | Present | T3 | Ⅲ |
| 54 | Alive | 91 | Male | 72 | 3.0 | Left | Absent | T1 | Ⅰ |
| 55 | Dead | 13 | Female | 72 | 4.0 | Right | Absent | T3 | Ⅱ |
| 56 | Dead | 12 | Male | 65 | 5.0 | Left | Absent | T3 | Ⅱ |
| 57 | Dead | 58 | Male | 79 | 2.5 | Right | Absent | T2 | Ⅰ |
| 58 | Alive | 91 | Female | 43 | 3.5 | Left | Present | T3 | Ⅲ |
| 59 | Alive | 91 | Female | 61 | 5.0 | Left | Absent | T3 | Ⅱ |
| 60 | Dead | 32 | Male | 73 | 7.0 | Right | Absent | T4a | Ⅱ |
| 61 | Dead | 89 | Male | 71 | 5.0 | Left | Present | T3 | Ⅲ |
| 62 | Dead | 65 | Female | 80 | 5.0 | Right | Present | T3 | Ⅲ |
| 63 | Dead | 13 | Male | 65 | 6.0 | Right | Present | T3 | Ⅲ |
| 64 | Dead | 16 | Female | 52 | 9.0 | Right | Present | T3 | Ⅲ |
| 65 | Alive | 90 | Female | 81 | 4.8 | Left | Absent | T2 | Ⅰ |
| 66 | Dead | 22 | Male | 60 | 5.0 | Left | Present | T3 | Ⅲ |
| 67 | Alive | 89 | Female | 69 | 2.8 | Left | Absent | T4b | Ⅱ |
| 68 | Alive | 88 | Female | 64 | 3.5 | Left | Absent | T3 | Ⅱ |
| 69 | Dead | 38 | Male | 65 | 6.0 | Left | Absent | T4b | Ⅱ |
| 70 | Dead | 18 | Male | 83 | 10.0 | Right | Present | T4a | Ⅲ |
| 71 | Alive | 88 | Female | 54 | 3.3 | Left | Absent | T2 | Ⅰ |
| 72 | Alive | 88 | Female | 79 | 5.0 | Left | Absent | T3 | Ⅱ |
| 73 | Alive | 88 | Female | 60 | 6.0 | Left | Absent | T3 | Ⅱ |
| 74 | Alive | 88 | Female | 50 | 3.0 | Left | Present | T3 | Ⅲ |
| 75 | Alive | 88 | Female | 54 | 3.0 | Right | Present | T3 | Ⅲ |
| 76 | Dead | 66 | Female | 62 | 5.0 | Left | Absent | T3 | Ⅱ |
| 77 | Alive | 88 | Male | 74 | 5.0 | Right | Present | T3 | Ⅲ |
| 78 | Dead | 36 | Female | 65 | 5.0 | Right | Present | T3 | Ⅲ |
| 79 | Alive | 88 | Male | 75 | 3.0 | Right | Present | T3 | Ⅲ |
| 80 | Alive | 88 | Male | 70 | 4.5 | Right | Present | T3 | Ⅱ |
| 81 | Alive | 88 | Female | 73 | 6.5 | Right | Absent | T3 | Ⅱ |
| 82 | Alive | 88 | Male | 58 | 6.0 | Right | Absent | T3 | Ⅱ |
| 83 | Dead | 32 | Female | 71 | 2.5 | Right | Absent | T3 | Ⅱ |
| 84 | Alive | 88 | Female | 76 | 4.0 | Left | Absent | T4a | Ⅱ |
| 85 | Alive | 87 | Female | 61 | 4.0 | Right | Absent | T4a | Ⅱ |
| 86 | Dead | 57 | Female | 75 | 7.0 | Left | Absent | T3 | Ⅱ |
| 87 | Dead | 4 | Female | 76 | 6.0 | Left | Present | T3 | Ⅲ |
| 88 | Alive | 97 | Female | 77 | 6.0 | Right | Absent | T3 | Ⅱ |
| 89 | Dead | 36 | Male | 61 | 3.5 | Left | Absent | T3 | Ⅱ |
| 90 | Dead | 25 | Male | 60 | 3.0 | Left | Absent | T3 | Ⅱ |
| 91 | Dead | 25 | Female | 64 | 6.0 | Right | Present | T4b | Ⅲ |
| 92 | Alive | 94 | Female | 55 | 3.0 | Left | Absent | T3 | Ⅱ |
| 93 | Dead | 17 | Female | 63 | 4.0 | Left | Absent | T3 | Ⅱ |
