# Supplementary material for: TRIM21 promotes colorectal cancer development through regulating DNA replication by TCF3/MCM2/5 axis
Source: Cell Death Discov. 2025 Sep 25;11:422. doi: 10.1038/s41420-025-02722-3 (PMC12462522; doi:10.1038/s41420-025-02722-3)

Figure 2D

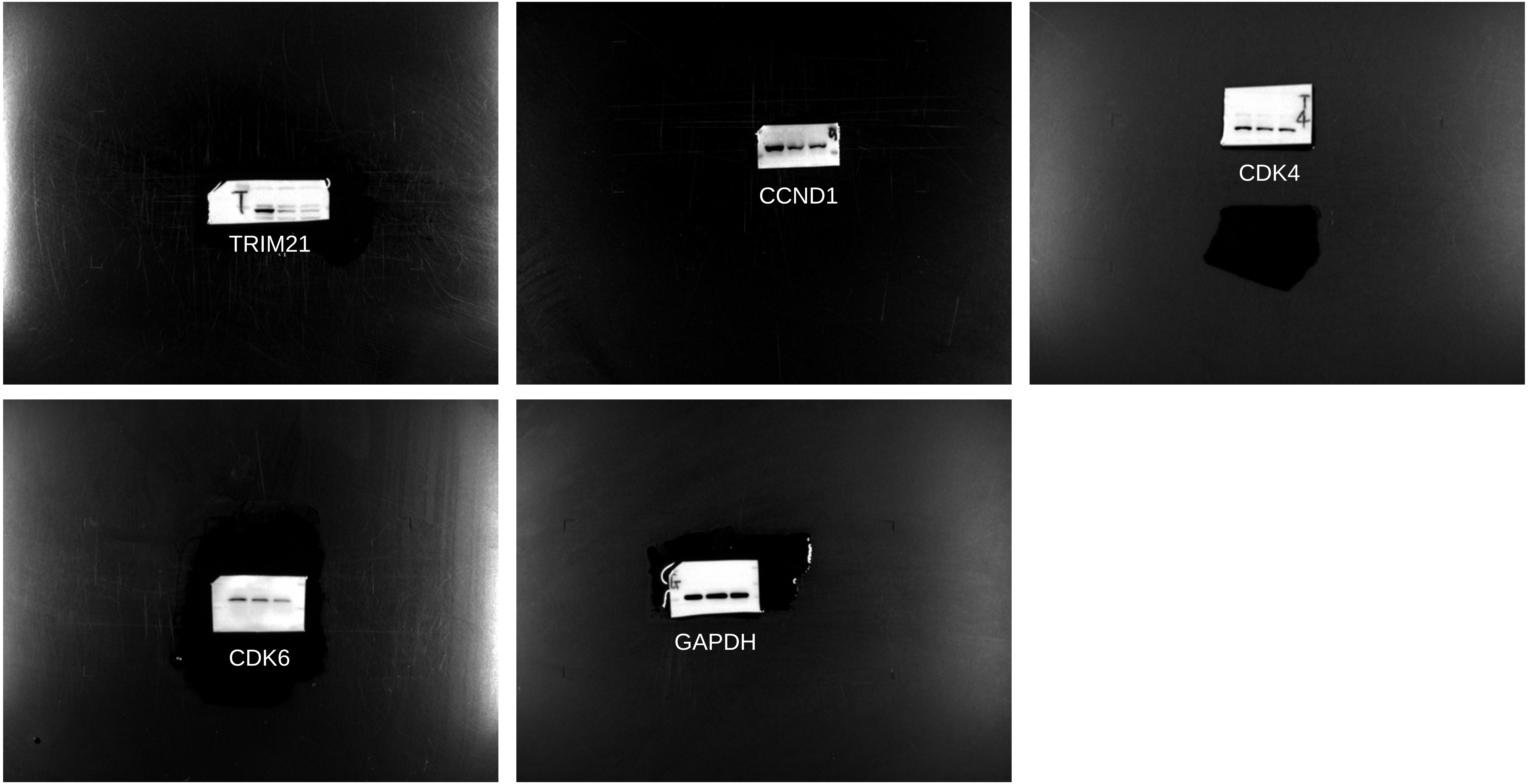

Figure 2D

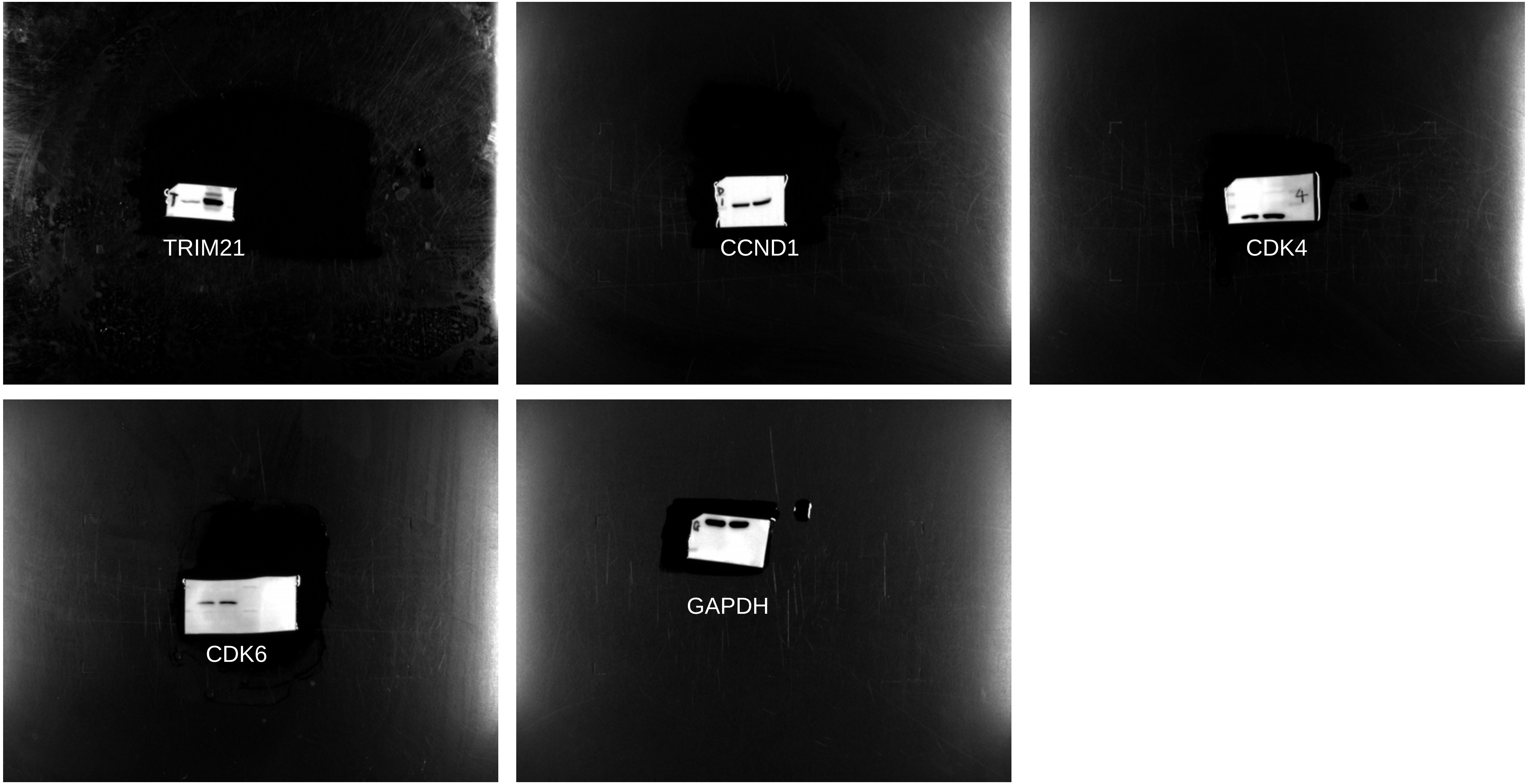

Figure 2F Whole HCT8

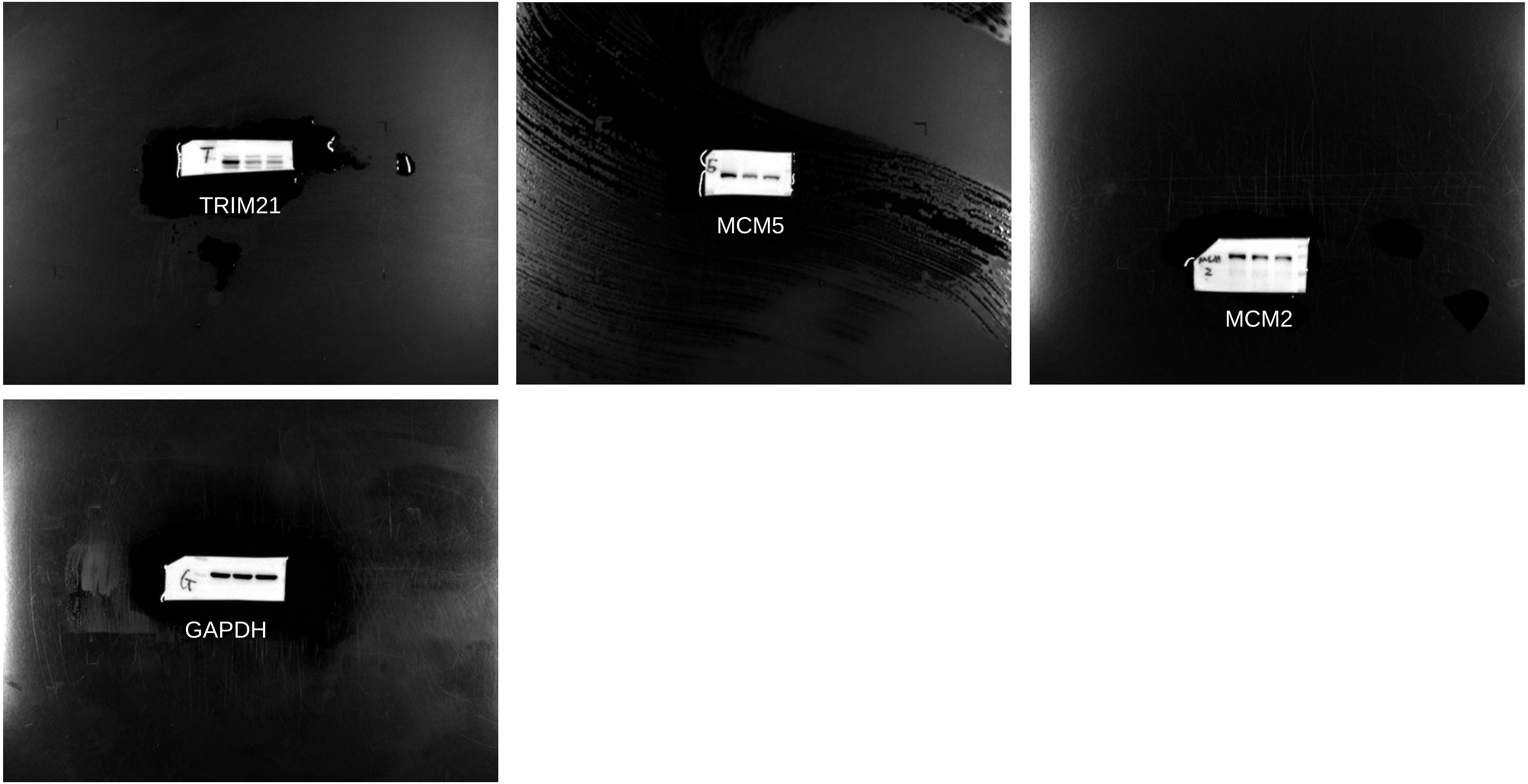

Figure 2F Nuclei HCT8

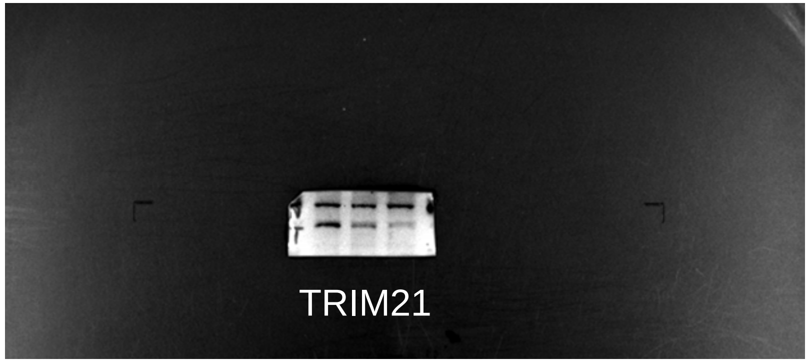

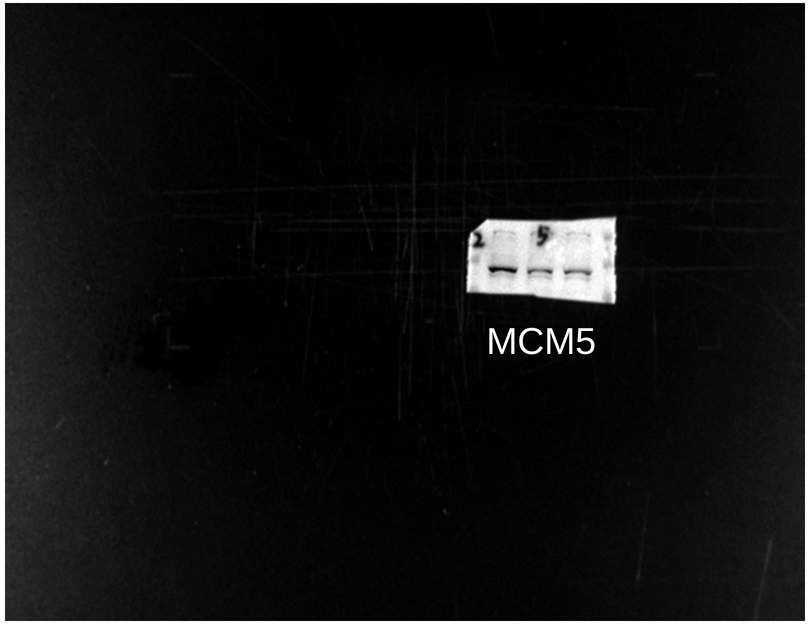

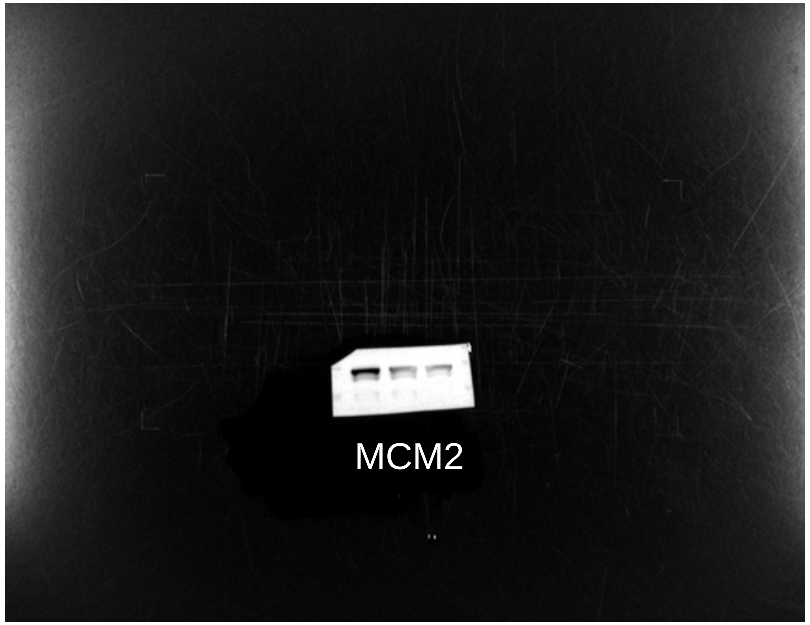

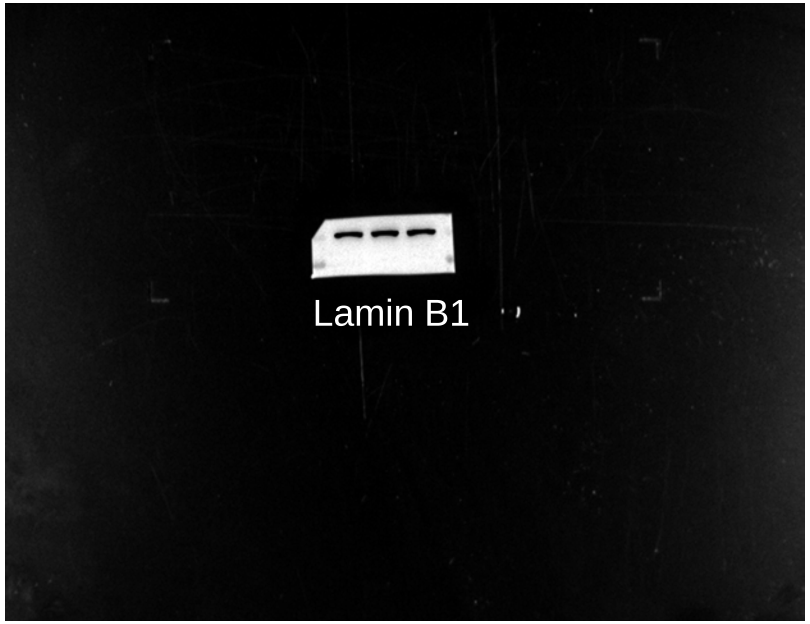

Figure 2F Chromatin HCT8

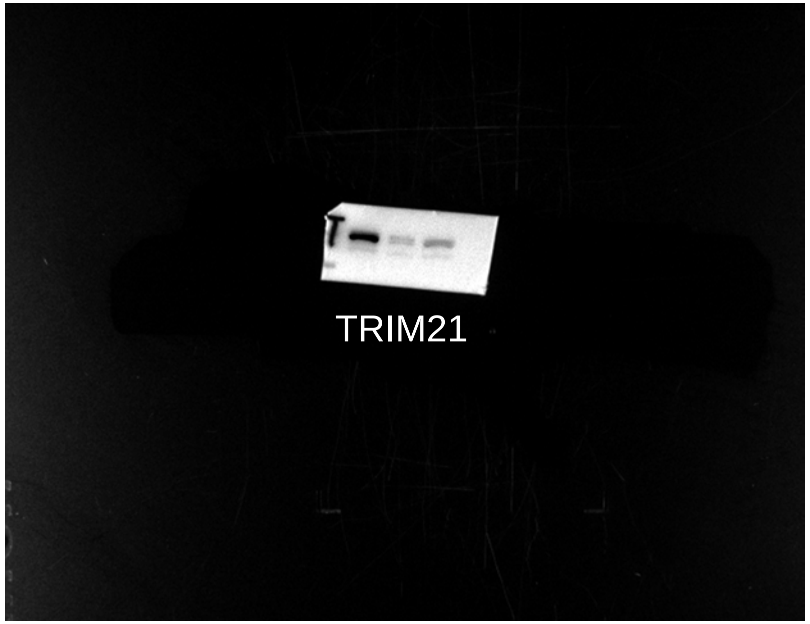

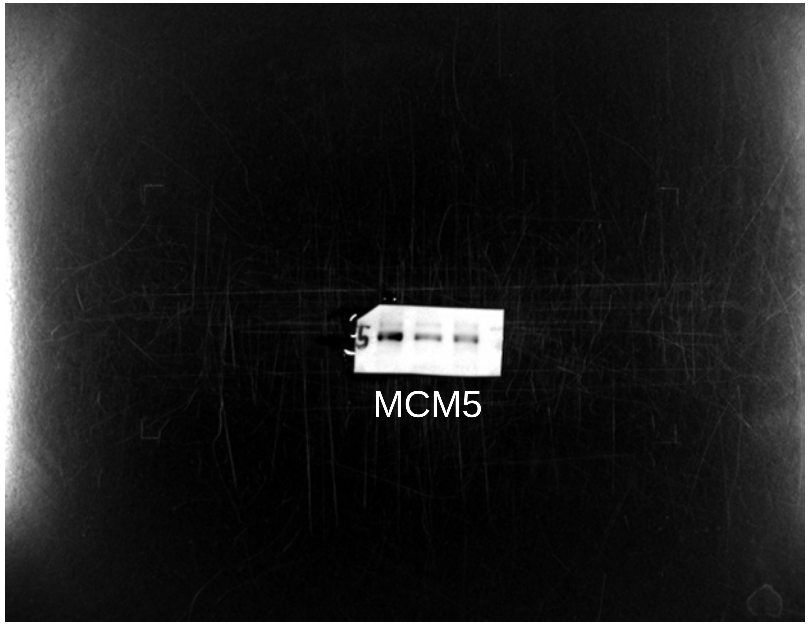

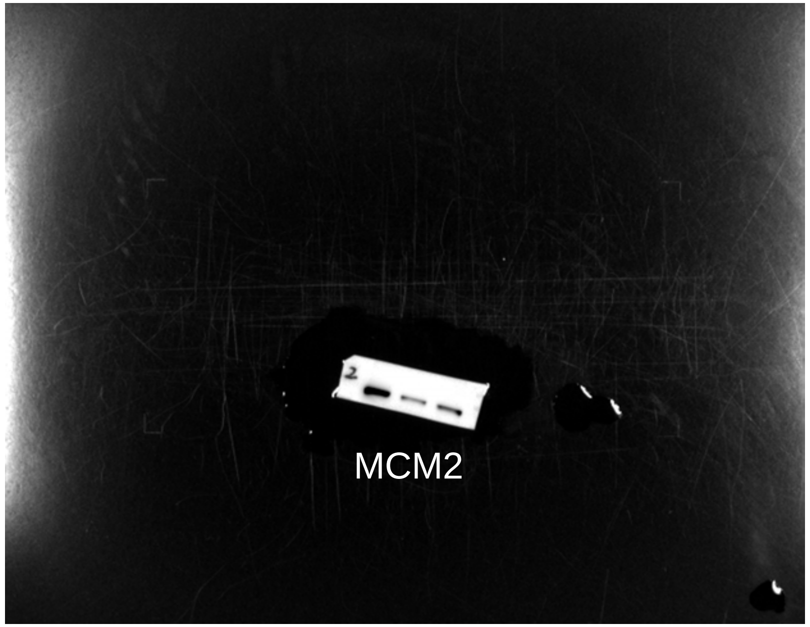

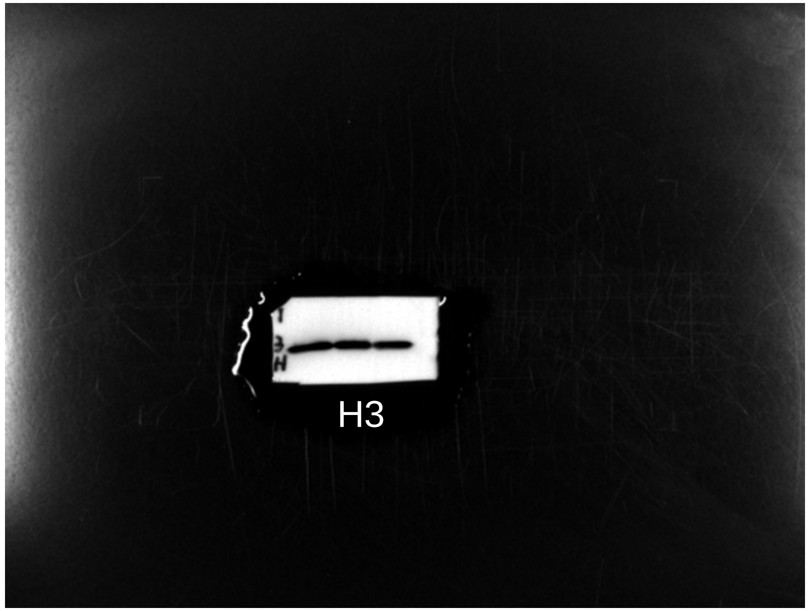

Figure 2F Whole HCT116

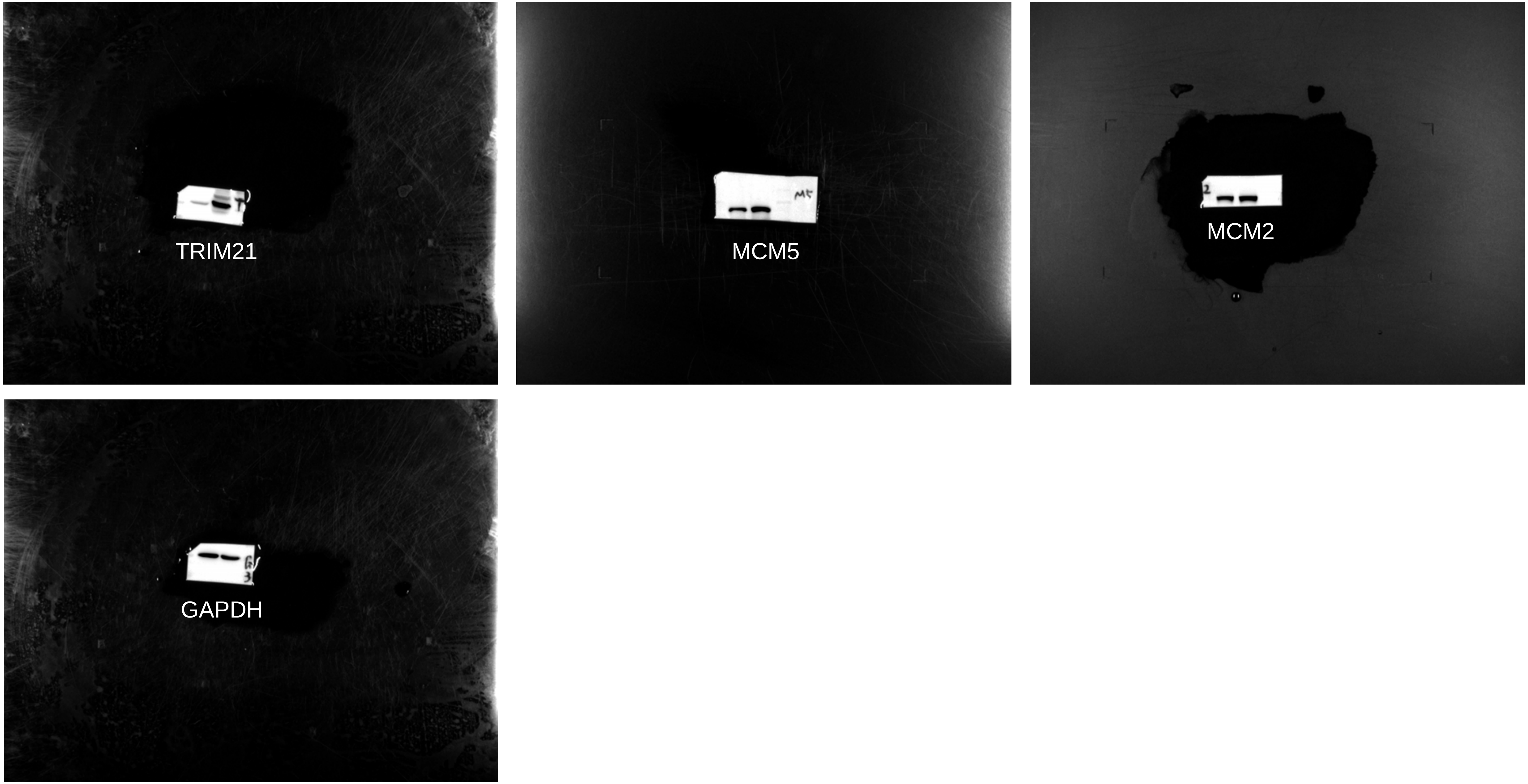

Figure 2F Nuclei HCT116

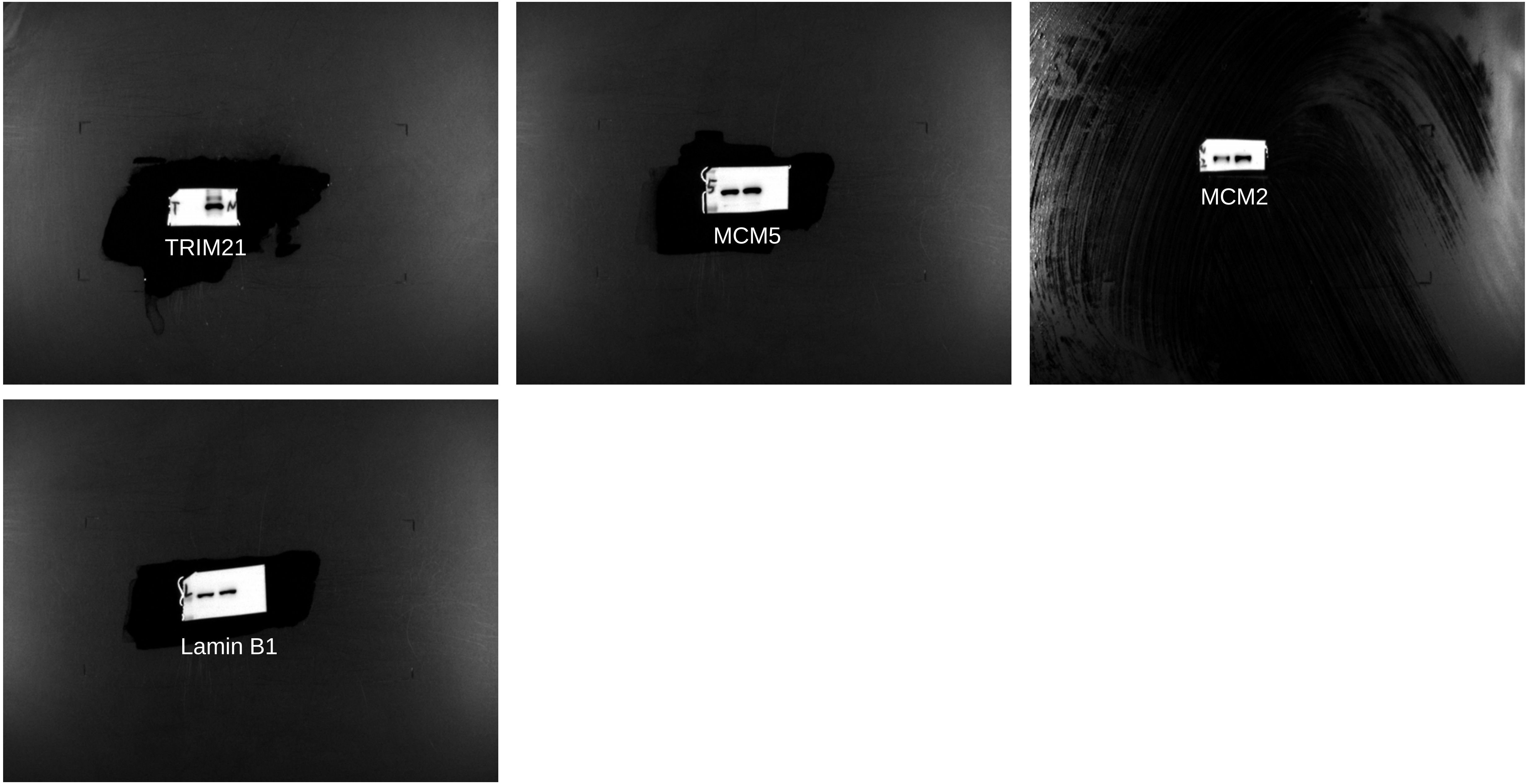

Figure 2F Chromatin HCT116

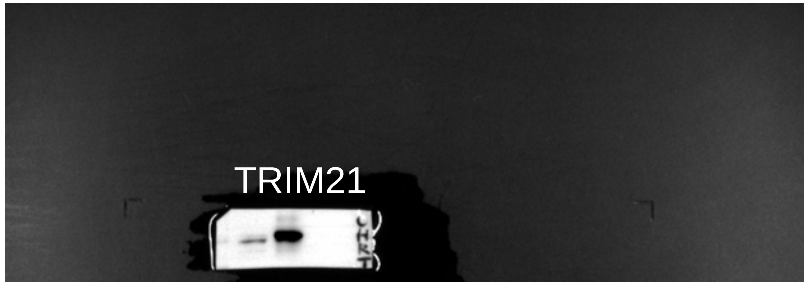

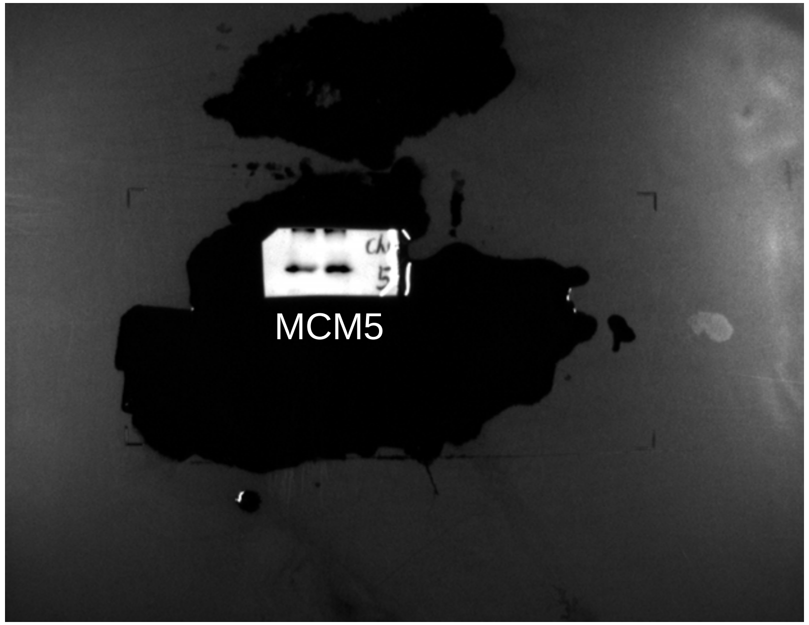

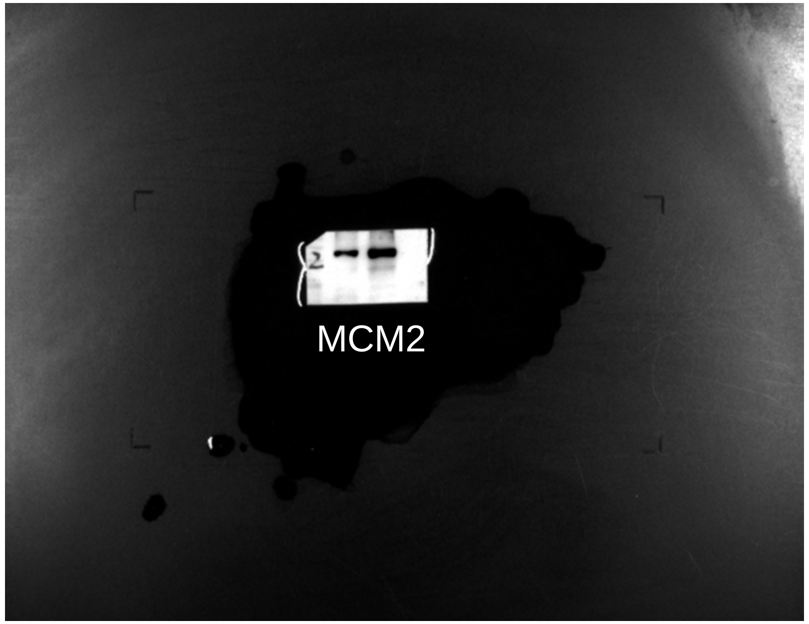

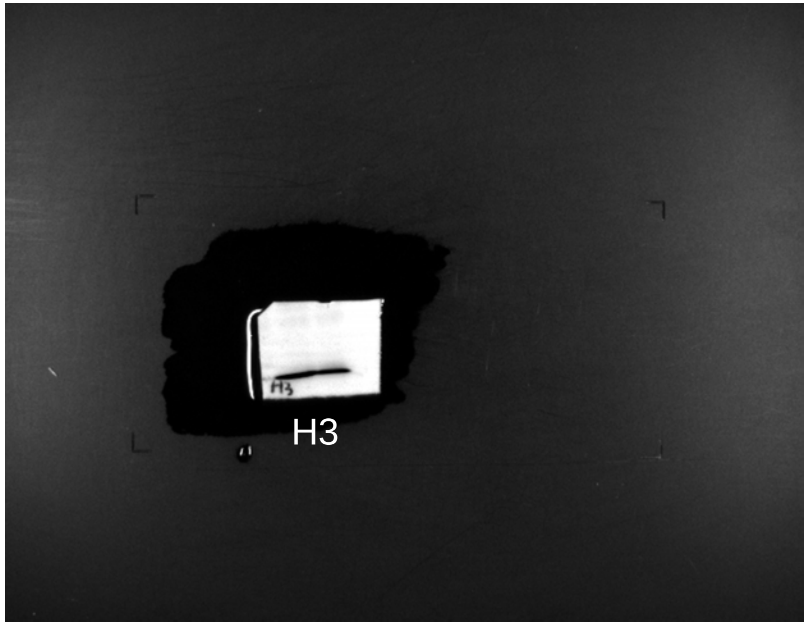

Figure 5A

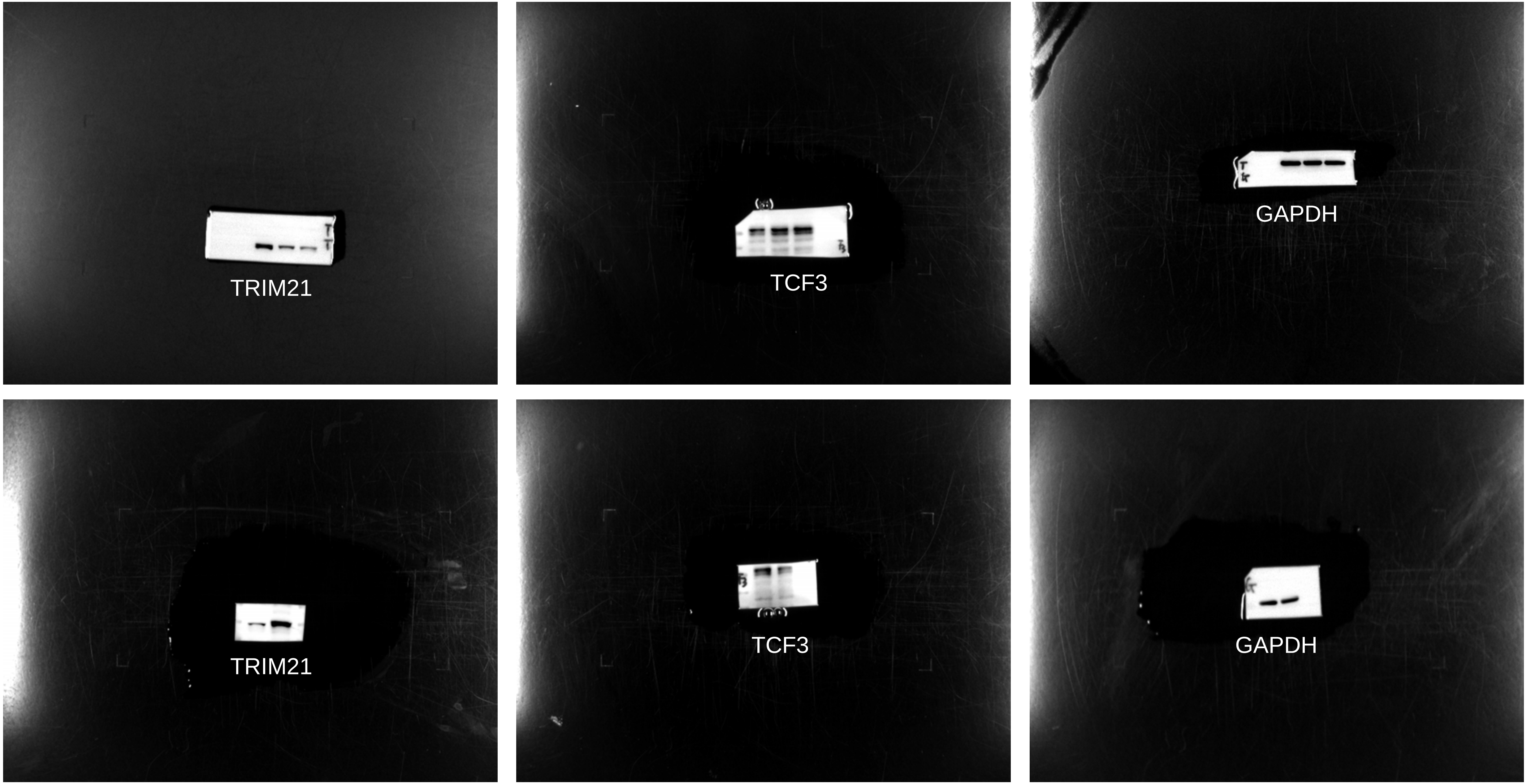

Figure 5B

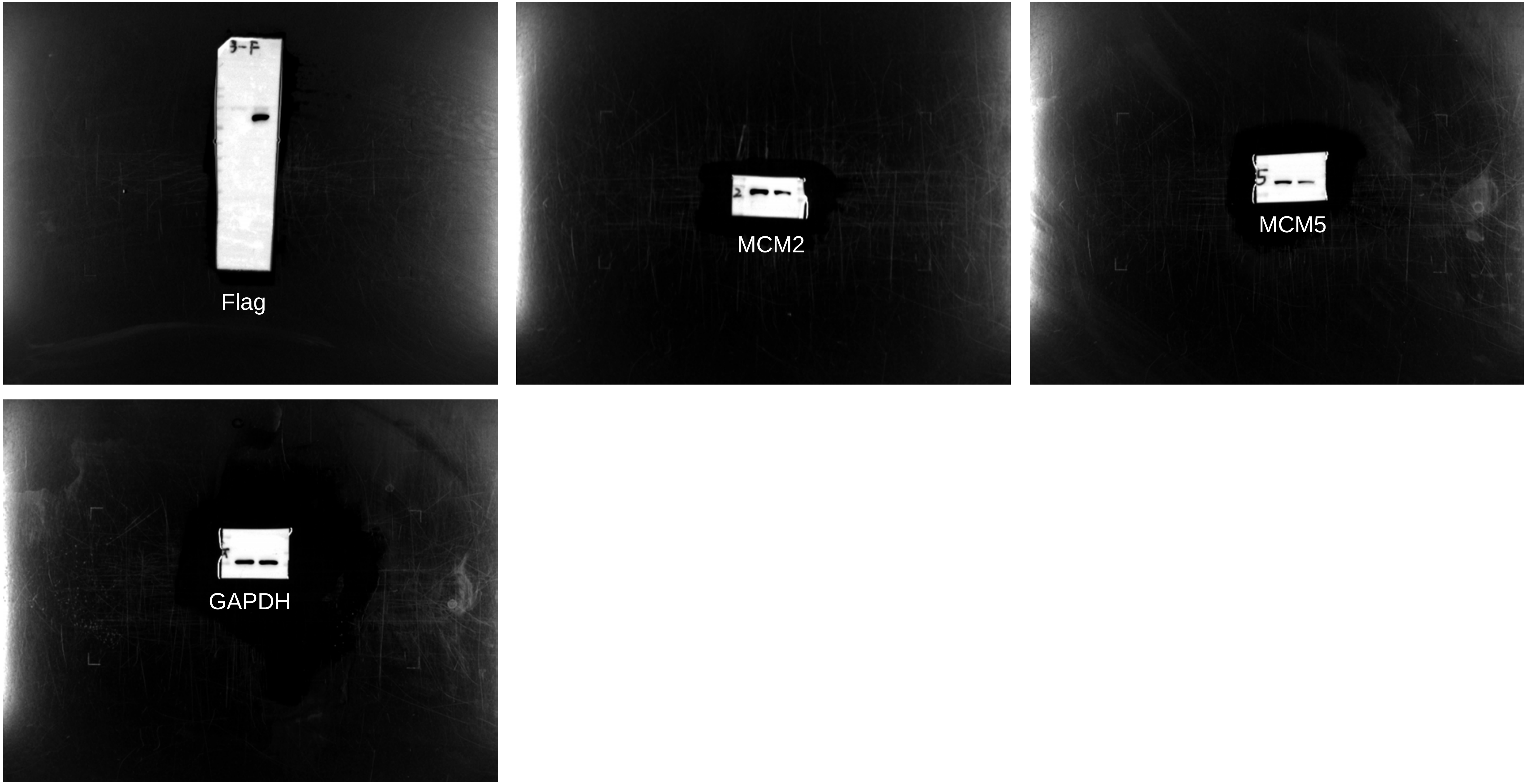

Figure 5B

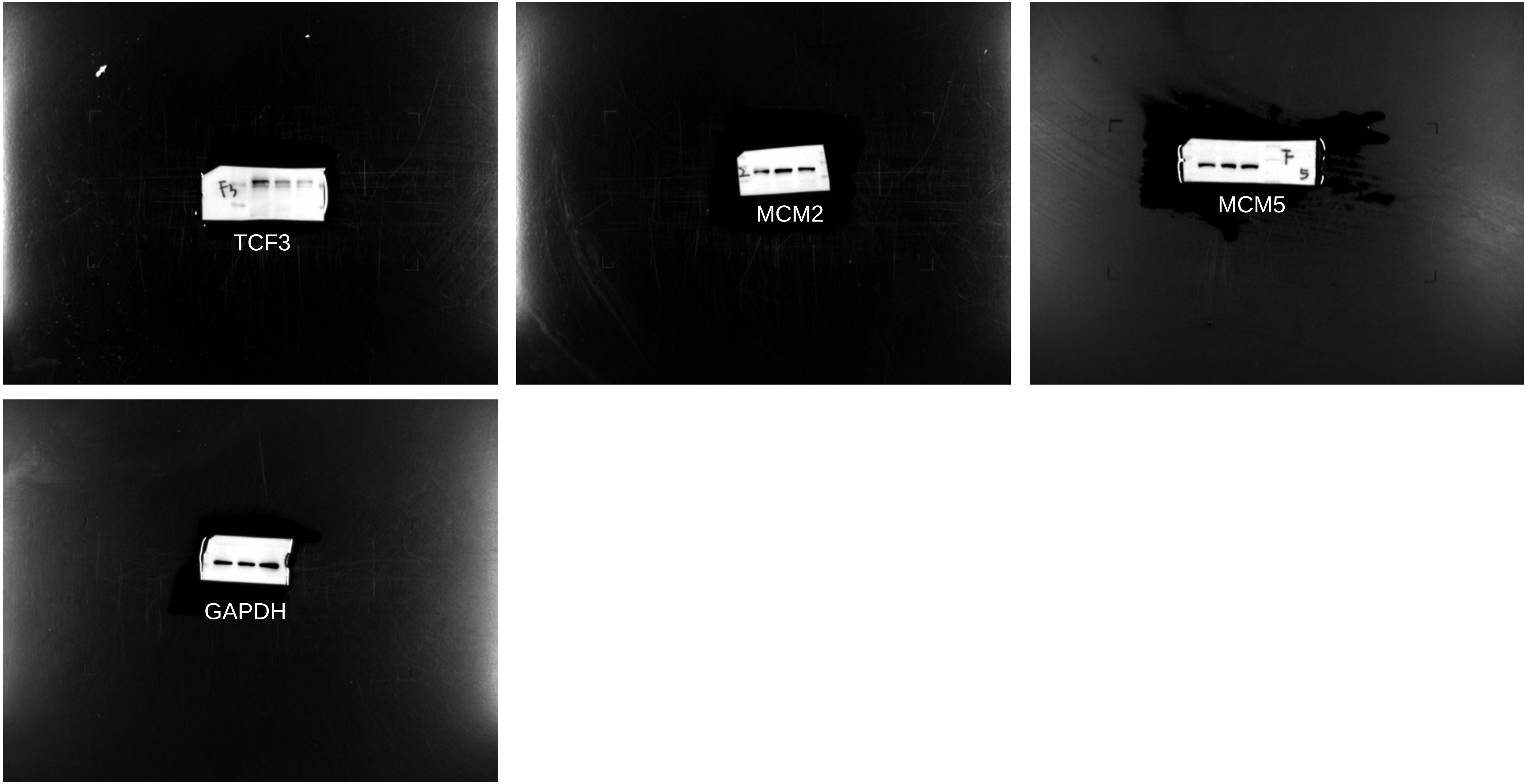

Figure 6B

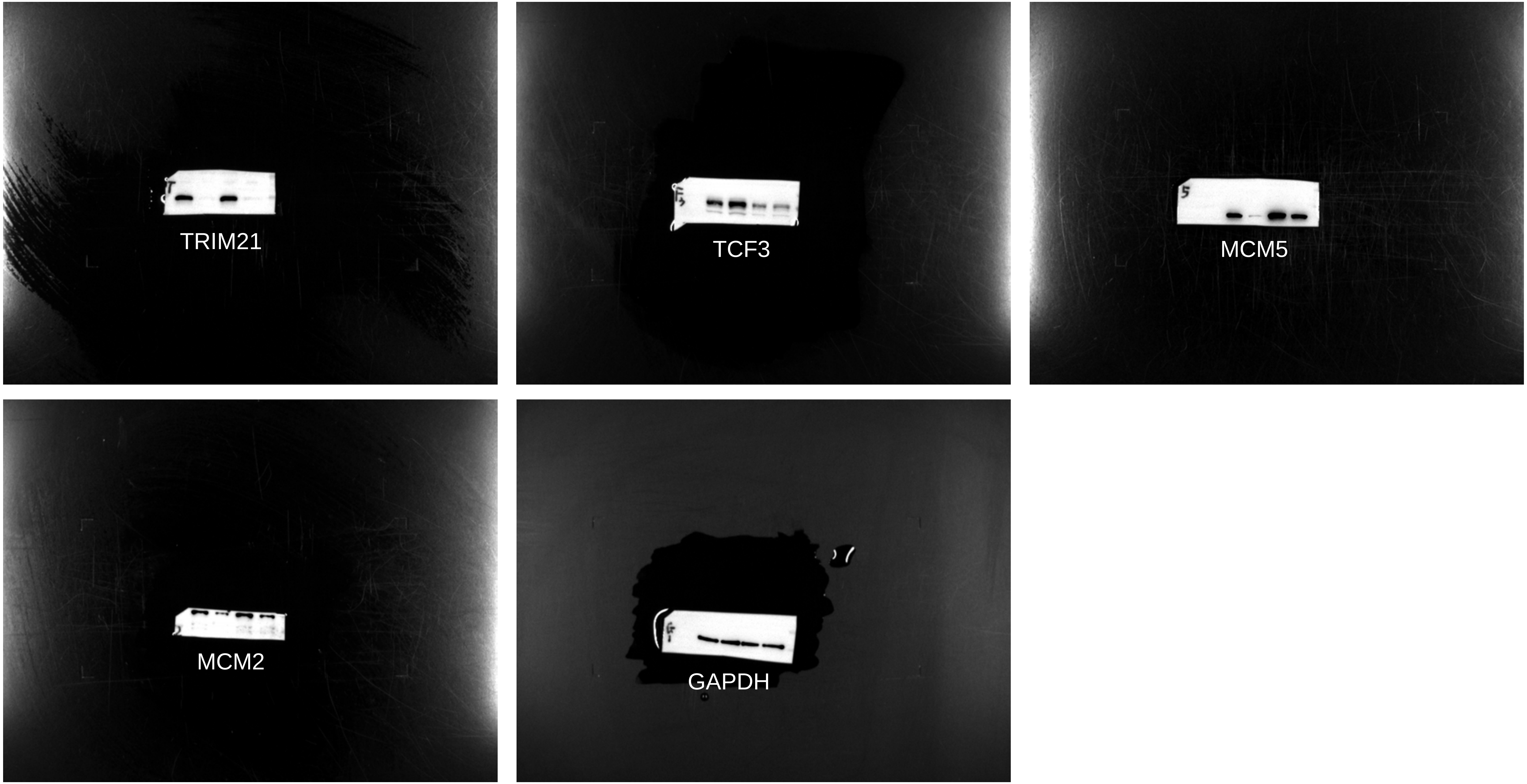

Supplementary Figure 1B

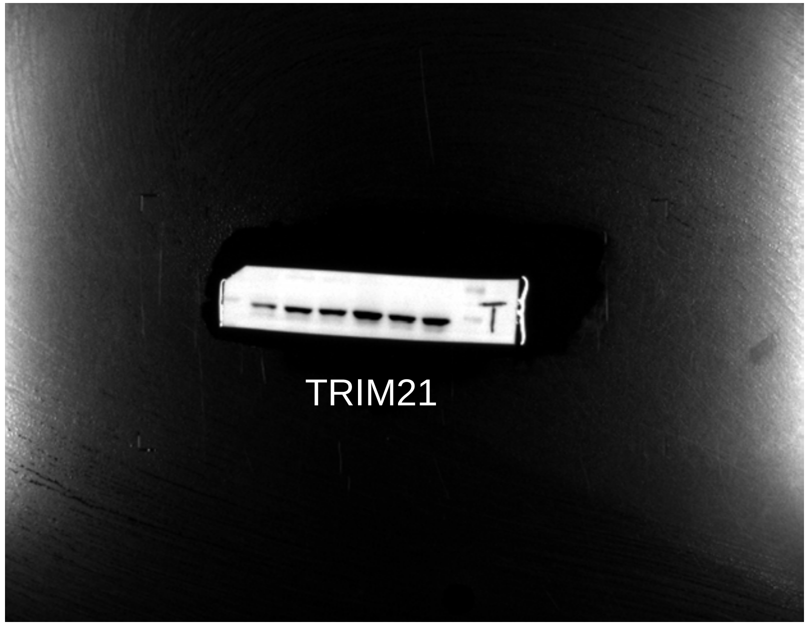

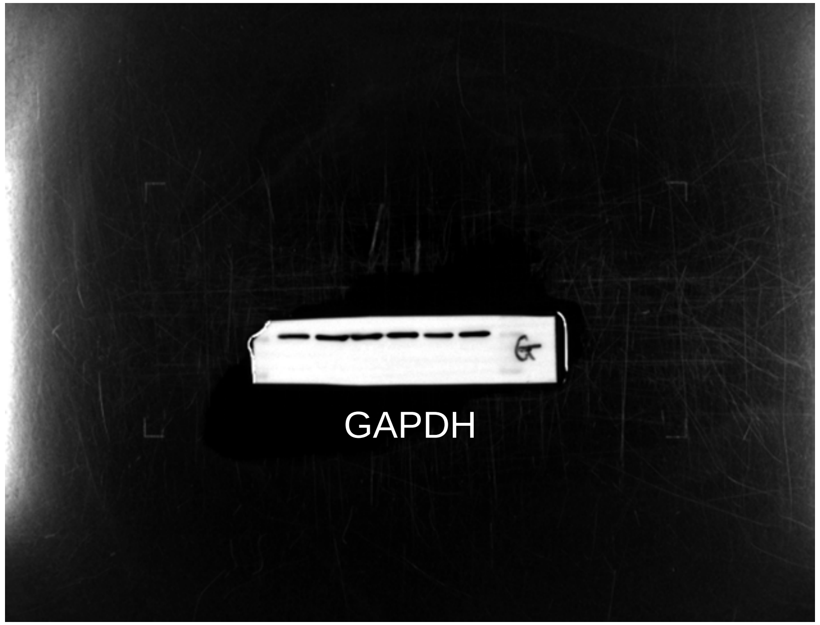

Supplementary Figure 1C

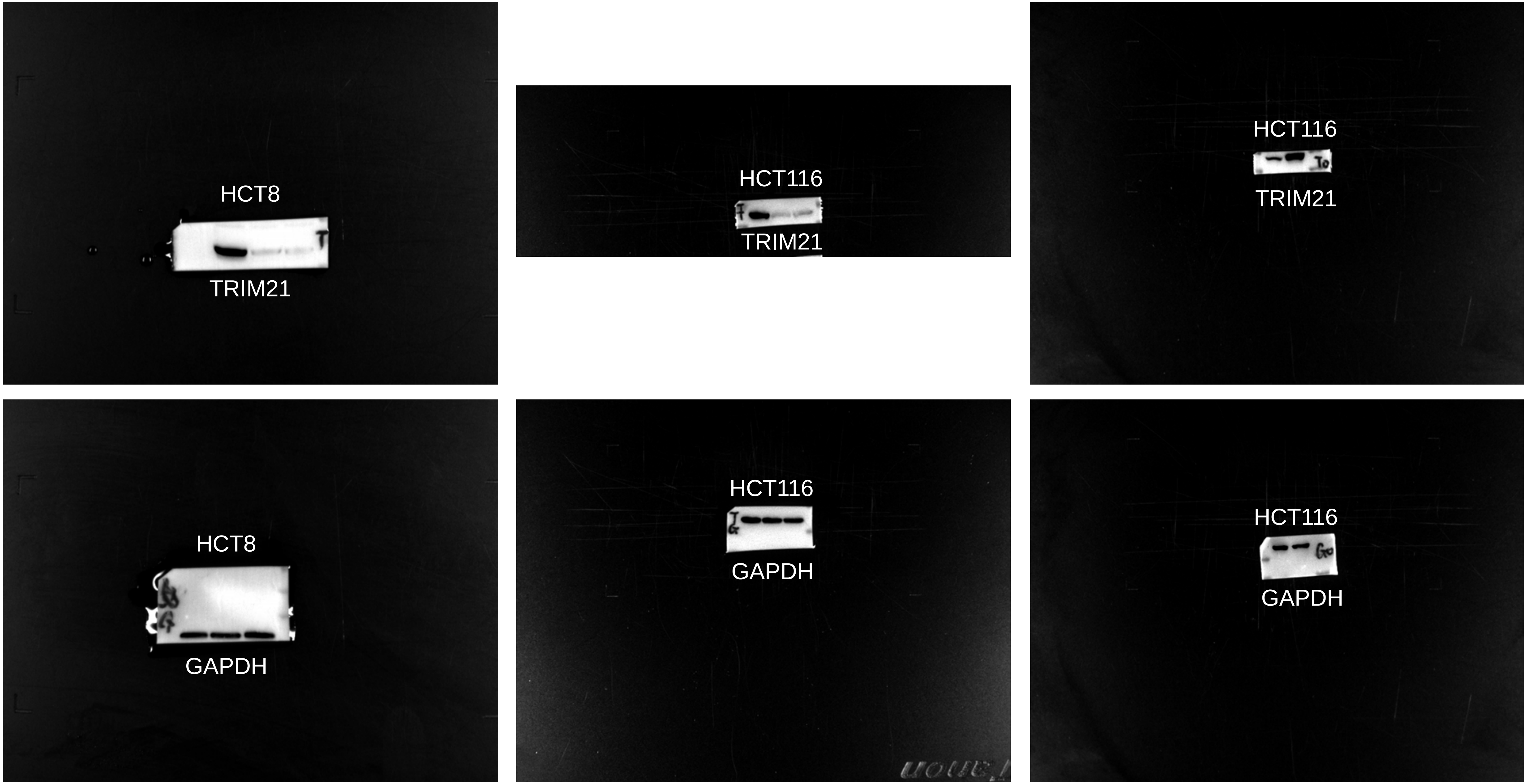

Supplementary Figure 1D

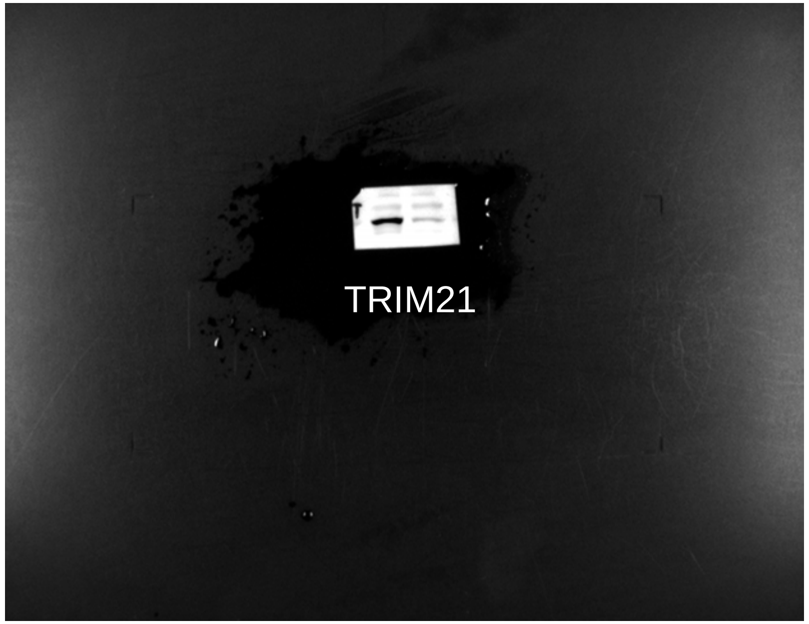

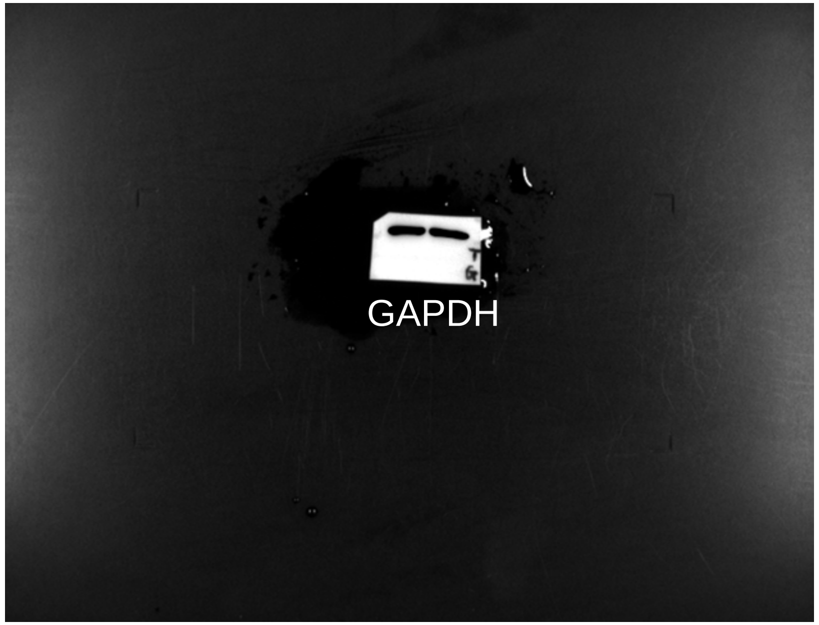

Supplementary Figure 3B

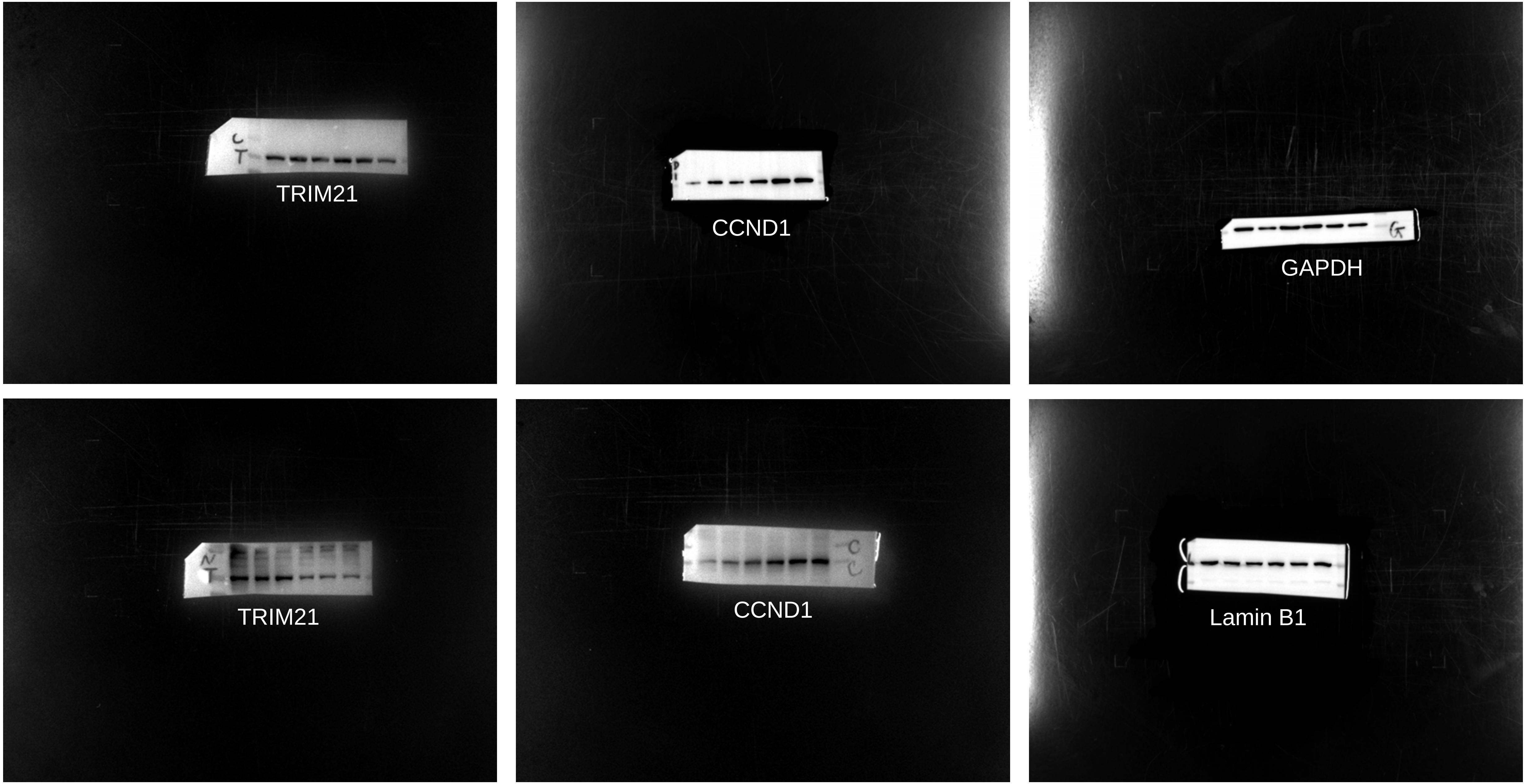

Supplementary Figure 3C

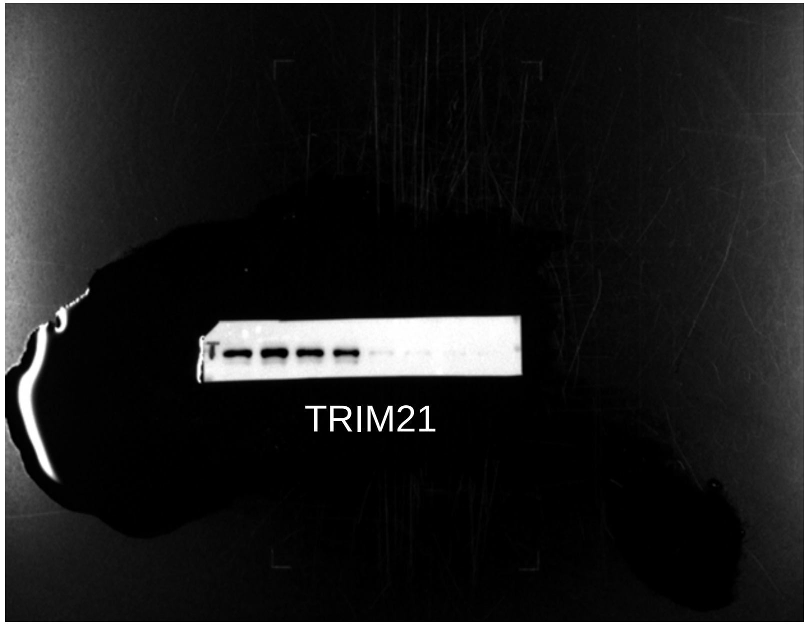

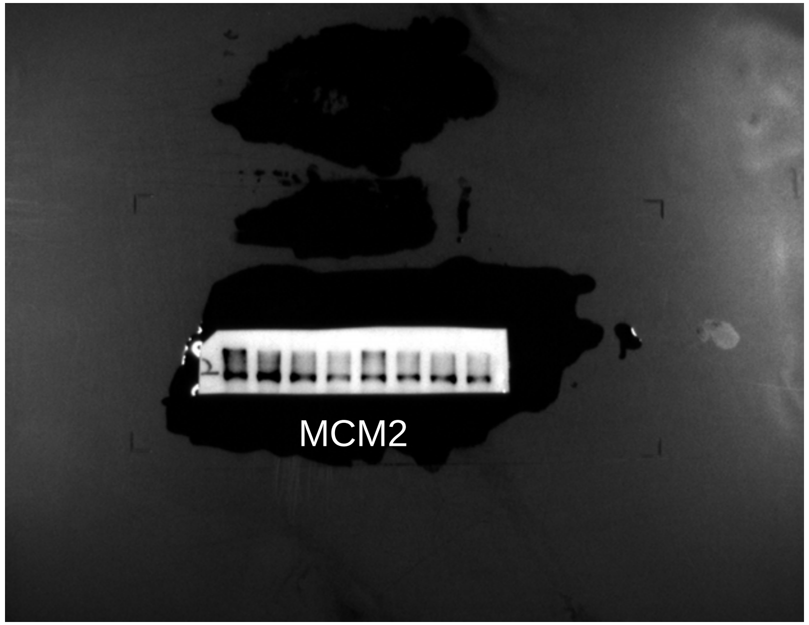

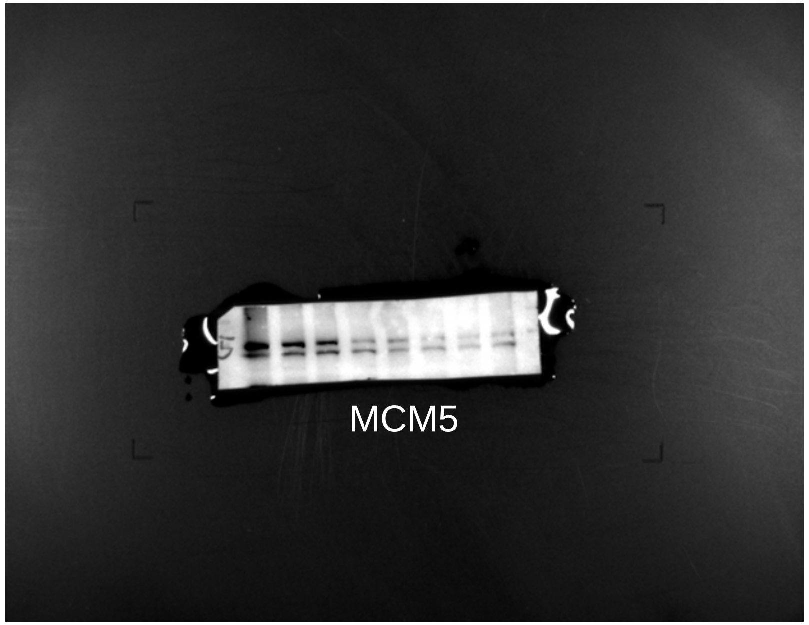

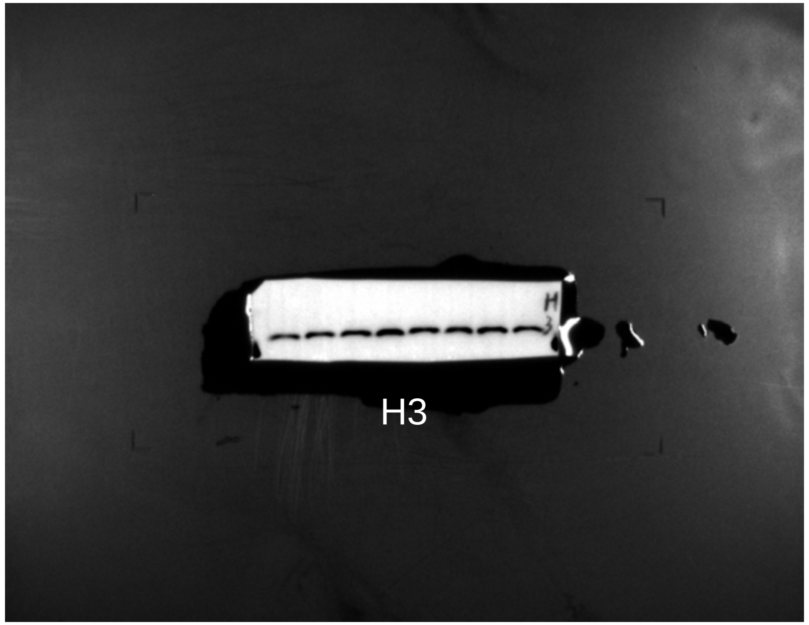

Supplementary Figure 3E

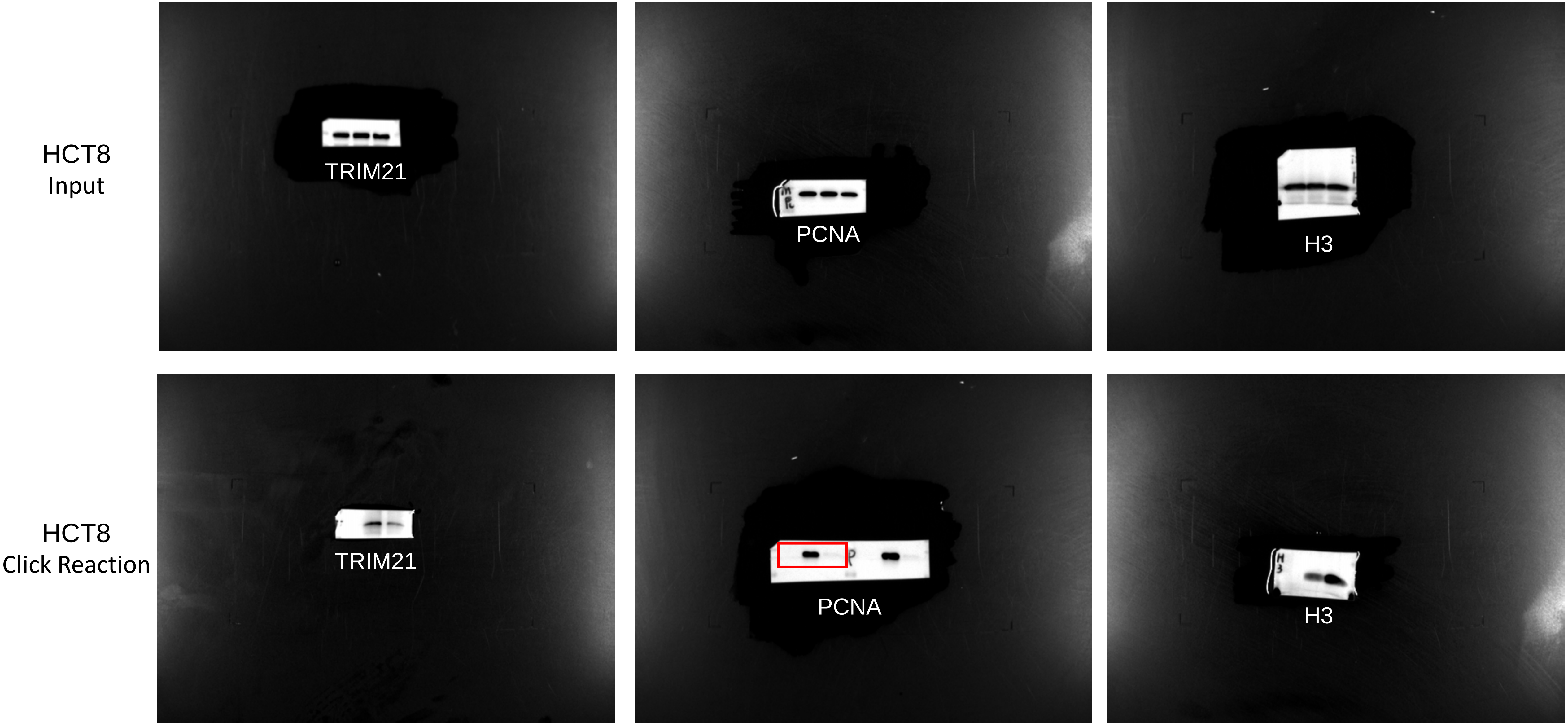

Supplementary Figure 3E

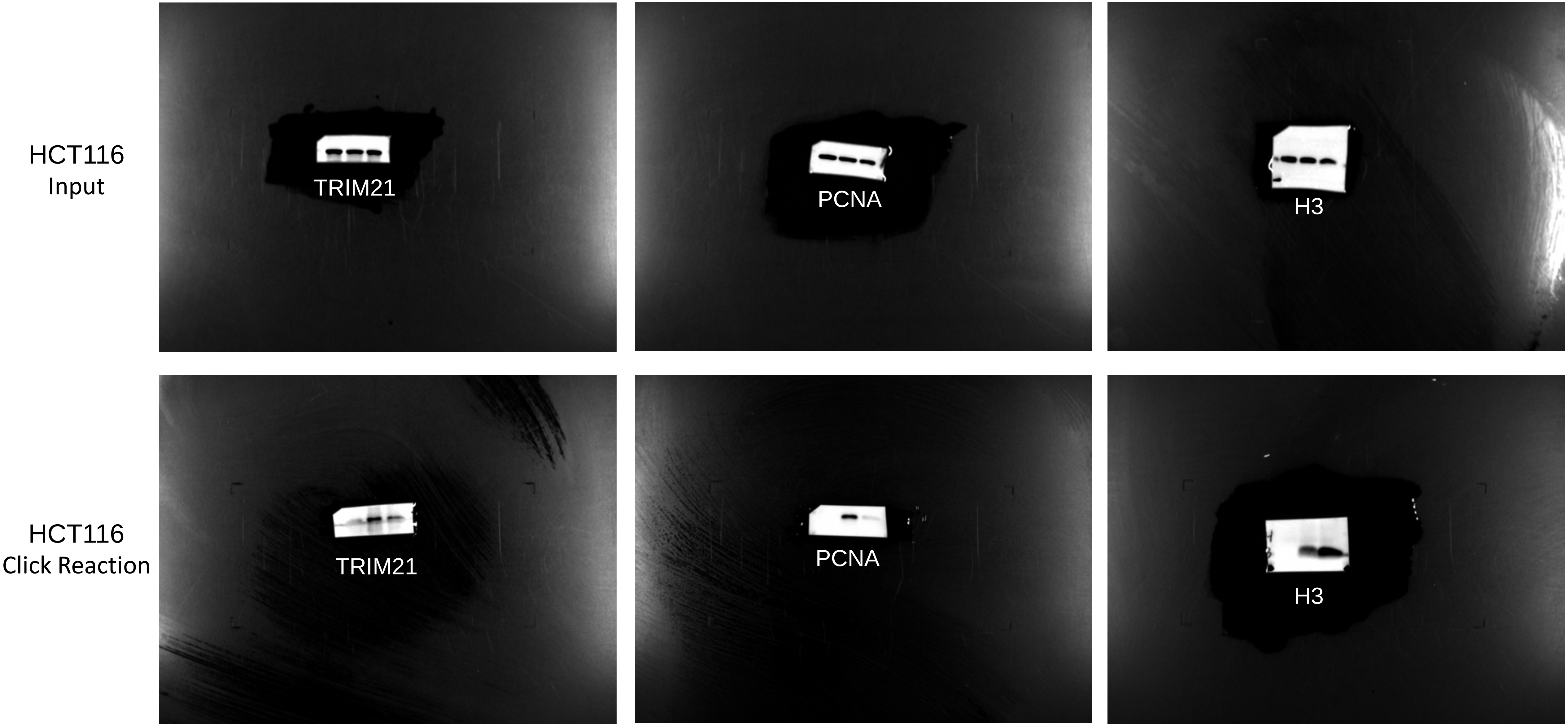

Supplementary Figure 4A

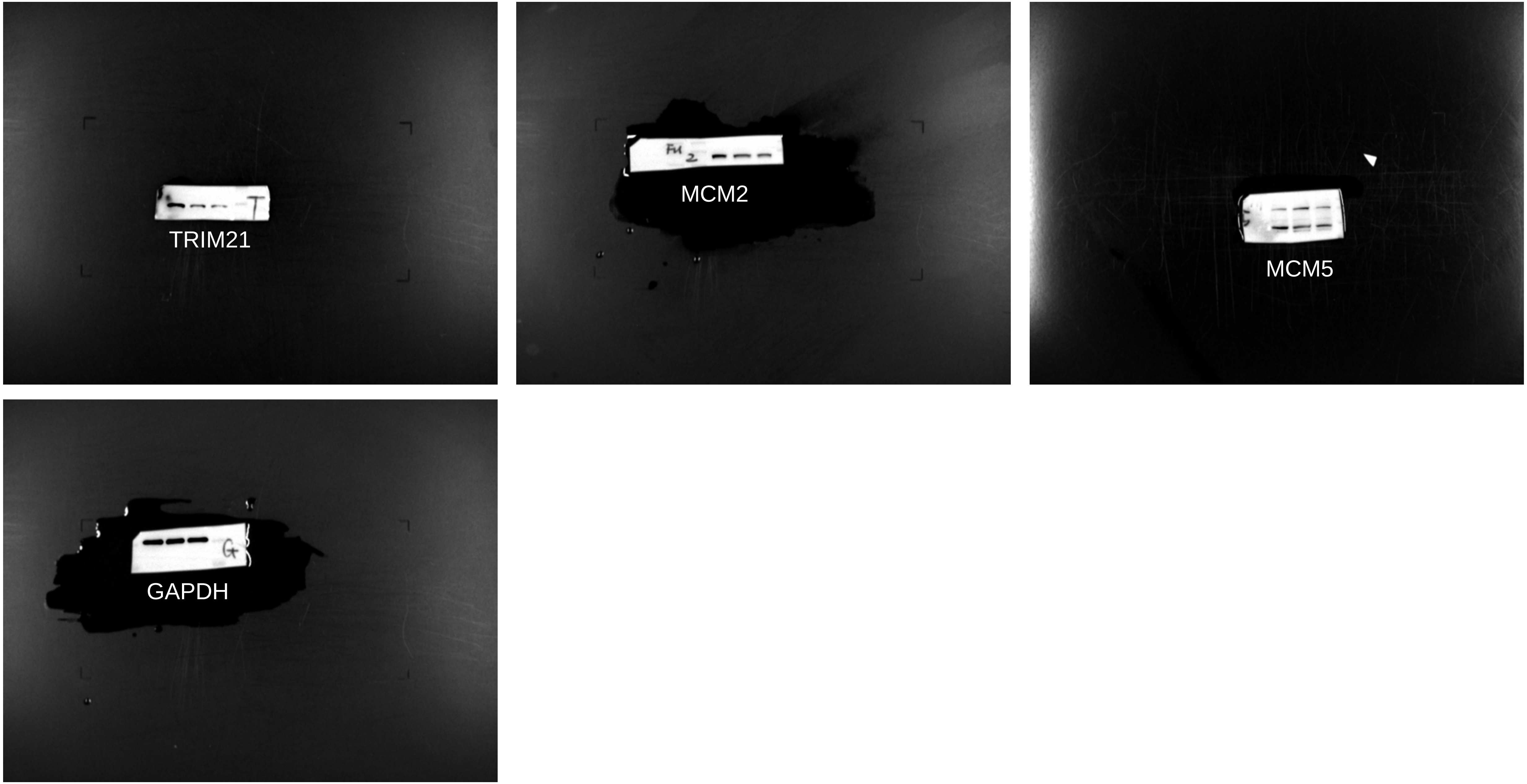

Supplementary Figure 5A

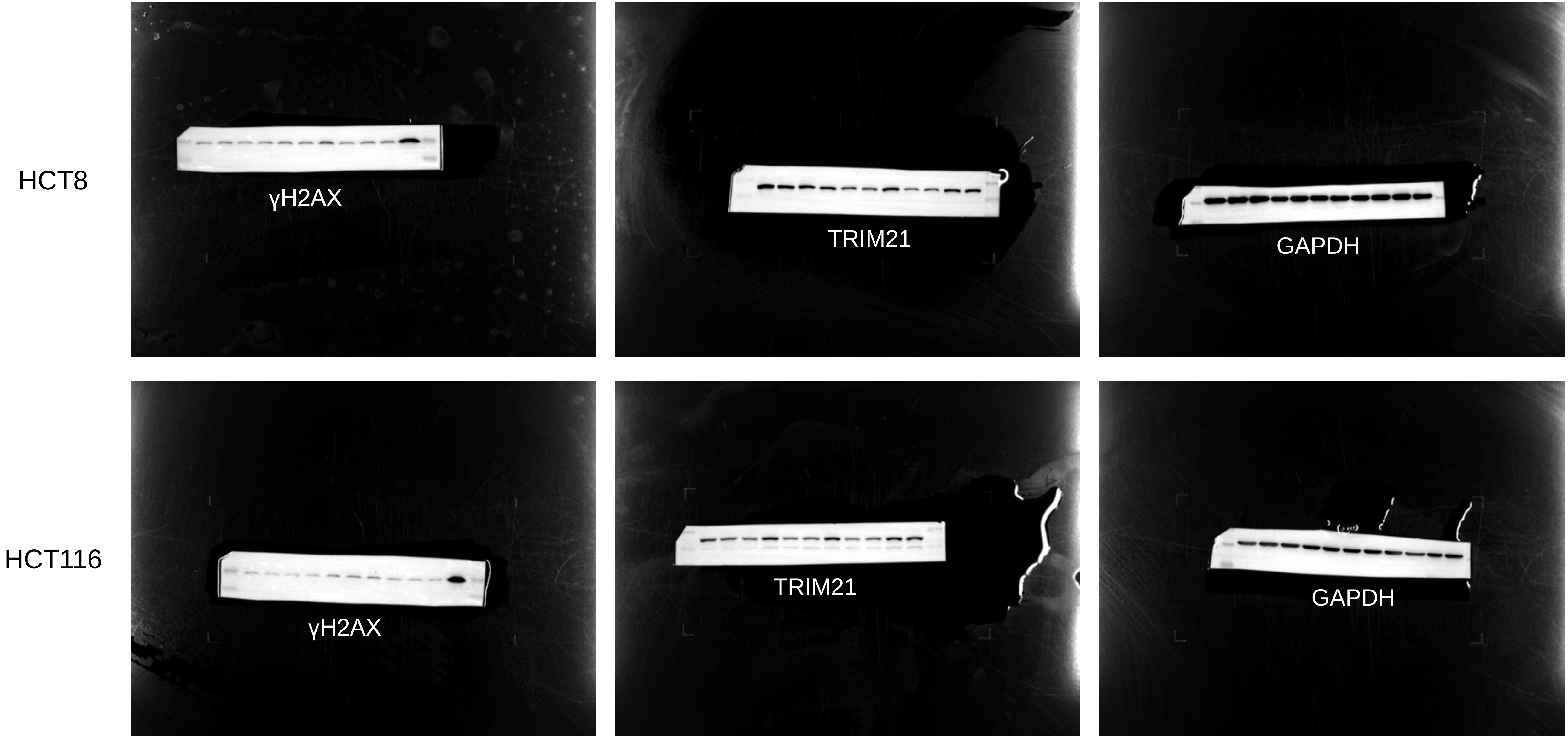

Supplementary Figure 5C

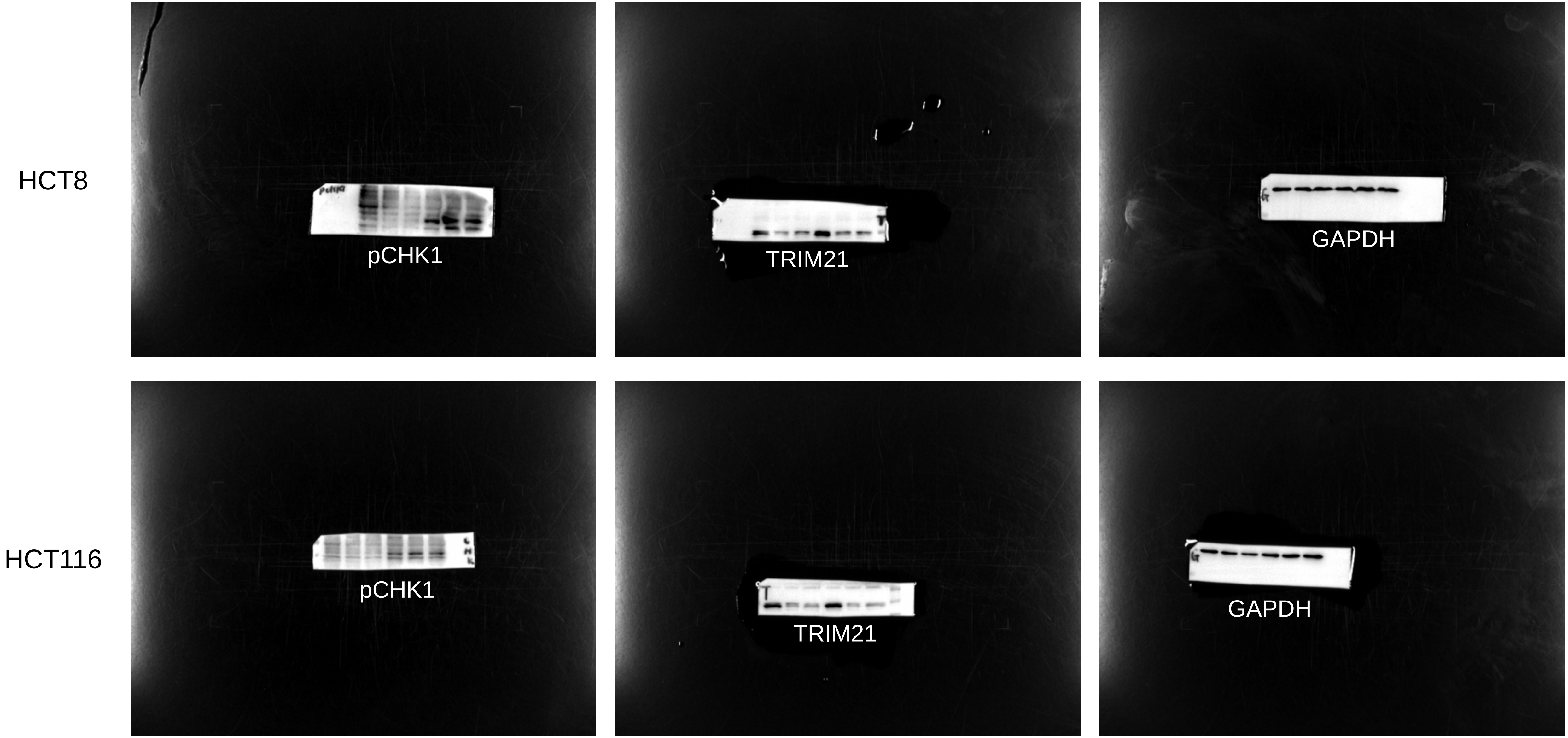

Supplementary Figure 6C

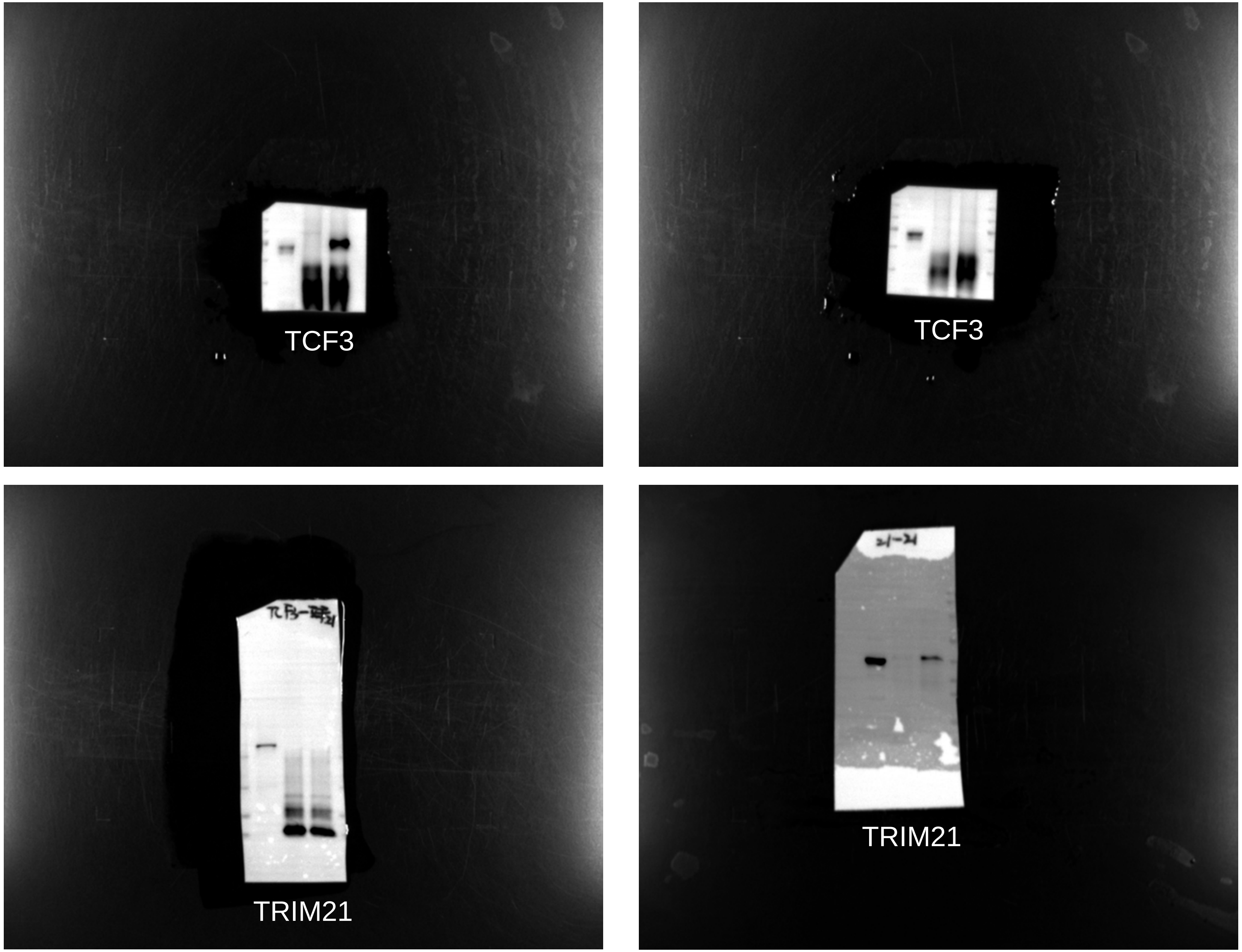

Supplementary Figure 6D

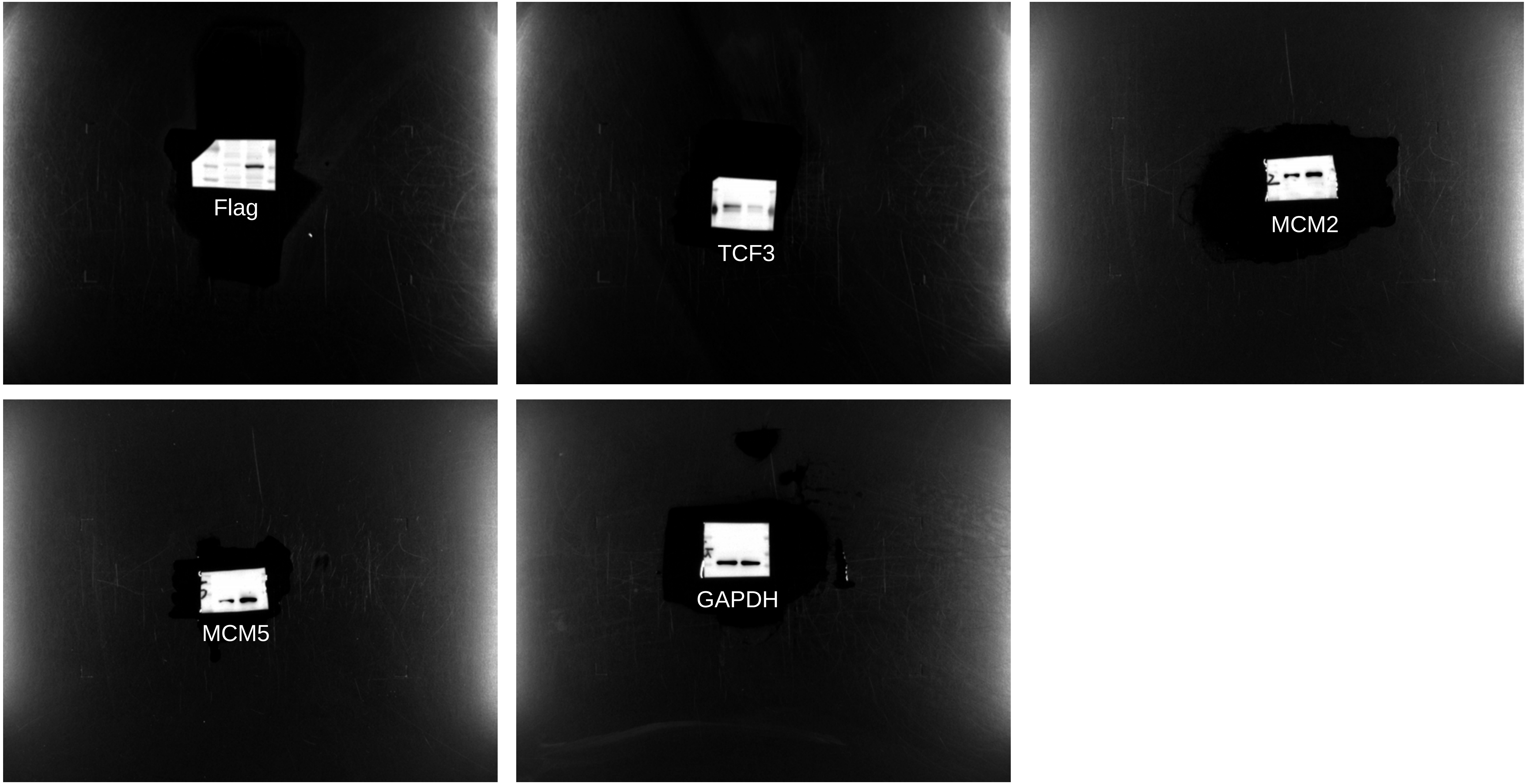

Supplementary Figure 6E

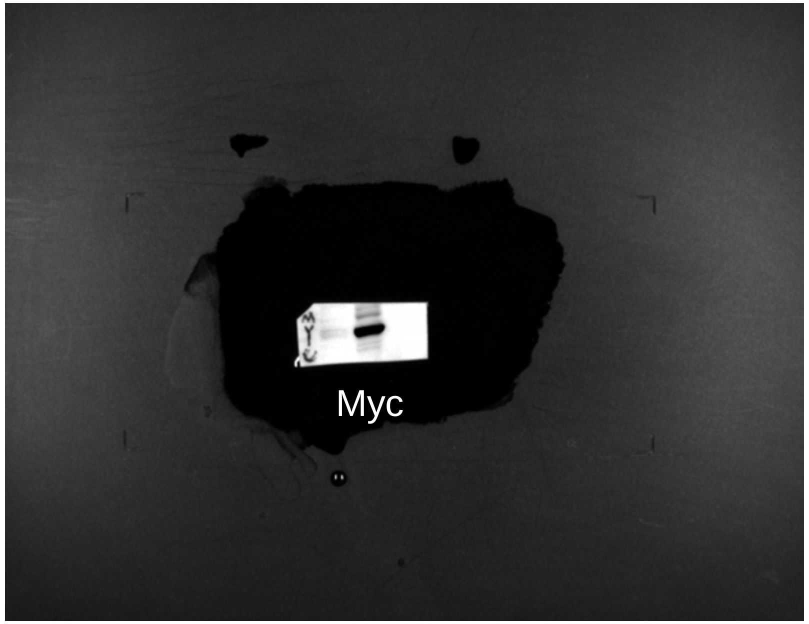

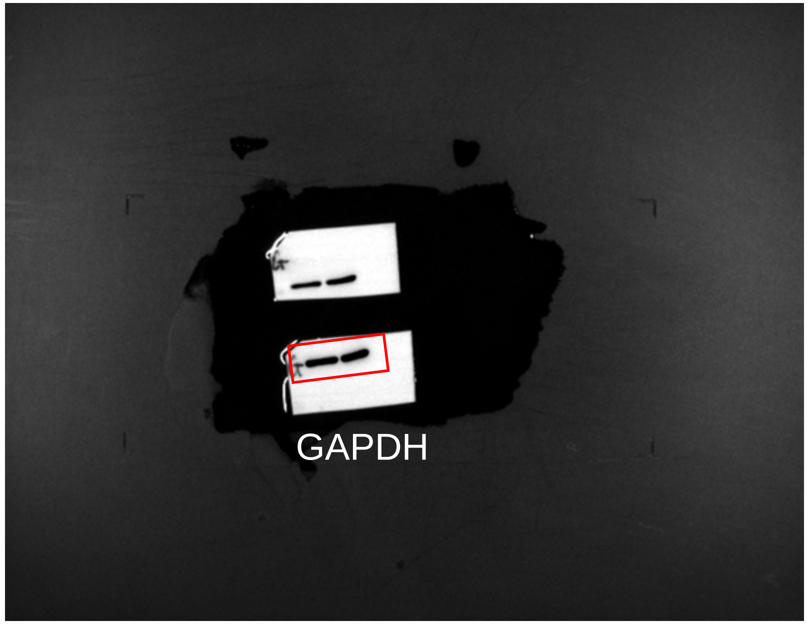

Supplementary Figure 7A

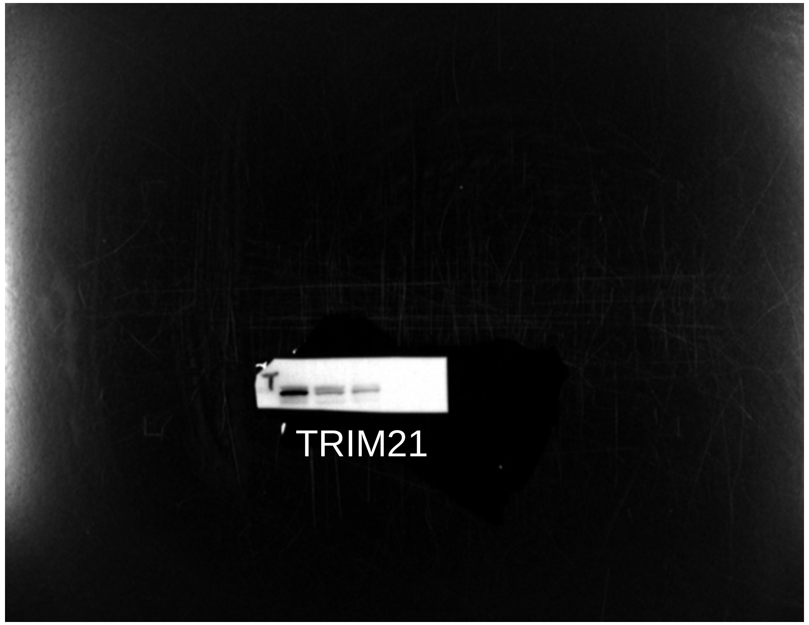

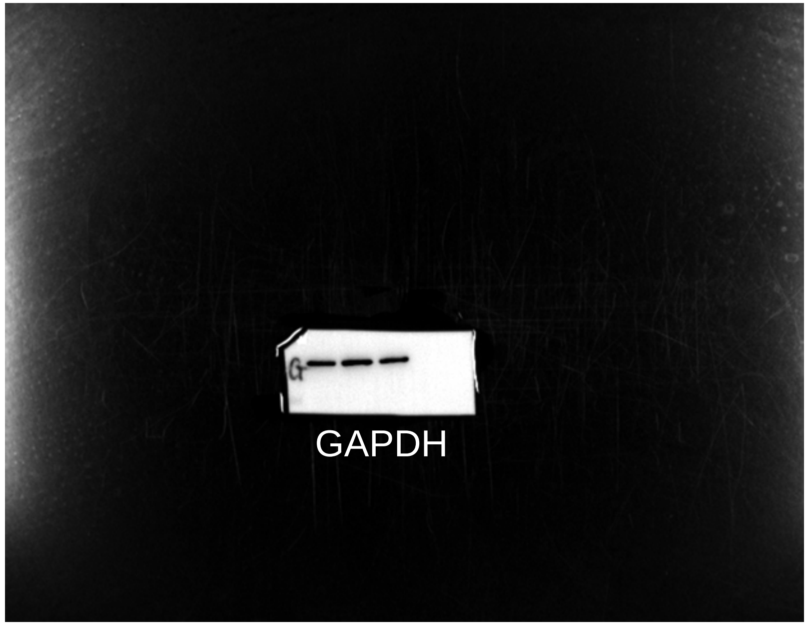

Supplementary Figure 7B

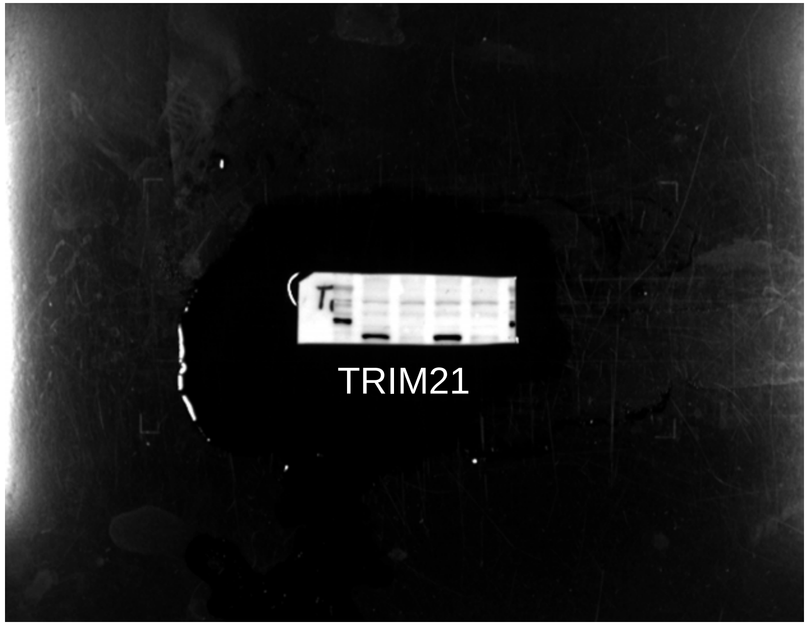

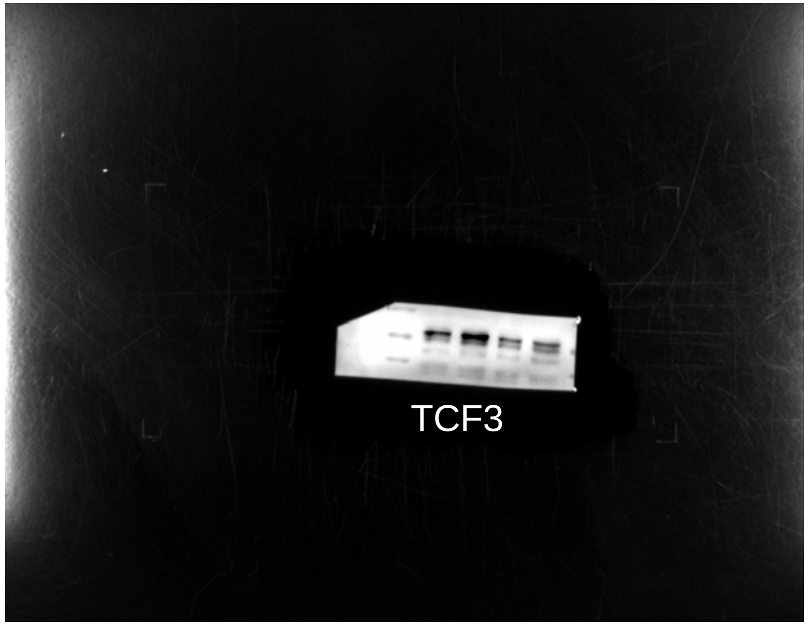

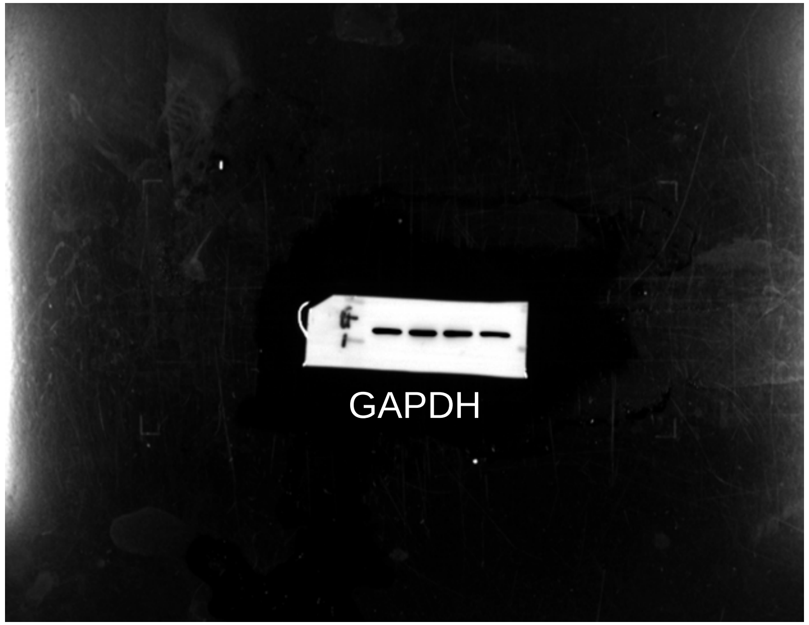

Supplement: Supplementary file 2 — Full and Uncropped Western Blots [file 41420_2025_2722_MOESM2_ESM.pdf]
